# Anti-inflammatory dopamine- and serotonin-based endocannabinoid epoxides reciprocally regulate cannabinoid receptors and the TRPV1 channel

**DOI:** 10.1038/s41467-021-20946-6

**Published:** 2021-02-10

**Authors:** William R. Arnold, Lauren N. Carnevale, Zili Xie, Javier L. Baylon, Emad Tajkhorshid, Hongzhen Hu, Aditi Das

**Affiliations:** 1 Department of Biochemistry, University of Illinois Urbana-Champaign, Urbana, IL 61801 USA; 2 Department of Anesthesiology, The Center for the Study of Itch & Sensory Disorders, Washington University School of Medicine, St. Louis, MO 63110 USA; 3 Department of Biochemistry, Center for Biophysics and Quantitative Biology, Center for Macromolecular Modeling and Bioinformatics, Beckman Institute for Advanced Science and Technology, University of Illinois Urbana-Champaign, Urbana, IL 61801 USA; 4 Department of Comparative Biosciences, Department of Biochemistry, Center for Biophysics and Quantitative Biology, Beckman Institute for Advanced Science and Technology, University of Illinois Urbana-Champaign, Urbana, IL USA; 5 Division of Nutritional Sciences, Neuroscience Program, Department of Bioengineering, Cancer Center at Illinois, University of Illinois Urbana-Champaign, Urbana, IL 61801 USA

**Keywords:** Biochemistry, Biophysics

## Abstract

The endocannabinoid system is a promising target to mitigate pain as the endocannabinoids are endogenous ligands of the pain-mediating receptors—cannabinoid receptors 1 and 2 (CB1 and CB2) and TRPV1. Herein, we report on a class of lipids formed by the epoxidation of N-arachidonoyl-dopamine (NADA) and N-arachidonoyl-serotonin (NA5HT) by epoxygenases. EpoNADA and epoNA5HT are dual-functional rheostat modulators of the endocannabinoid-TRPV1 axis. EpoNADA and epoNA5HT are stronger modulators of TRPV1 than either NADA or NA5HT, and epoNA5HT displays a significantly stronger inhibition on TRPV1-mediated responses in primary afferent neurons. Moreover, epoNA5HT is a full CB1 agonist. These epoxides reduce the pro-inflammatory biomarkers IL-6, IL-1β, TNF-α and nitrous oxide and raise anti-inflammatory IL-10 cytokine in activated microglial cells. The epoxides are spontaneously generated by activated microglia cells and their formation is potentiated in the presence of anandamide. Detailed kinetics and molecular dynamics simulation studies provide evidence for this potentiation using the epoxygenase human CYP2J2. Taken together, inflammation leads to an increase in the metabolism of NADA, NA5HT and other eCBs by epoxygenases to form the corresponding epoxides. The epoxide metabolites are bioactive lipids that are potent, multi-faceted molecules, capable of influencing the activity of CB1, CB2 and TRPV1 receptors.

## Abstract (web summary)

Endocannabinoids are ligands of cannabinoid receptors and a promising target for pain management. Here, the authors report a class of lipids formed by the epoxidation of N-arachidonoyl dopamine and N-arachidonoyl serotonin by cytochrome P450 epoxygenases, which reciprocally regulate canabinoid receptors and display anti-inflammatory activity.

## Introduction

Opioids are highly addictive pain medications that are susceptible for abuse. The age-adjusted death rate by opioid overdose was determined to be nearly 20 per 100,000 people in the United States in 2016, according to a report by the Centers for Disease Control and Prevention^1^. Hence, there is a need for therapeutic alternatives to opioids that combat inflammation and the associated pain.

Pain is regulated primarily by sensory afferent neurons and immune cells. Both of these cell types are rich sources of lipid mediators. Lipid mediators are generated via the enzymatic oxidation of dietary omega-3 and omega-6 polyunsaturated fatty acids (PUFAs). The pro-inflammatory lipid mediators contribute to pain sensitivity by activating the GPCRs in the sensory neurons to increase membrane excitability and pain response^2^. Non-steroidal anti-inflammatory drugs (NSAIDs) are used to inhibit cyclooxygenases, leading to a decrease in the synthesis of pro-inflammatory lipid metabolites such as prostaglandin E_2_ (PGE_2_), thereby decreasing inflammatory pain^3^. On the other hand, anti-inflammatory and pro-resolving lipid mediators suppress and resolve the inflammatory process, and thus attenuate inflammatory pain^4,5^. Hence, lipid mediators can fine-tune the pain response and have been at the center for the development of alternative non-opioid pain therapeutics^6^.

Additionally, cannabis has been used for centuries to reduce nociceptive pain either alone or in combination with opioids^7^. The primary components of cannabis interact in the body with cannabinoid receptors 1 and 2 (CB1 and CB2) and other GPCRs^8,9^. An endogenous class of bioactive lipids, known as endocannabinoids (eCBs), activates CB receptors and suppresses inflammation and pain sensitization^10^. The eCBs are derivatives of dietary omega-3 and omega-6 PUFAs and are generated by damaged neurons and inflamed tissues. CB1 receptors are highly expressed in the central nervous system, mostly in the presynaptic region, and there is substantial CB1 expression in the nociceptive sensory neurons. It has been shown that under different pain conditions there is a concomitant increase in CB1 expression^11^. Hence, there is sufficient evidence that CB1 mediates the psychotropic effects of cannabinoids such as modulating nociceptive pain, as well as modulating inflammation^8,12^. CB2 is mostly expressed in immune cells and mediates the anti-inflammatory effects of cannabinoids, which indirectly contributes to the anti-nociception of acute inflammatory pain^12,13^. CB2 receptor activation exerts profound anti-nociceptive effects in animal models of acute, inflammatory, and neuropathic pain^14^.

In addition to CB1 and CB2, a subclass of the eCBs act through transient receptor potential vanilloid 1 (TRPV1)^15^. TRPV1 is a non-selective cation channel that is activated by noxious temperatures, pH, and inflammatory agents^16,17^. TRPV1 often spearheads nociceptive pain signaling, and thus antagonizing TRPV1 can reduce pain. Paradoxically, the activation of TRPV1 by small molecules such as capsaicin (CAP), the spicy component of chili peppers, can also alleviate pain by desensitizing TRPV1 signaling^18^. TRPV1 also exhibits pro- and anti-inflammatory effects^19^.

Recently, it has been postulated that there is a crosstalk between CB1 and TRPV1 receptors, which are co-localized in dorsal root ganglion (DRG) and in neuron-enriched mesencephalic cultures, hippocampus, and cerebellum^20^. Therefore, the eCB system and TRPV1 axis provides a promising target to develop pain and inflammation therapeutics. For example, CMX-020 (Patent US8658632B2) is a novel drug based on the structure of eCBs and is in development to alleviate pain by binding to both cannabinoid receptors and TRPV1. Endogenous molecules and their synthetic derivatives may provide insight into effective therapeutic strategies with which to target the eCB-TRPV1 axis.

**Fig. 1:**
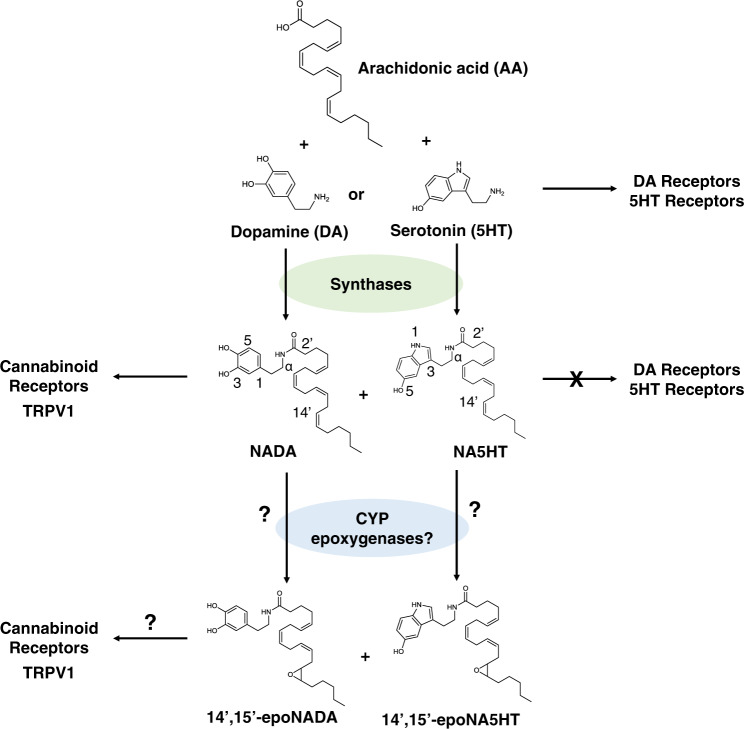
The endovanilloid (eVD) pathway and structures of 14’,15’-epoNADA and 14’,15’-epoNA5HT. NADA and NA5HT are synthesized by fatty acid amid hydrolase and other unidentified enzymes by the conjugation of arachidonic acid (AA) with dopamine (DA) or serotonin (5HT), respectively. NADA and NA5HT are known modulators of cannabinoid receptors and TRPV1; however, they do not activate DA or 5HT receptors. Herein, we study the epoxidation of NADA and NA5HT by CYP epoxygenases and test their effects on cannabinoid receptors and TRPV1.

The best-studied eCB is anandamide (N-arachidonoyl-ethanolamine: AEA), which is derived from the omega-6 PUFA arachidonic acid (AA)^21^. Besides AEA, other eCBs such as N-arachidonoyl-dopamine (NADA) and N-arachidonoyl-serotonin (NA5HT) are also derivatives of AA (Fig. 1). These dopamine^22,23^ and serotonin^24,25^ derivatives were identified in vivo in brain and intestinal tissues. It was shown that NADA binds with a higher affinity to CB1 than to CB2^22^; however, conclusive evidence for NA5HT binding to CB1/2 has yet to emerge. Additionally, NADA was shown to be an agonist of TRPV1^26^ and NA5HT is an antagonist of TRPV1, which results in analgesia^27^. Hence, NADA and NA5HT are classified as endovanilloids (eVDs) for their actions at the TRPV1 (vanilloid) receptor. Interestingly, NADA has low-affinity binding to DA receptors^22^, and the stimulation of degranulation in mast cells by NA5HT suggests it does not activate the 5HT receptors^28^. Therefore, NADA and NA5HT do not display typical DA or 5HT responses and are instead regulators of TRPV1.

To add to the complexity of lipid metabolism, cytochromes P450 (CYPs) are known to epoxidize lipids into anti-inflammatory and anti-pain mediators that are more effective than the parent molecules. For example, CYPs convert AA and omega-3 PUFAs into epoxy-PUFAs that have been shown to decrease pain^29,30^. Recently, the CYP-mediated metabolism of eCBs was shown to produce epoxy-eCBs that exhibit CB2 receptor selectivity and are anti-inflammatory and anti-tumorigenic^31–33^. For instance, CYPs epoxidize AEA into epoxyeicosatrienoic acid ethanolamides (EET-EAs) that bind to CB2 receptors^31,34,35^.

Herein, we evaluated whether epoxides of eVDs are more effective than parent eVDs at targeting cannabinoid receptors and TRPV1, and whether they are anti-inflammatory. We report a class of dual-functional epoxides of NADA and NA5HT (epoNADA and epoNA5HT, respectively) that reciprocally regulate both cannabinoid receptors (CB1 and CB2) and TRPV1 (Fig. 1). We synthesized NADA, NA5HT, 14′,15′-epoNADA, and 14′,15′-epoNA5HT. Using targeted lipidomics, we were able to identify the eVDs and epoxy-eVDs in porcine brain tissue, but because of the variability of the results we cannot definitively conclude their presence and levels in vivo. We then explored if NADA and NA5HT are potentially epoxidized under inflammatory conditions. We show that epoNADA and epoNA5HT are formed under inflammatory conditions by CYP epoxygenases and show that AEA potentiates the formation of epoNADA in microglial cells. We show that one potential mechanism of this potentiation may be through multiple-ligand binding to CYP-Nanodiscs using in vitro kinetics methods and molecular dynamics (MD) simulations. We further demonstrate that epoNA5HT is a potent TRPV1 antagonist suppressing intracellular Ca^2+^ response and membrane currents provoked by the TRPV1 ligand CAP in the primary afferent neurons. Altogether, we show that epoNADA and epoNA5HT act as dual CB1/2 and TRPV1 ligands and exhibit anti-inflammatory activity. These molecules are potential candidates for the development of pain therapeutics.

## Results

### Biosynthesis of epoxy-endovanilloids (eVDs)

**Fig. 2:**
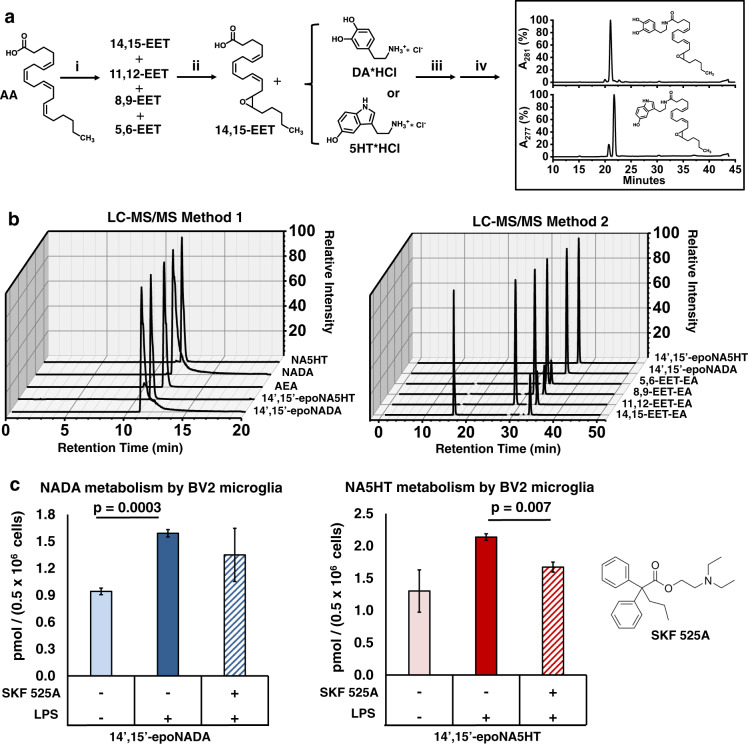
Targeted lipidomics for measuring eVDs and epoxy-eVDs. **a** Authentic standards 14’,15’-epoNADA and 14’,15’-epoNA5HT were synthesized. (i) *m-*CPBA, RT, 1 h, MeCN (ii) reverse-phase HPLC purify (iii) EDC, DMAP, DIPEA; ice bath 1 h; RT 8 h; 50:50 DCM:DMF (iv) reverse-phase HPLC purify. **b** Development of LC-MS/MS method for the separation of NADA, epo-NADA, NA5HT, epo-NA5HT and AEA (Method 1) and the different regioisomers of EET-EAs and epo-NADA and epo-NA5HT (Method 2). AEA *m*/*z* 348.3 → *m*/*z* 203.2; NADA *m*/*z* 440.2 → *m*/*z* 287.1; 14’,15’-epoNADA *m*/*z* 456.3 → *m*/*z* 137.1; 14’,15’-epoNA5HT *m*/*z* 479.3 → *m*/*z* 160.1; NA5HT *m*/*z* 463.3 → *m*/*z* 287.2; EET-EA *m*/*z* 264.2 → *m*/*z* 62.0. **c** NADA and NA5HT metabolism by BV2 microglial cells under lipopolysaccharide (LPS) stimulation in the presence of 1 µM t-AUCB (sEH inhibitor) and 10 µM NADA or NA5HT. The reversible CYP epoxygenase inhibitor SKF 525A was used to demonstrate CYP-mediated metabolism. Data represents the mean ± SEM of three experiments. Statistical significance based on a two-tailed *t*-test with equal variance. All data can be found in the Source Data file.

We first determined the levels of NADA and NA5HT in porcine brain as they express similar CYP isozymes to humans. As the epoxidation of PUFAs and eCBs by CYPs occurs mainly on the terminal alkene^32,34,36^, we synthesized 14′,15′-epoNADA and 14′,15′-epoNA5HT (Fig. 2a) (Supplementary Figs. 1–5). As shown in Fig. 2b Method 1, we developed an liquid chromatography with tandem mass spectrometry (LC-MS/MS) quantitation method in the selected reaction monitoring (SRM) mode to quantify NADA, NA5HT, 14′,15′-epoNADA, 14′,15′-epoNA5HT, and AEA from brain tissue and microglial cells.

Herein, we measured 1.7 and 1.9 pmol of NADA (0.182 ± 0.081 pmol g^−1^ wet tissue) and 11.3 and 4.9 pmol of NA5HT (0.686 ± 0.006 pmol g^−1^ wet tissue) from the cerebella of two pig brains, and the values were very low in two more pig brains that were analyzed. From the hippocampus–thalamus–hypothalamus regions, we recovered 0.4 and 0.5 pmol of NADA (0.039 ± 0.009 pmol g^−1^ wet tissue) and 0.2 and 0.1 pmol of NA5HT (0.010 ± 0.003 pmo g^−1^ wet tissue) in two pig brains. The epoxy-eVDs levels were variable and often below detection limit due to the low abundance of their parent molecules. Overall, the levels of NADA and NA5HT are much less than AEA in rat and pig brains^32^ (Supplementary Note 1).

In the field of lipid metabolites, there is strong evidence that the production of lipid metabolites is localized to the site of action and that there is a rapid subsequent degradation^37^.

This leads to low plasma/tissue levels of most lipid metabolites. Hence, we studied the epoxidation of these lipid metabolites in microglial cells. Microglial cells, the innate immune cells of the brain, are activated during neuroinflammation and play important roles in pain modulation^38^. Previously, it was demonstrated that CYPs are upregulated during neuroinflammation^39^. Hence, we used BV2 microglial cells to determine the production of eVD epoxides from NADA or NA5HT under an inflammatory stimulus. We stimulated microglial cells with lipopolysaccharide (LPS), followed by the addition of NADA or NA5HT^32^. We found that within 30 min of incubation, 14′,15′-epoNADA and 14′,15′-epoNA5HT were formed under LPS stimulation. Interestingly, these molecules are also produced without LPS stimulation showing that these molecules are made spontaneously by resting microglial cells (Fig. 2c). Importantly, the production of these metabolites were partially inhibited in the presence of SKF 525A (non-specific CYP epoxygenase inhibitor) demonstrating that the eVD epoxides are produced partly by enzymatic oxidation by CYP epoxygenases^40^ (Fig. 2c). However, there are several other CYPs in the microglial cells that may be producing these epoxidized metabolites, which can explain the partial inhibition.

### Epoxygenation of eVDs in the presence of AEA

**Fig. 3:**
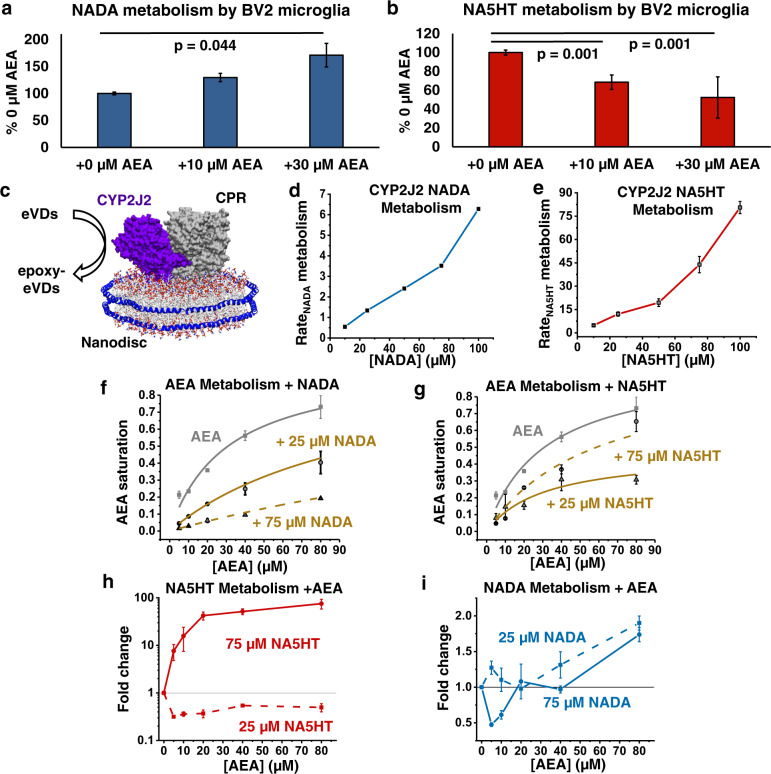
Potentiation of eVD metabolism by AEA. **a** Metabolism 10 µM NADA and **b** 10 µM NA5HT in the presence of increasing concentrations of AEA by BV2 microglia. Metabolism was conducted similarly to data in Fig. 2c. Data shown are the mean ± SEM of *n* = 6 (two sets of triplicate performed on separate days). Statistical significance is based on a one-way ANOVA with a Tukey’s post-hoc. **c** Schematic of the CYP2J2-CPR-Nanodisc. **d**, **e** Metabolism of NADA and NA5HT by CYP2J2-CPR-NDs. Rates are in pmol_epoxy-eVD_ min^−1^ nmol_CYP2J2_^−1^ and data represents the mean ± SEM of three independent experiments. **f**–**i** Co-substrate metabolism of eVDs with AEA. **f** AEA metabolism in the presence of NADA and **g** NA5HT. Rates are shown as the fraction of the *V*_max_ (135 pmol_EET-EAs_ min^−1^ nmol_CYP2J2_^−1^) for AEA metabolism (gray squares) as previously published (see text). **h** Fold change of NA5HT metabolism and **i** NADA metabolism in the presence of increasing AEA concentrations compared to the metabolism without AEA. **f**–**i** Data represents the mean ± SEM of three independent experiments. All data can be found in the Source Data file.

The endogenous levels of AEA are much higher than NADA and NA5HT. Furthermore, it has been shown that various brain CYPs such as CYP2J2 and CYP2D6 convert AEA into AEA epoxides (EET-EAs). Therefore, to understand the substrate specificity of CYPs when both substrates (AEA and NADA or NA5HT) are present, we used the activated microglial cells to study the co-metabolism of AEA with NADA or NA5HT (Fig. 3a, b). Interestingly, we observed that AEA potentiated the formation of 14′,15′-epoNADA (~2-fold) in a concentration-dependent manner (Fig. 3a). Contrariwise, AEA inhibited 14′,15′-epoNA5HT formation (~0.5-fold) (Fig. 3b). It is possible that AEA acts as a potentiator of CYP-mediated NADA metabolism and an inhibitor of CYP-mediated NA5HT metabolism, either directly or indirectly. Several ligands have shown to either act as direct potentiators or inhibitors of CYP metabolism through cooperative or allosteric binding^41,42^. To explore this possibility, we delineated the mechanism of eVD metabolism using a recombinantly expressed CYP.

Of the common CYP epoxygenases in mouse, CYP2J9 and CYP2J12 were shown to be highly expressed in mouse brain tissues while CYP2Cs showed low expression^43^. We confirmed that CYP2J9 and CYP2J12 are expressed in the BV2 microglial cells used for our studies (Supplementary Fig. 6). CYP2J9 and CYP2J12 are highly homologous to human CYP2J2. Hence, we elucidated the biochemistry of eVD metabolism in the presence and absence of AEA by human CYP2J2.

### Formation of epoxy-eVDs by human CYP2J2

CYP2J2 is highly expressed in the brain, cardiovascular, and cerebrovascular systems and is one of the major epoxygenases in these tissues^44,45^. Additionally, CYP2J2 is known to epoxidize several endocannabinoids including AEA^32,35,46^. In order to study the direct metabolism of NADA and NA5HT, we incubated CYP2J2 with each eVD and detected the metabolites using UV–Vis high-performance liquid chromatography (HPLC) and LC-MS/MS. All of the oxidized products of NADA (PD1-11) and NA5HT (PS1-7) are detailed in the Supplementary Figs. 7–18 and Supplementary Table 2. We determined that 14′,15′-epoNADA (PD5) is a product based on the fragmentation and co-elution with the synthesized standard. PS2 was confirmed to be 14′,15′-epoNA5HT. As a comparison to eVDs, we also investigated CAP as a substrate of CYP2J2, and found headgroup-oxidized products among other oxygenated products (Supplementary Figs. 19–26 and Supplementary Table 2).

### Kinetics of eVD metabolism by CYP2J2-CPR-Nanodiscs

We next incorporated CYP2J2 and CPR into Nanodiscs (ND) and proceeded to determine the kinetics of the 14′,15′-epoxide formation (Fig. 3). The metabolism of NA5HT by CYP2J2 is in a similar range as the metabolism of AEA and other lipid PUFAs,^34,36^ but the metabolism of NADA is low. Interestingly, the data demonstrate the presence of multiple binding sites, as the kinetics plots strongly deviate from a typical Michaelis-Menten model. The plots resemble the beginning of a sigmoidal curve indicating positive binding interactions. However, saturation was not achieved as the eVDs are poorly metabolized and insoluble beyond 100 µM (Fig. 3d and e). We therefore hypothesized that there are at least two binding sites. To further probe the kinetics of eVD metabolism, we measured the rate of NADPH oxidation by CYP2J2-CPR-ND in the presence of the eVDs. CPR shuttles electrons from NADPH to CYPs to facilitate the metabolism of substrates. Therefore, the rate of NADPH oxidation increases in the presence of CYP substrates. In this case, NADPH oxidation in the presence of NA5HT showed biphasic kinetics, which is unique to this substrate as described in the supplementary section (Supplementary Fig. 27).

### Relative binding affinities of NADA and NA5HT

As eCBs do not produce substantial Soret shift upon binding, we used an ebastine (EBS) competitive inhibition assay to measure the binding affinity of NADA and NA5HT^47,48^. Both NADA and NA5HT displayed competitive inhibition of EBS binding (Eq. 2), suggesting that the binding of NADA and NA5HT overlap the binding of EBS (*K*_*i*_ for NADA is 71.1 ± 20.0 μM and *K*_*i*_ for NA5HT is 49.3 ± 6.2 μM).

### Co-metabolism of AEA with NADA or NA5HT

We next determined the co-substrate kinetics of AEA with the eVDs to determine if we can explain the observed effects of AEA on BV2-mediated metabolism. We developed an LC-MS/MS method to simultaneously measure the four different regioisomers of AEA epoxides (EET-EAs) and either 14′,15′-epoNADA or 14′,15′-epoNA5HT (Fig. 2b, Method 2). We had previously determined the kinetics of AEA metabolism by CYP2J2-CPR-NDs^34^, and we repeated these experiments using two concentrations (25 and 75 μM) of either NADA or NA5HT. NADA inhibited AEA metabolism following a competitive inhibition model (Fig. 3f). A 3D global fit of the data (Supplementary Fig. 28) yields a *K*_*i*_ of 7.50 ± 0.88 μM for the inhibition of AEA by NADA, which is among the strongest endogenous inhibitors of CYP2J2 as compared to virodhamine^49^. NA5HT was a noncompetitive inhibitor (Eq. 3) at 25 μM (*K*_*i*_ = 21.4 ± 3.6 μM) and a competitive inhibitor at 75 μM (*K*_*i*_ = 86.6 ± 18.5 μM) (Fig. 3g). NADA and NA5HT also altered the regioselectivity of AEA epoxidation in a concentration-dependent manner (Supplementary Fig. 29). Interestingly, AEA showed a biphasic potentiation of NADA and NA5HT metabolism (Fig. 3h, i) when we measured the epoxy-eVD formation. Overall, the potentiation of eVD metabolism by AEA and the altered AEA regioselectivity in the presence of eVDs demonstrate that eVDs and AEA are binding to CYP2J2 at multiple sites. A further analysis of this multiple-site binding is provided in the Supplementary Note 2 and Supplementary Fig S30. Furthermore, these data support the observed crosstalk of AEA and eVDs in microglial cells (Fig. 3a, b), as at similar concentrations within each experiment AEA potentiates NADA and inhibits NA5HT.

### Molecular dynamics (MD) simulations of eVD binding to CYP2J2

**Fig. 4:**
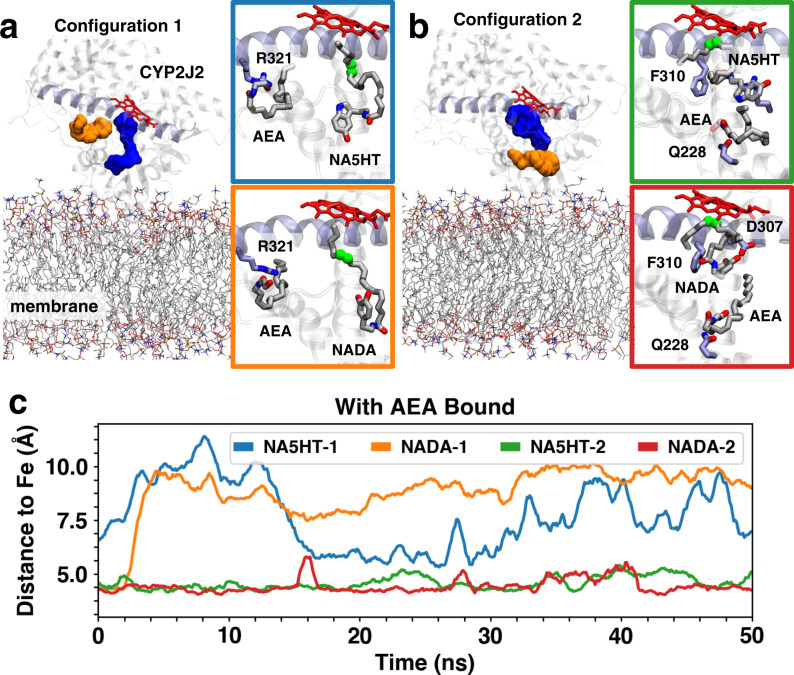
Concurrent CYP2J2 binding of AEA and NADA/NA5HT in MD simulations. Representative snapshots of two distinct configurations of AEA and NADA/NA5HT identified with molecular docking and with MD simulations: **a** peripheral binding pocket located near helix I (shown in purple cartoon) and **b** peripheral binding site near the membrane interface. In both panels, peripheral and active binding site cavity are shown as orange and blue surfaces, respectively. Lipids are shown in stick representation and CYP2J2 in cartoon representation. AEA, NADA, and NA5HT molecules are shown as sticks. Notable residues involved in AEA/NADA/NA5HT interactions are also shown as sticks. Carbons involved in epoxidation are highlighted as green spheres. **c** Time series of carbon-to-heme distances obtained from 50 ns MD simulations for the main epoxidation sites of NADA/NA5HT. Colors correspond to the subpanels shown in **a** and **b**.

In order to characterize the molecular basis of the multi-site kinetics observed with the eVDs, we performed MD simulations starting from membrane-bound structures of CYP2J2 in complex with substrates (AEA, NADA or NA5HT). Initial molecular docking was performed with AEA and either NADA or NA5HT in a stepwise manner^50^. These models allowed us to probe the binding mode of a second molecule to CYP2J2 in the presence of another molecule bound in the active site in an unbiased manner (i.e., without any assumptions about location of peripheral binding pockets). Two distinct configurations of peripheral AEA binding, with either NADA or NA5HT in the active site, were identified (Fig. 4a, b). In Configuration 1, AEA was docked in a pocket located below the I-helix, with its ethanolamine group near a residue (R321) that we have previously identified to modulate PUFA binding^36^ (Fig. 4a). In Configuration 2, AEA was located closer to the membrane interface (Fig. 4b). For the two identified AEA binding configurations, the initial orientation of NADA or NA5HT in the active site was similar, with the main epoxidation site (carbons C14 and C15) close to the heme moiety (distance <5 Å), and the headgroup (DA or 5HT) pointing away from the heme. These docking results suggested that NADA/NA5HT binding was not modulated by the same PUFA-interacting residues previously reported (T318, R321 and S493)^36^. Owing to the larger headgroups of NADA/NA5HT compared to other PUFAs (i.e., DA/5HT vs. carboxylic acid), a different binding orientation (not interacting with the PUFA triad) was necessary to fit these molecules in the active site.

The MD simulations (Fig. 4c, Supplementary Tables 3–6, and Supplementary Movies 1–4) revealed that stable NADA/NA5HT binding (i.e., with the epoxidation site distance to the heme <5 Å) was only achieved when AEA is bound in Configuration 2 (Fig. 4b). When AEA is located in the I-helix pocket (Configuration 1), NADA or NA5HT in the active site gradually move away from the heme, which results in a displacement of its epoxidation site (with the distance to heme between 7.5 and 10 Å during the simulations) (Fig. 4c). In contrast, AEA in Configuration 2 constrains the motion of NADA or NA5HT in the active site, which maintain their potentially productive orientation close to the heme (epoxidation site to heme distance <5 Å) during the simulation (Fig. 4c). In these simulations, the ethanolamine group of AEA interacted with Q228, located near the membrane interface, and remained locked in its binding site. Positioning of AEA in turn constrained the mobility of the molecule in the active site (NADA or NA5HT). NADA/NA5HT are further stabilized by hydrophobic interactions (mainly with F310) and transient electrostatic interactions (i.e., NA5HT serotonin group with D307 and E222). These observations suggest that NADA/NA5HT binding is enhanced by concurrent AEA binding to a peripheral site near the membrane interface and provide insights into the protein residues involved in this binding (e.g., Q228 for AEA and F310 for NADA/NA5HT). Overall, the MD simulations in conjunction with the kinetics data concur with the observations from the cell culture studies that AEA enhances the metabolism of NADA.

### Anti-inflammatory action of eVDs in microglial cells

**Fig. 5:**
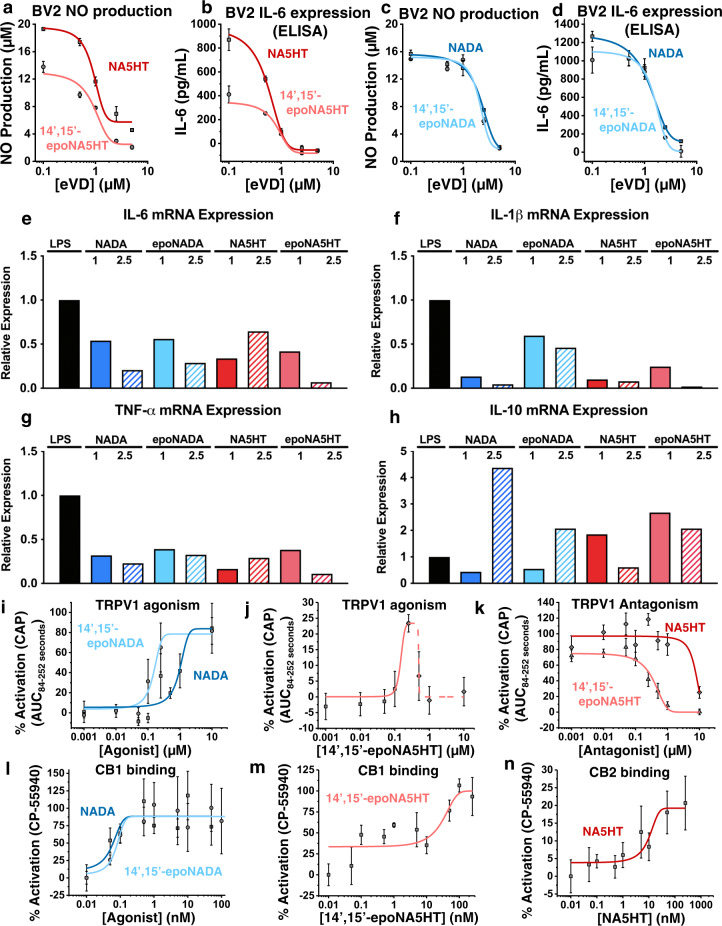
Anti-inflammatory actions of eVDs and receptor activation. **a**–**d** Anti-inflammatory effects of eVDs and epoxy-eVDs were determined by preincubating eVDs and epoxy-eVDs with BV2 cells for 4 h prior to stimulation by 25 ng mL^−1^ LPS. Data represents the mean ± SEM of *n* = 3 independent experiments. **a** Dose-dependent inhibition of nitric oxide (NO) production by NA5HT and 14,15-epoNA5HT as determined by Griess Assay. **b** Dose-dependent reduction of IL-6 protein expression (ELISA) by NA5HT and 14,15-epoNA5HT. **c** Dose-dependent inhibition of NO production and (**d**) IL-6 protein expression by NADA and 14,15-epoNADA. **e**–**h** Anti-inflammation experiments were repeated to measure pro-inflammatory *Il-6*, *Il-1β*, and *Tnf-α* and anti-inflammatory *Il-10* mRNA expression in the presence of LPS. Data represents the average of three technical replicates. Data is reported as the mean relative expression. **i**–**k** eVD activation to TRPV1-transfected HEK cells was determined using a Fura 2-AM Ca^2+^-influx assay. The *B*_*max*_ of capsaicin (CAP) activation is defined as 100%. Data represents the mean ± SEM of *n* = 6 (two sets of triplicate experiments on separate days). **k** Antagonism was determined by preincubating cells with antagonist prior to stimulating with 250 nM CAP. **l**–**n** eVD activation of CB1 and CB2 was determined by the PRESTO-Tango assay. *B*_max_ of CP-55940 is defined as 100%. Data represents the mean ± SEM of *n* = 6 (two sets of triplicate experiments on separate days). All data can be found in the Source Data file.

We further proceeded to characterize their pharmacology. Previous studies have demonstrated the anti-inflammatory actions of eVDs^51–53^; thus, we hypothesize that epoxy-eVDs would also be anti-inflammatory. Microglial cells are strongly activated after injury and release pro-inflammatory cytokines such as IL-6, IL-1β, and TNF-α. Therefore, there is a significant interest in discovering lipid-based molecules that decrease microglial activation. To investigate the actions of eVDs and epoxy-eVDs, we measured the levels of pro-inflammatory nitric oxide (NO), IL-6, IL-1β, and TNF-α in lipopolysaccharide- (LPS)-stimulated BV2 cells. All eVDs and epoxy-eVDs dose-dependently reduced NO and IL-6 production (Fig. 5a–d) and the IC_50_ values are tabulated in Table 1. Together, these data demonstrate that the eVDs and the epoxy-eVDs are anti-inflammatory mediators. As determined by MTT and BrdU assays, these compounds were not toxic at their effective concentrations, though NA5HT and 14′,15′-epoNA5HT increased cell viability in the presence of LPS, suggesting they may be pro-proliferative, which was confirmed using a BrdU assay (Supplementary Fig. 31, 32).

**Table 1:** Anti-inflammatory marker inhibition and receptor activation parameters of eVDs and epoxy-eVDs.

	NO production IC_50_ (μM)	IL-6 expression IC_50_ (μM)	TRPV1 % activation^a^	TRPV1 EC_50_ (IC_50_)^b^	CB1 % activation^c^	CB1 EC_50_^b^	CB2 % activation^c^	CB2 EC_50_^b^
NADA	0.909 ± 0.076	0.507 ± 0.065	83.8 ± 13.6	962 ± 249	83.9 ± 9.2	0.045 ± 0.01	---	DNB^d^
14’,15’-epoNADA	0.888 ± 0.135	0.807 ± 0.440	79.7 ± 9.8	156 ± 36	92.3 ± 11.2	0.080 ± 0.03	---	DNB^d^
NA5HT	2.14 ± 0.06	1.06 ± 0.49	---	(7,650 ± 1,300)	---	DNB^d^	19.2 ± 3.4	8.80 ± 5.57
14’,15’-epoNA5HT	2.07 ± 0.18	1.56 ± 0.22	23.6 ± 4.4 ---	120 ± 27 (254 ± 38)	99.9 ± 13.1	16.5 ± 11.7	---	DNB^d^

^a^Percent activation compared to the *B*_maxc_ of CAP.

^b^nM.

^c^Percent activation compared to the *B*_maxc_ of CP-55940.

^d^DNB: does not activate. Compounds upto 1 μM in concentration do not agonize the receptor and do not antagonize 50 nM of CP-55940.

We also measured the mRNA expression of *Il-6*, *Il -10, Il* -*1β*, and *Tnf-α* to determine the inflammatory phenotype. Whereas IL-6, IL -1β, and TNF-α are pro-inflammatory, IL-10 is an anti-inflammatory cytokine that facilitates the resolution of inflammation. Concentrations around the IC_50_ of the IL-6 and NO experiments (1 and 2.5 µM of each compound) were tested and compared to cells with LPS only. All the eVDs and epoxy-eVDs downregulated pro-inflammatory *Il-6, Il-1β*, and *Tnf-α* with 14′,15′-epo-NA5HT being the most effective overall (Fig. 5e–g). NADA and 14′,15′-epoNADA concomitantly upregulated *Il-10* (Fig. 5h).

Most eCBs and eVDs mediate anti-inflammation and anti-pain poly-pharmacologically through CB1, CB2, and TRPV1 receptors. We determined that the mRNA of *Cnr1* (CB1 gene), *Cnr2* (CB2 gene), and *Trpv1* are expressed in the BV2 cells (Supplementary Fig. 33). Since these receptors are known targets of eVDs and mediate inflammation and pain, we proceeded to measure the activation of CB1, CB2, and TRPV1 by epoxy-eVDs.

### Activation of TRPV1 by eVDs and epoxy-eVDs

Previous studies have shown that NADA mediates anti-inflammation via TRPV1^51^. As TRPV1 is a non-selective cation channel, activation was determined by measuring the relative Ca^2+^ influx (Supplementary Fig. 34 and Table 1). We determined that NADA and 14′,15′-epoNADA are full agonists, with ~80% activation compared to CAP (Fig. 5i). The EC_50_ of 14′,15′-epoNADA is about 6-fold tighter than NADA. 14′,15′-epoNADA also promoted a broader duration of Ca^2+^ influx opening compared to NADA (Supplementary Fig. 34). The TRPV1-selective antagonist AMG-9810 was able to fully antagonize the signal from CAP, the eVDs, and the epoxy-eVDs, confirming the Ca^2+^ influx is TRPV1-mediated (Supplementary Fig. 35b, c).

On the contrary, 14′15′-epoNA5HT was found to be a partial agonist of TRPV1 up to 250 nM, with a 24% activation compared to CAP (Fig. 5j). This signal was antagonized by AMG-9810, demonstrating it is TRPV1-mediated (Supplementary Fig. 35c). Concentrations above 250 nM, however, resulted in a reduction of the signal, signifying that 14′,15′-epoNA5HT is an antagonist of TRPV1 at higher concentrations (Fig. 5j). Previously, NA5HT had been shown to be an antagonist of TRPV1^27^; therefore, we measured the antagonism of TRPV1 by 14′15′-epoNA5HT and compared it to NA5HT. 14′15′-epoNA5HT functioned as an antagonist of CAP at all concentrations, with an IC_50_ of 250 ± 38 nM (Fig. 5k). Of note, this IC_50_ correlates to the concentration at which the self-antagonism was observed for 14′,15′-epoNA5HT (Fig. 5j). From Table 1, we see that 14′15′-epoNA5HT is a 30-fold stronger antagonist of TRPV1 than NA5HT.

### Activation of CB1 and CB2 by eVDs and epoxy-eVDs

We next determined the activation of CB1 and CB2 by eVDs and the epoxy-eVDs using the PRESTO-Tango assay^32^. Herein, NADA, 14′,15′-epoNADA, and 14′,15′-epoNA5HT, but not NA5HT, demonstrated full-agonist activation of CB1 (~100% activation as compared to CP55940) (Fig. 5l, m, Table 1). Of these, NADA activates CB1 2-fold more potently as compared to 14′,15′-epoNADA. 14′,15′-epoNA5HT activated CB1 with an EC_50_ of 16.5 ± 11.7 nM. Only NA5HT activated CB2. The measured EC_50_ was 8.80 ± 5.57 nM with 19.2% activation compared to CP55940, which makes it a partial agonist.

We further tested agonism and antagonism of NA5HT, NADA, 14′,15′-epoNADA, and 14′,15′-epoNA5HT on CB1 and CB2 receptors. None of these antagonized 50 nM CP55940 activation of these receptors (Supplementary Fig. 36c). Therefore, NA5HT does not act on CB1, and NADA, 14′,15′-epoNADA, and 14′,15′-epoNA5HT do not act on CB2. This interesting dichotomy could be exploited to design pain therapeutics that specifically target one receptor. Overall, our data shows that 14′,15′-epoNA5HT is anti-inflammatory, is an agonist of CB1, and is an antagonist of TRPV1, thereby making it the most efficacious of the eVDs for the development of pain therapeutics.

### Inhibition of TRPV1 responses in primary DRG neurons

**Fig. 6:**
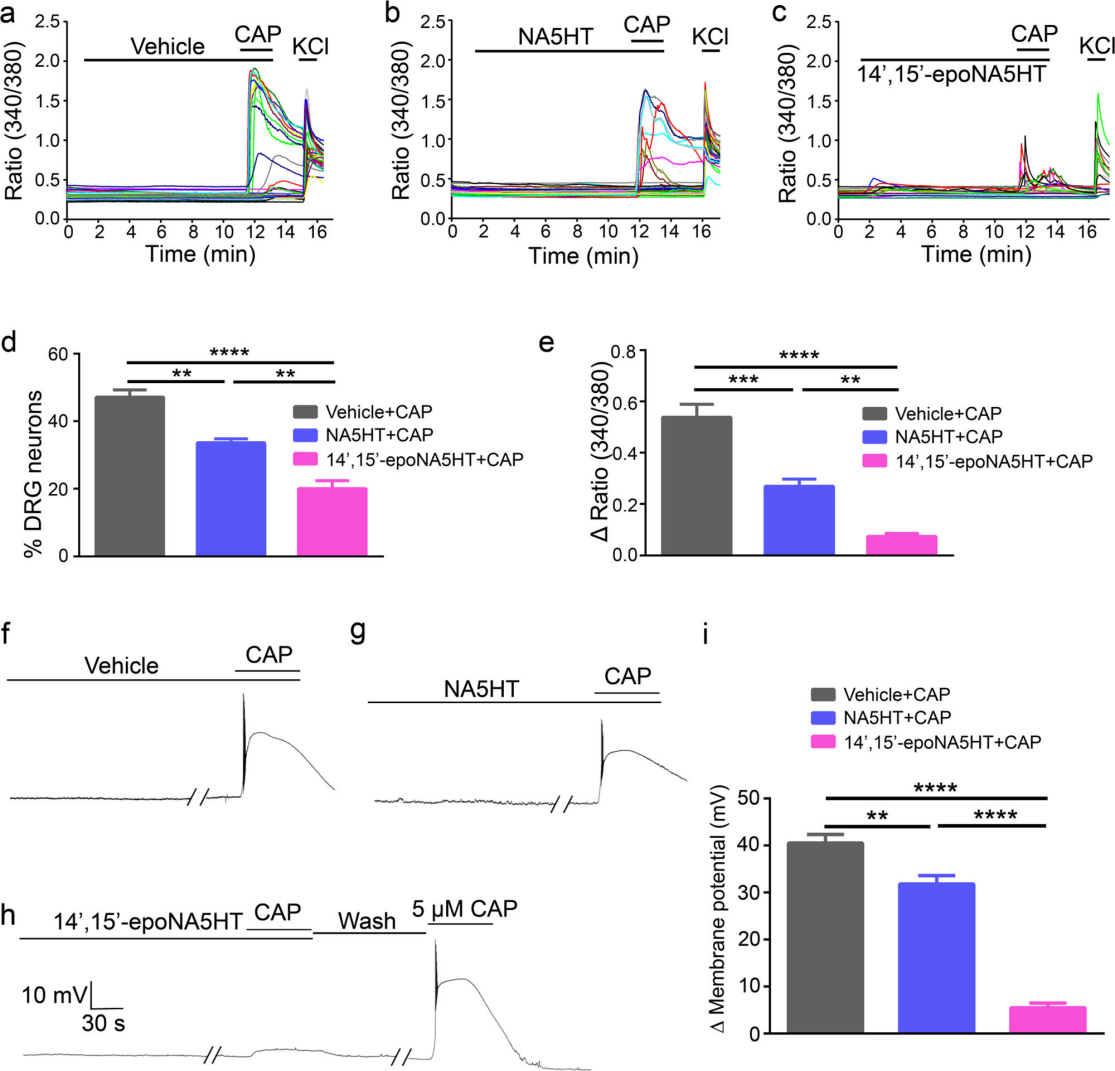
Inhibition of CAP-induced response in cultured mouse DRG neurons. **a**–**c** Ratiometric Ca^2+^ imaging of cultured wild-type mouse DRG neurons. Each trace corresponds to fluorescence in a single neuron. CAP elicited large [Ca^2+^]_i_ responses in cultured DRG neurons (**a**), which was significantly inhibited by pretreatment of NA5HT (**b**) and 14’,15’-epoNA5HT (**c**) for 10 min, respectively. **d** Percentage of DRG neurons responding to CAP with or without pretreatment of NA5HT or 14’,15’-epoNA5HT. (*n* = 5 from 4 mice; error represents SEM). *P*-values: 0.0012 (**) NA5HT+CAP vs. Vehicle+CAP, 0.0011 (**) NA5HT+CAP versus 14’, 15’-epoNA5HT+CAP, and < 0.0001 (****). **e** Quantification of CAP-induced [Ca^2+^]_i_ response with or without pretreatment of NA5HT or 14’,15’-epoNA5HT. (*n* = 5 from 4 mice; error represents SEM). *P*-values: 0.0035 (**), 0.0003 (***), and < 0.0001 (****). (**f**) Representative traces showing CAP-induced a robust membrane depolarization and action potential firing in cultured DRG neurons, which was significantly inhibited by pretreatment of NA5HT (**g**) and 14’,15’-epoNA5HT (**h**) for 10 min, respectively. **i** Summarized data showing that CAP-produced membrane depolarization and action potential firing in DRG neurons with or without pretreatment of NA5HT or 14’,15’-epoNA5HT. (*n* = 5 from three mice; error represents SEM). Drug concentration: CAP, 250 nM; NA5HT, 1 μM; 14’,15’-epoNA5HT, 1 μM; and KCl, 100 mM. *P*-values: 0.0057 (**), and <0.0001 (****). (one-way ANOVA followed by a Tukey’s post-hoc analysis). All data can be found in the Source Data file.

To further determine if NA5HT and 14′,15′-epoNA5HT could inhibit native TRPV1 expressed in mouse dorsal root ganglia (DRG) neurons, we compared CAP-evoked intracellular free Ca^2+^ ([Ca^2+^]_i_) response in cultured mouse DRG neurons with and without pretreatment of NA5HT and 14′,15′-epoNA5HT using live-cell Ca^2+^ imaging. Bath application of CAP at 250 nM produced a robust [Ca^2+^]_i_ increase in 47.12% ± 2.18% of DRG neurons (Fig. 6a, d). However, after pretreatment of 1 μM NA5HT or 1 μM 14′,15′-epoNA5HT for 10 min, 250 nM CAP could only activate 33.64% ± 1.16% and 20.01% ± 2.39% of DRG neurons, respectively (Fig. 6b–d). Consistent with the reduced number of DRG neuron activated by CAP after pretreatment of NA5HT and 14′,15′-epoNA5HT, the amplitude of CAP-induced [Ca^2+^]_i_ increase was also significantly diminished after pretreatment of NA5HT and 14′,15′-epoNA5HT (Fig. 6b, c, e). We also examined the inhibitory effect of NA5HT and 14′,15′-epoNA5HT on CAP-induced excitation of DRG neurons by using current-clamp recording. Consistent with Ca^2+^ imaging results, CAP evoked a large membrane potential depolarization and robust action potential firing, which was also significantly inhibited by pretreatment with NA5HT or 14′,15′-epoNA5HT for 10 min (Fig. 6f–i). Of note, inhibition of TRPV1-mediated [Ca^2+^]_i_ increase membrane potential depolarization by 14′,15′-epoNA5HT was significantly stronger than that produced by NA5HT (Fig. 6i), which is consistent with results in TRPV1-expressing HEK293 cells. Together, these results suggest that both NA5HT and epoNA5HT are potent TRPV1 antagonists suppressing TRPV1 function in both heterologous cells and native DRG neurons.

## Discussion

There is evidence that there is a synergism between the eCB and the opioid system that reduces the need for high doses of opioids^7^. Hence, it is important to understand the function of eCBs and their metabolites as endogenous and exogenous ligands of the receptors that are involved in pain response modulation. It has been previously shown that the activation of CB1 and CB2 is associated with anti-nociceptive and anti-inflammatory actions^10,54^. Regulation of TRPV1 can also modulate pain by mostly antagonizing TRPV1. While there are ample examples of drugs that target cannabinoid receptors or TRPV1, there is dearth of molecules that are rheostat regulators (varying strength of agonism or antagonism) of both cannabinoid receptors and TRPV1. The primary difference between many drugs and endogenous lipids is that the former are usually structurally rigid and target either CB receptors or TRPV1 whereas the latter are functionally plastic and capable of activating both receptors. NADA and NA5HT are AA derivatives of neurotransmitters. NADA is an agonist of both CB1 and TRPV1^26,55^. NA5HT is an antagonist of TRPV1. PUFAs, through parallel pathways, are converted into epoxide mediators that have been shown to reduce pain^30^. Overall, there is strong evidence for pain modulation by eCB and PUFA epoxides through multiple receptors.

**Fig. 7:**
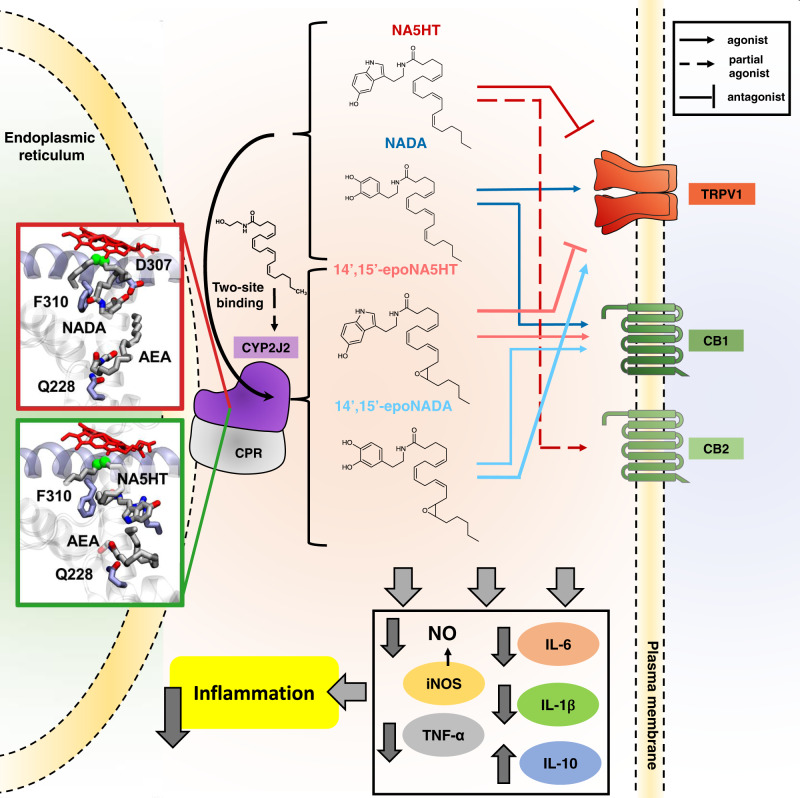
Summary of the eVD metabolism pathway and pharmacology. The eVDs bind in a two-site model to CYP2J2 and other epoxygenases and are metabolized to form epoxy-eVDs. AEA potentiates the metabolism of eVDs as revealed by BV2 metabolism assays, in vitro CYP2J2 kinetics, and molecular dynamics simulations. 14’,15’-epoNADA is a better TRPV1 agonist and slightly weaker CB1 agonist compared to NADA. 14’,15’-epoNA5HT is a better TRPV1 antagonist compared to NA5HT, and is a CB1 full agonists as opposed to NA5HT, which is a partial CB2 agonist. Overall, the eVDs and epoxy-eVDs potently downregulate pro-inflammatory NO production and *Il-6*, *Il-1ß*, and *Tnf-α* expression while increasing *Il-10* expression, thereby demonstrating that they are potently anti-inflammatory.

Using a combined biophysical and cell-based approach, we report the pharmacological characterization of NADA and NA5HT epoxides that are produced by the CYP epoxygenase pathway in microglial cells. These molecules are anti-inflammatory and function through the eCB-TRPV1 axis. These results can potentially inspire new therapeutics that effectively target this axis. The overall findings of this work are outlined in Fig. 7 and discussed below.

The biosynthesis of the epoxy-eVDs is facilitated by CYP epoxygenases as the inhibiton of CYP enzymes in microglial cells reduce the levels of these metabolites. In the pig brain, we were able to detect the parent compounds NADA and NA5HT, whose levels were much lower and variable than that of AEA. The epoNADA and epoNA5HT were spontaneously formed by microglia cells in the presence and absence of inflammatory stimulus. As the levels of AEA were high and eVDs were low, we tested the effect of eVD metabolism by CYPs in the presence of AEA. Interestingly, the metabolism of NADA is potentiated by AEA in the microglial cells. This was an interesting observation as there are very few examples where the binding of one ligand at the enzyme active site potentiates the metabolism of another ligand. However, such complicated substrate interactions are common for CYPs with a large active sites such as CYP3A4^41,42,56^. We demonstrate that AEA is a complicated effector of eVD metabolism in CYP2J2, which potentially explains the observed potentiation of NADA metabolism in BV2 cells. However, AEA may also be inhibiting unknown enzymes that degrade NADA or 14′,15′-epoNADA.

We then proceeded to investigate the the interactions of AEA and the eVDs at the enzymological level to gain better molecular insight into the metabolism. CYP2J9, CYP2J12, and CYP2J2 were all shown to be highly expressed in mouse and human brains^43,44,57–59^. Additionally, CYPs such as CYP2J6 are expressed in DRGs and is involved in the epoxidation of lipids that act on TRPV1 when paclitaxel is administered. In addition, CYP2J4 is found in TRPV1-positive rat trigeminal ganglia, which are also involved in pain-temperature sensing pathways^60^. Comparing the in cellulo metabolism data to the in vitro kinetic measurements with CYP2J2-NDs reveals a complex network of substrate-substrate interactions. Previously, we determined that PUFAs bind CYP2J2 at two main sites: the substrate access channel and the PUFA binding pocket^36^. AEA binds to CYP2J2 with PUFA-like properties: that is, by binding similar sites in the substrate access channel and PUFA binding pocket. However, NADA and NA5HT do not bind the PUFA binding pocket and have unstable binding to the substrate access channel. Therefore, AEA can simultaneously be accommodated in binding to CYP2J2. The MD simulations support that up to three overlapping binding sites are possible, which can help to explain the observed complex AEA and eVD interactions, such as the regioselectivity change in the AEA metabolites (Supplementary Fig. 29) and the potentiation of eVD metabolism (Figs. 3 and 4). We have previously observed complex inhibition and regioselectivity changes for endogenous substrates of CYP2J2^34,49,50^; however, this is the first report of a potentiation of CYP2J2 metabolism. Overall, the finding implies that the co-substrate AEA potentiates the metabolism of NADA and NA5HT by CYP2J2 in vitro. Therefore, this study provides another therapeutic route where drugs can potentiate CYP2J2’s epoxidation of endogenous lipids.

One common observation is that the oxidized eVD metabolites exhibit different pharmacology compared to the parent molecules. CYP2U1 metabolizes NA5HT to a 2-oxo derivative that is a weaker FAAH inhibitor compared to NA5HT^24^. NADA was shown to be hydroxylated at the ω and ω-1 positions by rat liver microsomes, which were weaker TRPV1 agonists compared to NADA^26^. Therefore, the 2-oxo-NA5HT and NADA-OH metabolites may represent a degradation pathway of NADA and NA5HT. However, we show that the epoxy-eVDs are potently anti-inflammatory molecules in activated microglial cells.

A key observation is that the epoxidation of eVDs increases their activity on TRPV1. 14′,15′-epoNADA is a 6.5-fold stronger agonist than NADA and 14′,15′-epoNA5HT is a 30-fold stronger antagonist than NA5HT at TRPV1, while also showing partial agonism at lower concentrations. Moreover, both NA5HT and 14′,15′-epoNA5HT suppressed TRPV1-mediated [Ca^2+^]_i_ response and membrane potential depolarization in mouse DRG neurons with 14′,15′-epoNA5HT exerting a significantly stronger inhibition of TRPV1-mediated responses than that produced by NA5HT. Based on the cryo-EM structure of TRPV1, it has been proposed that agonists binding at the CAP-binding pocket facilitate channel opening by promoting the lateral movement of the S4-S5 linker^61^. The epoxide could be forming some interactions with H-bond donating groups such as Tyr or Asp residues that populate this linker, which facilitates the channel opening. Since TRPV1 exists as a tetramer, the binding of 14′,15′-epoNA5HT to different sites and perhaps different monomers may help to explain its dual agonism/antagonism. However, since the activation of TRPV1 is self-antagonized at concentrations greater than 250 nM (around the IC_50_), the 14′,15′-epoNA5HT functions overall as an antagonist.

The epoxidation of NA5HT changes it from being a partial CB2 agonist to a full CB1 agonist. This is intriguing given that 14′,15′-epoNA5HT and NA5HT differ only by an epoxide. Contrariwise, the epoxidation of NADA does not greatly alter their potency towards CB1 receptor activation. Previously it was shown that the epoxidation of omega-6 and omega-3 eCBs have preferential activation towards CB2^32,46^. Herein, we show that eVD epoxides target CB1 receptors. While NADA and 14′,15′-epoNADA are TRPV1 and CB1 agonists, NA5HT and 14′,15′-epoNA5HT are TRPV1 antagonists. The complex functional crosstalk of CB1 and TRPV1 is still being elucidated,^47,62^ but the studies suggest that the plasticity exhibited by endogenous lipids indirectly contributes to this crosstalk.

Overall, the eVDs and epoxy-eVDs lower pro-inflammatory cytokines while increasing anti-inflammatory IL-10. They also potently activate cannabinoid receptors and are potent ligands of TRPV1. The epoxidation of eVDs increases their potency at TRPV1 and alters their pharmacology at cannabinoid receptors. In particular, 14′,15′-epoNA5HT is the most effective epoxy-eVD at reducing pro-inflammatory markers. 14′,15′-epoNA5HT is also a potent antagonist of TRPV1 expressed in either HEK293 cells or native DRG neurons and a potent full agonist of CB1. Lastly, the formation of epoxy-eVDs by CYPs is potentiated by the co-substrate AEA, which is also metabolized by CYPs to form EET-EA that are potent CB2 ligands. Hence, inflammation and related pain response is accompanied by a storm of epoxy-eVDs and epoxy-eCBs that are multi-faceted endogenous molecules capable of influencing the activity of CB1, CB2 and TRPV1 receptors. The discovery of these molecules will serve as templates for new multi-target therapeutic drugs that will prove useful for the treatment of inflammatory pain, as well as of other conditions in which these receptors are targeted in other clinical studies.

## Methods

Materials and methods concerning RT-qPCR, IL-6 ELISA kit-based detection, Griess Assay to detect nitric oxide, cell viability assay using MTT, cell proliferation assay using BrdU, protein expression and purification methods, nanodisc assembly, in vitro lipid metabolism, and full-scan mass spectrometry analysis of the metabolites can be found in the supplementary information.

### Synthesis of NADA and NA5HT

Dopamine hydrochloride (24.9 mg, 0.131 mmol, 2.0 equiv.) or serotonin hydrochloride (27.9 mg, 0.131 mmol, 2.0 equiv.) was added to N,N-diisopropylethylamine (DIPEA, 27.4 μL, 0.145 mmol, 2.2 equiv.) in an anhydrous solution of DMF/CH_2_Cl_2_ (1/1 v/v, 25 mL) under an argon atmosphere. This mixture was cooled in an ice-water bath and then AA (21.7 μL, 0.066 mmol, 1.0 equiv.), 1-ethyl-3-(3-dimethylaminopropyl)-carbodiimide (EDC, 63 mg, 0.33 mmol, 5.0 equiv.), and 4-dimethylaminopyridine (DMAP, 2 mg, 0.0099 mmol, 0.15 equiv.) were added. After 1 h, the reaction was warmed to room temperature and allowed to incubate at the same temperature for 8 h. Afterwards, toluene was added, and the reaction was concentrated under reduced pressure. The product was extracted 3× from water with CH_2_Cl_2_ and then washed 1× with brine. The organic layer was dried over sodium sulfate and concentrated under reduced pressure to yield a yellow-brown oil. NADA [(5Z,8Z,11Z,14Z)-N-(3,4-dihydroxyphenethyl)icosa-5,8,11,14-tetraenamide] and NA5HT [(5Z,8Z,11Z,14Z)-N-(2-(5-hydroxy-1H-indol-3-yl)ethyl)icosa-5,8,11,14-tetraenamide] were purified using HPLC Method 2 below. Products were dried under reduced pressure, stored in ethanol, and quantified via HPLC as stated below. Yields were 13.0 mg NADA (45%) and 12.8 mg NA5HT (45%). **[NADA]** ^1^H-NMR (400 MHz, CDCl_3_) *δ* 6.96 (s, 1H); 6.80 (d, *J* = 8.0 Hz, 1H); 6.74 (d, *J* = 2.0 Hz, 1H); 6.58 (dd, *J* = 8.1, 2.0 Hz, 1H); 5.61 (s, 1H); 5.54 (d, *J* = 6.5 Hz, 1H); 5.44–5.27 (m, 8H); 4.12 (q, J = 7.1 Hz, 1H) (ethyl acetate); 3.48 (q, *J* = 6.7 Hz, 2H); 2.88–2.75 (m, 6H) (part ethyl acetate); 2.70 (t, *J* = 7.1 Hz, 2H); 2.19–2.12 (m, 2H); 2.12–1.99 (m, 6H); 1.68 (p, *J* = 7.4 Hz, 2H); 1.41–1.19 (m, 8H) (part ethyl acetate); 0.88 (t, *J* = 6.7 Hz, 3H). HRMS (*m*/*z*): [M + H^+^] cal’d 440.3165, observed 440.3161 (−0.9 ppm); C_28_H_42_NO_3_. **[NA5HT]**. ^1^H-NMR (500 MHz, CDCl_3_) *δ* 7.90 (s, 1H); 7.04–6.98 (m, 2H); 6.82–6.76 (m, 1H); 5.53 (s, 1H); 5.44–5.29 (m, 8H); 3.57 (q, *J* = 6.6 Hz, 2H); 2.90 (t, *J* = 6.8 Hz, 2H); 2.80 (dt, *J* = 18.2, 5.9 Hz, 6H); 2.08 (ddt, *J* = 31.1, 14.6, 7.4 Hz, 6H); 1.73–1.63 (m, 2H); 1.40–1.16 (m, 7H) (part ethanol); 0.88 (t, *J* = 6.7 Hz, 3H). HRMS (*m*/*z*): [M + H^+^] cal’d. 463.3325, observed 463.3322 (−0.6 ppm); C_30_H_43_N_2_O_2._

### Synthesis of 14′,15′-epoNADA and 14′,15′-epoNA5HT

Synthesis of EETs was performed using *m-*chloroperoxybenzoic acid (*m*-CPBA) according to our previously established protocol^32^. AA (54.3 µL, 0.165 mmol, 1.0 equiv.) was combined with *m*CPBA (, 0.33 mmol, 2.0 equiv.) in 2 mL dichloromethane (DCM) and allowed to react at room temperature for 1 h. The reaction was terminated with an equivolume solution of 10% NaHCO_3_ (aq) and the aqueous layer was re-extracted thrice with DCM. The organic layers were combined and resuspended with acetonitrile for reversed-phase HPLC separation [Mobile Phase A 95:4.9:0.1 H_2_O:acetonitrile:acetic acid; Mobile Phase B 95:4.9:0.1 acetonitrile:H_2_O:acetic acid; linear gradient, 50% Solvent B to 100% Solvent B over 1 h using a SunFire^TM^ Prep C_18_ OBD^TM^ (see below)] of the terminal epoxide (14,15-EET) from the other regioisomers. Yield: 4–8 mg of 14,15-EET (7.6-15% yield). 14,15-epoNADA [(5Z,8Z,11Z)-N-(3,4-dihydroxyphenethyl)-13-(3-pentyloxiran-2-yl)trideca-5,8,11-trienamide] and 14,15-epoNA5HT [(5Z,8Z,11Z)-N-(2-(5-hydroxy-1H-indol-3-yl)ethyl)-13-(3-pentyloxiran-2-yl)trideca-5,8,11-trienamide] were synthesized by coupling DA and 5HT to 14,15-EET using the above coupling method. Yield from 14,15-EET: 3.6 mg (65%) 14′,15′- epoNADA, 4.7 mg (40%) 14′,15′-epoNA5HT. **[14′,15′-epoNADA]** ^1^H-NMR (400 MHz, CDCl_3_) *δ* 6.80 (d, *J* = 8.1 Hz, 1H); 6.72 (s, 1H); 6.58 (d, *J* = 7.9 Hz, 1H); 5.81 (s, 1H); 5.65–5.25 (m, 6H); 3.47 (q, *J* = 6.5 Hz, 2H); 3.01 (d, *J* = 5.4 Hz, 1H); 2.80 (d, *J* = 20.6 Hz, 4H); 2.69 (t, *J* = 6.8 Hz, 2H); 2.45 (m, *J* = 7.3 Hz, 1H); 2.24 (m, *J* = 6.8 Hz 1H); 2.17–1.92 (m, 4H); 1.79–1.62 (m, 2H); 1.34 (q, *J* = 5.1, 3.7 Hz, 4H); 0.98–0.77 (m, 3H). HRMS (*m*/*z*): [M + H^+^] cal’d. 456.3114, observed 456.3104 (−2.2 ppm); C_28_H_42_NO_4._ **[14′,15′-epoNA5HT]** ^1^H-NMR (500 MHz, CDCl_3_) *δ* 7.97 (s, 1H); 7.13–6.89 (m, 2H); 6.79 (dd, *J* = 8.7, 2.5 Hz, 1H); 5.60 (s, 1H); 5.55–5.29 (m, 7H); 5.23 (s, 1H); 3.56 (p, *J* = 8.1, 7.3 Hz, 2H); 2.96 (d, *J* = 7.2 Hz, 2H); 2.89 (q, *J* = 7.0 Hz, 2H); 2.79 (dt, *J* = 23.0, 6.3 Hz, 4H); 2.42 (d, *J* = 6.7 Hz, 1H); 2.21 (dt, *J* = 13.8, 6.8 Hz, 1H); 2.09 (dq, *J* = 20.9, 7.4 Hz, 4H); 1.68 (p, *J* = 7.6 Hz, 2H); 1.47–1.19 (m, 6H), 0.89 (d, *J* = 7.1 Hz, 3H). HRMS (*m*/*z*): [M + H^+^] cal’d. 479.3274, observed 479.3269 (−1.0 ppm); C_30_H_43_N_2_O_3._

### Extraction of NADA and NA5HT from porcine brain regions

Porcine brains were obtained from freshly slaughtered swine from the Meat Science Laboratory at the University of Illinois at Urbana-Champaign (Ryan Dilger Lab). The swine are from a commercial swine line for the swine industry (1050 Cambro line) and were fed a standard diet. The brains were dissected into regions (cerebellum; central core comprising the hippocampus, hypothalamus, and thalamus; and the cerebrum containing the cerebral cortex) and diced immediately after removal from the pig. The tissue was then flash frozen and stored at −80 °C until used. For extraction, the tissue was homogenized in 5 volumes of ice-cold methanol (MeOH) containing 0.03 mm of the soluble epoxide hydrolase inhibitor 4-[[trans-4-[[(tricyclo[3.3.1.13,7]dec-1-ylamino)carbonyl]amino]cyclohexyl]oxy]-benzoic acid (t-AUCB) and 1 mm of FAAH inhibitor phenylmethylsulfonyl fluoride (PMSF). Debris was filtered using a filter column. The extract was then dried under reduced pressure and resuspended in 50% MeOH. The extracts were then loaded onto 1-gram Bond Elut C-18 cartridges (Varian, Harbor City, CA) (one column per gram of tissue), preconditioned with 25% MeOH. Cartridges were washed with 4 mL of 10% MeOH and eluted with 4 mL of 100% acetonitrile (MeCN). The eluates were dried under reduced pressure in amber vials and stored under Ar_(*g*)_ at −80 °C until resuspended in 150 μL of 180-proof ethanol for LC-MS/MS analysis (the day after). Samples of tissue were spiked with 1 µg of NADA or NA5HT standards to test the recovery of this method, and we were able to recover 40% of the material.

### HPLC analysis of NADA, NA5HT, and metabolites

Compounds were analyzed and separated via HPLC consisting of an Alliance 2695 analytical separation module (Waters, Milford, MA) and a Waters 996 photodiode diode array detector (Waters). Synthesis purification of EETs and epoxy-eVDs were separated in reverse-phase using a SunFire^TM^ Prep C_18_ OBD^TM^ 5 μm 19 × 50 mm column (Waters) and a 3.0 mL min^−1^ flow rate, and for quantification using a Phenomenex Prodigy® 5μm ODS-2, 150 × 4.60 mm column (Phenomenex, PN 00F-3300-E0, Torrance, CA) with a 1 mL min^−1^ flow rate. Mobile Phase A consisted of 95:5% H_2_O (0.1% acetic acid):MeCN and Mobile Phase B consisted of 5:95% H_2_O (0.1% acetic acid):MeCN. A full-scan method (Method 1) was developed to investigate all potential products from in vitro enzyme reactions as follows: 0–1 min, 100% A; 1–60 min, linear gradient of 100% A to 100% B; 60–65 min, 100% B. NADA and NA5HT elution times were confirmed using authentic standards (59.5 min and 60 min, respectively). A shorter method (Method 2) was developed to analyze the hydrophobic products and for synthesis purification and quantification. 0–30 min: 100% A to 100% B; 30–40 min: 100% B. All wavelengths from 190–600 nm were monitored. 14′,15′-epoNADA and 14′,15′-epoNA5HT were quantified at 281 nm and 277 nm wavelengths, respectively, using a NADA and NA5HT standard curve, respectively.

### LC-MS/MS quantitation of NADA, NA5HT, and AEA from tissue

Samples were analyzed with the 5500 QTRAP LC/MS/MS system (Sciex, Foster City, CA) in Metabolomics Lab of Roy J. Carver Biotechnology Center, University of Illinois at Urbana-Champaign. Software Analyst 1.6.2 was used for data acquisition and analysis. The 1200 series HPLC system (Agilent Technologies, Santa Clara, CA) includes a degasser, an autosampler, and a binary pump. The LC separation was performed on an Agilent SB-Aq column (4.6 × 50mm, 5μm) with mobile phase A (0.1% formic acid in water) and mobile phase B (0.1% formic acid in acetontrile). The flow rate was 0.3 mL min^−1^. The linear gradient was as follows: 0–1 min, 90%A; 8–13min, 0%A; 13.5–18min, 90%A. The autosampler was set at 10 °C. The injection volume was 10 μL. Mass spectra were acquired under positive electrospray ionization (ESI) with the ion spray voltage of 5500 V. The source temperature was 450 °C. The curtain gas, ion source gas 1, and ion source gas 2 were 32 psi, 50 psi, and 65 psi, respectively. Multiple reaction monitoring (MRM) was used for quantitation: AEA *m*/*z* 348.3 → *m*/*z* 203.2; NADA *m*/*z* 440.2 → *m*/*z* 287.1; 14′,15′-epoNADA *m*/*z* 456.3 → *m*/*z* 137.1; 14′,15′-epoNA5HT *m*/*z* 479.3 → *m*/*z* 160.1; NA5HT *m*/*z* 463.3 → *m*/*z* 287.2. Internal standard AEA-d4 was monitored at *m*/*z* 352.3 → *m*/*z* 287.2.

### Cell culture

HEK cells stably transfected with human TRPV1 (HEK-hTRPV1) were a gift from Prof. Bradshaw (University of Indiana, Bloomington), which were originally constructed by Merck Research. Cells were grown in Eagle Minimum Essential Media supplemented with l-glutamine (EMEM) (ATCC) and 10% fetal bovine serum (FBS) supplemented with penicillin (100 U mL^−1^) and streptomycin (0.1 mg mL^−1^) and were incubated at 37 °C with 5% CO_2_. Cells were sub-cultured at 80–90% confluency by trypsinization in a 1:6-1:10 ratio. HTLA cells for PRESTO-TANGO and BV2 microglia were grown according to our previously established protocol^32^. HTLA and BV2 cells were maintained in Dulbecco’s modified Eagle’s media (DMEM) with 10% FBS with penicillin (100 U mL^−1^) and streptomycin (0.1 mg mL^−1^) at 37 °C with 5% CO_2_ and sub-cultured at 80–90% confluency by trypsinization. HTLA cells were further supplemented with 2 μg mL^−1^ of puromycin and 100 μg mL^−1^ of hygromycin B.

### Isolation and short-term culture of mouse DRG neurons

Mice were killed by cervical dislocation following CO_2_ asphyxia and spinal columns were removed and placed in ice-cold HBSS. Laminectomies were performed and bilateral DRGs were dissected out. After removal of connective tissues, DRGs were digested in 1 mL of Ca^2+^/Mg^2+^-free HBSS containing 20 U of papain (Worthington, Lakewood, NJ), 0.35 mg of l-cysteine and 1 μL of saturated NaHCO_3_ and incubated at 37 °C for 10 min. The DRG suspension was centrifuged, the supernatant was removed, and 1 mL of Ca^2+^/Mg^2+^-free HBSS containing 4 mg of collagenase type II and 1.25 mg of Dispase type II (Worthington) was added and incubated at 37 °C for 15 min. After digestion, neurons were pelleted; suspended in neurobasal medium containing 1% l-glutamine, 2% B-27 supplement, 100 U mL^−1^ penicillin plus 100 μg mL^−1^ streptomycin, and 50 ng mL^−1^ nerve growth factor. The cells were plated on a 12-mm coverslip coated with poly-l-lysine (10 μg mL^−1^) and cultured under a humidified atmosphere of 5% CO_2_/95% air at 37 °C for 24 h.

### Metabolism of NADA and NA5HT by BV2 microglia

BV2 microglia were plated on 6-well plates at 5 × 10^5^ cells per well and grown to 80–90% confluency. Cell growth media was exchanged for 2 mL of serum-free DMEM and cells were then stimulated with 100 ng mL^−1^ of LPS for 12 h; control cells were without LPS stimulation. Afterwards, 1 μM of t-AUCB with or without 1 μM of the CYP inhibitor SKF 525A were added for 30 min. 10 μM of NADA or NA5HT were then added for 30 min with or without 10 μM or 30 μM AEA. Cells were scraped into media and combined with 2 mL ice-cold methanol. Cells were lysed using three consecutive 30-s on/off cycles on a water-bath sonicator. Cell debris was pelleted via centrifugation and the supernatant was purified using 100-mg Bond Elut C-18 cartridges (Varian, Harbor City, CA). Elution fractions were dried under reduced pressure, resuspended in 150 μL of 180-proof ethanol, and analyzed as stated above for tissue extractions. To account for batch-to-batch variability, data in the presence of AEA were analyzed based on a percentage to controls without AEA.

### TRPV1 binding/activation measurements

Binding of NADA, NA5HT, and epoxy-eVDs to TRPV1 was determined using an intracellular Ca^2+^ fluorescent quantification method. HEK-hTRPV1 cells were grown for 3 passages after recovery from frozen stocks before plating on Corning CellBind black, clear-bottom 96-well fluorescence plates coated with poly-L-lysine. After 24 h, media was removed, and cells were loaded with 3 μM Fura-2 AM dye (Molecular Probes) in sterile-filtered HEPES-Tyrode Buffer (HTB) (Alfa Aesar) supplemented with 0.01% Plurionic F-127 (Molecular Probes) for 20 min at room temperature. Analytes were prepared from DMSO stocks in 150 μL HTB on separate 96-well plates so that <0.1% DMSO was introduced to the cells. Dye was removed and cells were washed twice with HTB and 100 μL of HTB was added to the cells for the assay. To confirm binding to TRPV1, 0.5 μM of the TRPV1-specific antagonist AMG-9810 was added to this 100 μL of HTB prior to stimulating with agonists. Cells were then incubated at room temperature for 20 min to allow for the de-acetylation of the dye. Fluorescence readings were conducted on a SpectraMax Gemini EM (Molecular Devices, San José, Ca) plate reader using the following settings: bottom-read; channel 1—340 nm excitation, 510 nm emission; channel 2—380 nm excitation, 510 nm emission; 2-s mix before experiment; read every 14 sec; 5-min experiment. The assays were conducted at room temperature (25 °C). 100 μL of agonists were transferred in triplicate via multi-channel pipette to initiate the assay and the fluorescence intensities of both channels were measured over 5 min. The intensity from channel 1 (Ca^2+^-bound Fura-2) was divided by the intensity from channel 2 (Ca^2+^-free Fura-2) to achieve the Fluorescence Ratio $\left( {\frac{{I_{340ex/510em}}}{{I_{380ex/510em}}}} \right)$. The Fluorescence Ratio was then plotted over time. Owing to variations in the activation, the AUC of the Fluorescence Ratio from 84–252 s was used to determine activation. The average AUC of DMSO from 84–252 s was considered baseline and subtracted from each data point. The AUCs were plotted as a function of concentration and fitted to a dose-response curve (Eq. 5) using OriginPro. CAP was used as a full-agonist positive control, and the *B*_max_ of the CAP was defined as 100% activation. Antagonism experiments for NA5HT, 14′,15′-epoNA5HT, and AMG-9810 were determined by adding varying concentrations of antagonist in 100 μL of HTB 15 min prior to stimulating with 250 nM CAP.

### Animals

Male and female C57BL/6J mice were ordered from Jackson Laboratories and were used at the age of 7–8 weeks. Mice were housed in a humidity- and temperature-controlled environment under a 12-h light/12-h dark cycle with free access to water and food. The mice were randomly used and all experiments were done blind to genotypes of animals. All experimental procedures and animal care were performed in accordance with the guidelines of the National Institutes of Health and were approved by the Institutional Animal Care and Use Committee at Washington University School of Medicine.

### Live-cell Ca^2+^ imaging

Cultured DRG neurons were loaded with 4 μM Fura-2 AM (Life Technologies) in culture medium at 37 °C for at least 60 min before use. Cells were washed three times and incubated in HBSS at room temperature for 30 min. Fluorescence at 340 nm and 380 nm excitation wavelengths was recorded on an inverted Nikon Ti-E microscope equipped with 340-, 360-, and 380-nm excitation filter wheels with NIS-Elements imaging software (Nikon Instruments Inc.). Fura-2 ratios (F340/F380) reflect changes in [Ca^2+^]_i_ upon stimulation. Values were obtained from 50–100 cells in time-lapse images from each coverslip. Threshold of activation was defined as 3 SD above the average (∼20% above the baseline).

### Whole-cell Patch-clamp recording

Whole-cell patch-clamp recordings were performed using an Axon 700B amplifier (Molecular Devices, Sunnyvale, CA, USA) with Clampex 10.4 software (Molecular Devices). at room temperature (22–24 °C) on the stage of an inverted phase-contrast microscope equipped. Pipettes were pulled from borosilicate glass (BF 150-86-10; Sutter Instrument, Novato, CA, USA) with a Sutter P-1000 pipette puller, which had resistances of 2–4 megaohms when filled with pipette solution containing 140 mM KCl, 1 mM EGTA, 1 mM MgCl_2_, 5 mM MgATP and 10 mM HEPES with pH 7.3 and 320 mOsm L^−1^ osmolarity. Cells were continuously perfused with extracellular solution containing the following: 140 mM NaCl, 2 mM CaCl2, 1 mM MgCl2, 5 mM KCl, 10 mM glucose and 10 mM HEPES, pH adjusted to 7.4 with NaOH and the osmolarity was adjusted to ≈340 mOsm L^−1^ with sucrose. Data were analyzed and plotted using Clampfit 10 (Molecular Devices).

### PRESTO-TANGO binding with CB1 and CB2

Binding of eVDs to CB1 and CB2 was performed in HTLA cells using the PRESTO-TANGO assay as previously described^32^. HTLA cells, (a HEK293 cell line stably expressing a tTA-dependent luciferase reporter and a β-arrestin2-TEV fusion gene) were a gift from Brian Roth’s lab. Cells were seeded at 20,000 cells per 100 µL into a poly-l-lysine coated 96-well plate. After 24 h, cells were transfected with *cnr1* or *cnr2* plasmids (0.1 µg per well) using Calfectin (0.4 µL) in a 4:1 reagent to plasmid ratio (final volume 110 µL per well). Transfection media was replaced after 18 h with fresh serum-media and maintained for 48 h. On the day of the assay, serum-media was replaced with 100 µL media containing 1% dialyzed FBS for 4 h. Cells were then incubated with 1 μM of t-AUCB for 30 min and then compound dissolved with media containing 1% dialyzed FBS and was added in a log dose manner (final volume 200 µL per well) and incubated for 8 h. Media was then exchanged for 40 µL of 20×-diluted Bright-Glo (Promega, Madison, WI) solution and incubated in the dark for 20 min and analyzed by luminescence readings. CP-55940 was used as a full-agonist positive control for both receptors, and its *B*_max_ was defined as 100% activity. For antagonism experiments, analytes were co-administered at varying concentrations with 50 nM CP-55940.

### Quantitation of epoxy-eVDs using LC-MS/MS

Samples were analyzed with the 5500 QTRAP LC/MS/MS system (Sciex, Framingham, MA) in Metabolomics Lab of Roy J. Carver Biotechnology Center, University of Illinois at Urbana-Champaign. Software Analyst 1.6.2 was used for data acquisition and analysis. The 1200 series HPLC system (Agilent Technologies, Santa Clara, CA) includes a degasser, an autosampler, and a binary pump. The LC separation was performed on an Agilent SB-Aq (4.6 x 50mm, 5 μm) with mobile phase A (0.1% formic acid in water) and mobile phase B (0.1% formic acid in MeCN). The flow rate was 0.3 mL min^−1^. The linear gradient was as follows: 0–1 min, 90%A; 8–13 min, 0%A; 13.5–18 min, 90%A. The autosampler was set at 10 °C. The injection volume was 10 μL. Mass spectra were acquired under positive electrospray ionization (ESI) with the ion spray voltage of +5000 V. The source temperature was 450 °C. The curtain gas, ion source gas 1, and ion source gas 2 were 30, 65, and 55, respectively. Multiple reaction monitoring (MRM) was used for quantitation: 14′,15′-epoNA5HT *m*/*z* 479.3 → *m*/*z* 160.0; 14′,15′-epoNADA *m*/*z* 456.3 → *m*/*z* 137.1. Internal standard Anadamide-d4 was monitored at *m*/*z* 352.3 → *m*/*z* 287.2.

### Quantitation of EET-EAs and eVDs using LC-MS/MS

Samples were analyzed with the 5500 QTRAP LC/MS/MS system (Sciex, Framingham, MA) in Metabolomics Lab of Roy J. Carver Biotechnology Center, University of Illinois at Urbana-Champaign. Software Analyst 1.6.2 was used for data acquisition and analysis. The 1200 series HPLC system (Agilent Technologies, Santa Clara, CA) includes a degasser, an autosampler, and a binary pump. The LC separation was performed on an Agilent Agilent Eclipse XDB-C18 (4.6 x 150mm, 5 μm) with mobile phase A (0.1% formic acid in water) and mobile phase B (0.1% formic acid in MeCN). The flow rate was 0.4 mL min^−1^. The linear gradient was as follows: 0–2 min, 90%A; 8 min, 55%A; 13–25 min, 40%A; 30 min, 30%A; 35 min, 25%A; 36–44 min, 0%A; 45–50 min, 90%A. The autosampler was set at 10 °C. The injection volume was 10 μL. Mass spectra were acquired under positive electrospray ionization (ESI) with the ion spray voltage of +5000 V. The source temperature was 450 °C. The curtain gas, ion source gas 1, and ion source gas 2 were 32, 65, and 55, respectively. Multiple reaction monitoring (MRM) was used for quantitation: 14,15-epoNA5HT *m*/*z* 479.3 → *m*/*z* 160.0; 14,15-epoNADA *m*/*z* 456.3 → *m*/*z* 137.1; 5,6-EET-EA, 8,9-EET-EA, 11,12-EET-EA, and 14,15-EET-EA are all measured with *m*/*z* 264.2 → *m*/*z* 62.0. Internal standards Anadamide-d4 and 14,15-EET-EA-d8 were monitored at *m*/*z* 352.3 → *m*/*z* 287.2 and *m*/*z* 372.2 → *m*/*z* 63.0, respectively.

### Binding equations

The general one-site binding equation used was Eq. 1 below

1 $$B = B_0 + \frac{{B_{{\mathrm{max}}}[S]}}{{K + [S]}}$$

where *B*_*o*_ is the baseline response, *B*_max_ is the maximum response, *K* is the binding parameter, and [*S*] is the substrate concentration. For kinetic experiments, *B* and *K* represent velocity and *K*_*m*_, respectively; for Soret binding experiments, *B* and *K* represent Δ*A* and *K*_*D*_, respectively. For metabolism and Soret experiments, *B*_*o*_ = 0; for NADPH metabolism *B*_*o*_ is a nonzero value.

Inhibition experiments were described by either a competitive model (Eq. 2) or noncompetitive model (Eq. 3)

2 $$B = \frac{{B_{{\mathrm{max}}}[S]}}{{K\left( {1 + \frac{{[I]}}{{K_{\mathrm{i}}}}} \right) + [S]}}$$

3 $$B = \frac{{B_{{\mathrm{max}}}[S]}}{{\left( {K + [S]} \right)\left( {1 + \frac{{[I]}}{{K_{\mathrm{i}}}}} \right)}}$$

where *K*_i_ is the affinity of the inhibitor and [*I*] is the concentration of the inhibitor.

A general two-site binding equation (Eq. 4) was used to describe the metabolism of NADA and NA5HT

4 $$B = \frac{{\frac{{B_1[S]}}{{K_1}} + \frac{{B_2[S]^2}}{{K_1 \cdot K_2}}}}{{1 + \frac{{[S]}}{{K_1}} + \frac{{[S]^2}}{{K_1 \cdot K_2}}}}$$

where *B*_1_ and *B*_2_ are the maximum metabolism at the first site and second site, respectively, and *K*_1_ and *K*_2_ are the affinities at the first site and second site, respectively.

In cellulo data were fitted to a dose-response equation (Eq. 5)

5 $$B = B_0 + \frac{{B_{{\mathrm{max}}} - B_o}}{{1 + 10^{\left[ {\left( {B_{50} - \left[ L \right]} \right)} \right]p}}}$$

where [*L*] is the concentration of the ligand, *p* is the Hill coefficient, *B*_50_ is the half-maximal response (EC_50_ for agonism and IC_50_ for antagonism experiments), *B*_*o*_ is the baseline response (bottom asymptote), and *B*_max_ is the maximum response (top asymptote).

### Statistical analysis

Statistical significance was determined by either a two-tailed *t*-test with equal variance or a one-way ANOVA followed by a Tukey’s post-hoc analysis as indicated in each figure legend. *P*-values < 0.05 were considered statistically significant.

### Modeling and simulation of CYP2J2

Initial structural models of membrane-bound CYP2J2 bound to AEA and NADA or NA5HT were generated with molecular docking performed with AutoDock Vina^63^ in a stepwise manner as described below. A grid box of dimension 22 Å in *x*, *y,* and z and centered in the active site of CYP2J2 was employed for docking. We first docked NADA or NA5HT to our previous membrane-bound models of CYP2J2^36^. From this docking step, configurations of AEA/NADA/NA5HT in which the main epoxidation site (carbons C14 and C15) were close to the heme moiety (with distance <5 Å) and with a high docking score, resulting in over 100 initial structures of each molecule in complex with CYP2J2. The resulting structures were then employed as receptors for docking of a second molecule (AEA for NADA/NA5HT in active site, or NADA/NA5HT for AEA in active site). This second step allowed us to explore potential peripheral binding sites (i.e., outside the central active site cavity of CYP2J2), which were hypothesized from the experimental. From docking, two potential peripheral binding sites were identified. For each peripheral binding configuration, the corresponding CYP2J2 complexes with two molecules were sorted by docking score, and the models with the highest score were employed as starting configurations for MD simulations. Each simulation system was minimized for 2000 steps, and equilibrated for 1 ns with the *C*_α_ atoms of CYP2J2 and the heavy atoms of the ligands (AEA, NADA, and NA5HT) harmonically restrained (with force constant *k* = 1 kcal per mol per Å^2^). Following this preparation step, the 2-molecule systems were simulated for 50 ns.

### Simulation protocol

The simulations were performed using NAMD2^64^. The CHARMM27 force field with cMAP^48^ corrections was used for CYP2J2. The CHARMM36^65,66^ force field was used for lipids. Force field parameters for AEA, NADA, and NA5HT were generated by analogy from the CHARMM General Force Field^67^. The TIP3P model was used for water^68^. Simulations were performed with the NPT ensemble with a time step of 2 fs. A constant pressure of 1 atm was maintained using the Nosé-Hoover Langevin piston method^69^. Temperature was maintained at 310 K using Langevin dynamics with a damping coefficient *γ* of 0.5 ps^-1^ applied to all atoms. Nonbonded interactions were cutoff at 12 Å, with smoothing applied at 10 Å. The particle mesh Ewald (PME) method^70^ was used for long-range electrostatic calculations with a grid density of >1 Å^−3^.

### Reporting summary

Further information on research design is available in the Nature Research Reporting Summary linked to this article.

## Supplementary information

Supplementary Information

Description of Additional Supplementary Files

Supplementary Movie 1

Supplementary Movie 2

Supplementary Movie 3

Supplementary Movie 4

Reporting Summary

## Data Availability

All data generated or analyzed during this study are included in this published article (and its supplementary information files). Other material is available from the corresponding author on reasonable request. Material availability statement: All plasmids used in the study are available from Addgene or can be obtained from the corresponding author on request. All other data or resources are available from the corresponding author upon reasonable request. Source data are provided with this paper.

## Source data

Source Data

## References

[1] Hedegaard, H., Warner, M. & Minino, A. M. *Drug Overdose Deaths in the United States*, *1999–2016*. 1–8 (NCHS Data Brief*,* 2017). 29319475

[2] Park KA, Vasko MR (2005). Lipid mediators of sensitivity in sensory neurons. Trends Pharm. Sci..

[3] Cashman JN (1996). The mechanisms of action of NSAIDs in analgesia. Drugs.

[4] Serhan CN (2014). Pro-resolving lipid mediators are leads for resolution physiology. Nature.

[5] Serhan CN, Chiang N (2013). Resolution phase lipid mediators of inflammation: agonists of resolution. Curr. Opin. Pharmacol..

[6] Piomelli D, Sasso O (2014). Peripheral gating of pain signals by endogenous lipid mediators. Nat. Neurosci..

[7] Gerak LR, France CP (2016). Combined treatment with morphine and Delta(9)-tetrahydrocannabinol in rhesus monkeys: antinociceptive tolerance and withdrawal. J. Pharmacol. Exp. Therapeutics.

[8] Devane WA, Dysarz FA, Johnson MR, Melvin LS, Howlett AC (1988). Determination and characterization of a cannabinoid receptor in rat brain. Mol. Pharmacol..

[9] Munro S, Thomas KL, Abu-Shaar M (1993). Molecular characterization of a peripheral receptor for cannabinoids. Nature.

[10] Guindon J, Hohmann AG (2009). The endocannabinoid system and pain. CNS Neurol. Disord. Drug Targets.

[11] Amaya F (2006). Induction of CB1 cannabinoid receptor by inflammation in primary afferent neurons facilitates antihyperalgesic effect of peripheral CB1 agonist. Pain.

[12] Galiègue S (1995). Expression of central and peripheral cannabinoid receptors in human immune tissues and leukocyte subpopulations. Eur. J. Biochem..

[13] Clayton N, Marshall FH, Bountra C, O'Shaughnessy CT (2002). CB1 and CB2 cannabinoid receptors are implicated in inflammatory pain. Pain.

[14] Guindon J, Hohmann AG (2008). Cannabinoid CB2 receptors: a therapeutic target for the treatment of inflammatory and neuropathic pain. Br. J. Pharmacol..

[15] Morales P, Hurst DP, Reggio PH (2017). Molecular targets of the phytocannabinoids: a complex picture. Prog. Chem. Org. Nat. Pr..

[16] Caterina MJ (2000). Impaired nociception and pain sensation in mice lacking the capsaicin receptor. Science.

[17] Choi SI, Yoo S, Lim JY, Hwang SW (2014). Are sensory TRP channels biological alarms for lipid peroxidation?. Int. J. Mol. Sci..

[18] Anand P, Bley K (2011). Topical capsaicin for pain management: therapeutic potential and mechanisms of action of the new high-concentration capsaicin 8% patch. Br. J. Anaesth..

[19] Yoshida, A. et al. TRPV1 is crucial for proinflammatory STAT3 signaling and thermoregulation-associated pathways in the brain during inflammation. *Sci. Rep.* **6**, 26088 (2016). 10.1038/srep26088 PMC4870621 27188969

[20] Grabiec U, Dehghani F (2017). N-arachidonoyl dopamine: a novel endocannabinoid and endovanilloid with widespread physiological and pharmacological activities. Cannabis Cannabinoid Res..

[21] Devane WA (1992). Isolation and structure of a brain constituent that binds to the cannabinoid receptor. Science.

[22] Bisogno T (2000). N-acyl-dopamines: novel synthetic CB(1) cannabinoid-receptor ligands and inhibitors of anandamide inactivation with cannabimimetic activity in vitro and in vivo. Biochem. J..

[23] Bisogno T (1998). Arachidonoylserotonin and other novel inhibitors of fatty acid amide hydrolase. Biochem. Biophys. Res. Commun..

[24] Siller M (2014). Oxidation of endogenous N-arachidonoylserotonin by human cytochrome P450 2U1. J. Biol. Chem..

[25] Verhoeckx KC (2011). Presence, formation and putative biological activities of N-acyl serotonins, a novel class of fatty-acid derived mediators, in the intestinal tract. Biochim. Biophys. Acta.

[26] Rimmerman N (2009). Microsomal omega-hydroxylated metabolites of N-arachidonoyl dopamine are active at recombinant human TRPV1 receptors. Prostaglandins Other Lipid Mediat..

[27] Maione S (2007). Analgesic actions of N-arachidonoyl-serotonin, a fatty acid amide hydrolase inhibitor with antagonistic activity at vanilloid TRPV1 receptors. Br. J. Pharmacol..

[28] Yoo JM, Sok DE, Kim MR (2013). Effect of endocannabinoids on IgE-mediated allergic response in RBL-2H3 cells. Int. Immunopharmacol..

[29] Schunck WH, Konkel A, Fischer R, Weylandt KH (2018). Therapeutic potential of omega-3 fatty acid-derived epoxyeicosanoids in cardiovascular and inflammatory diseases. Pharm. Ther..

[30] Zhang G, Kodani S, Hammock BD (2014). Stabilized epoxygenated fatty acids regulate inflammation, pain, angiogenesis and cancer. Prog. Lipid Res..

[31] Zelasko S, Arnold WR, Das A (2015). Endocannabinoid metabolism by cytochrome P450 monooxygenases. Prostaglandins Other Lipid Mediat..

[32] McDougle DR (2017). Anti-inflammatory omega-3 endocannabinoid epoxides. Proc. Natl Acad. Sci. USA.

[33] Roy, J., Watson, J. E., Hong, I., Fan, T. M. & Das, A. Anti-tumorigenic properties of omega-3 endocannabinoid epoxides. *J. Med. Chem.*10.1021/acs.jmedchem.8b00243 (2018). 10.1021/acs.jmedchem.8b00243 PMC7202735 29856219

[34] Arnold WR, Weigle AT, Das A (2018). Cross-talk of cannabinoid and endocannabinoid metabolism is mediated via human cardiac CYP2J2. J. Inorg. Biochem..

[35] McDougle DR, Kambalyal A, Meling DD, Das A (2014). Endocannabinoids anandamide and 2-arachidonoylglycerol are substrates for human CYP2J2 epoxygenase. J. Pharm. Exp. Ther..

[36] Arnold WR, Baylon JL, Tajkhorshid E, Das A (2016). Asymmetric binding and metabolism of polyunsaturated fatty acids (PUFAs) by CYP2J2 epoxygenase. Biochemistry.

[37] Seki H, Tani Y, Arita M (2009). Omega-3 PUFA derived anti-inflammatory lipid mediator resolvin E1. Prostaglandins Other Lipid Mediat..

[38] Inoue K, Tsuda M (2018). Microglia in neuropathic pain: cellular and molecular mechanisms and therapeutic potential. Nat. Rev. Neurosci..

[39] Snider NT, Nast JA, Tesmer LA, Hollenberg PF (2009). A cytochrome P450-derived epoxygenated metabolite of anandamide is a potent cannabinoid receptor 2-selective agonist. Mol. Pharmacol..

[40] Graber MN, Alfonso A, Gill DL (1997). Recovery of Ca2+ pools and growth in Ca2+ pool-depleted cells is mediated by specific epoxyeicosatrienoic acids derived from arachidonic acid. J. Biol. Chem..

[41] Atkins WM (2005). Non-Michaelis-Menten kinetics in cytochrome P450-catalyzed reactions. Annu. Rev. Pharmacol. Toxicol..

[42] Denisov IG, Frank DJ, Sligar SG (2009). Cooperative properties of cytochromes P450. Pharm. Ther..

[43] Graves JP (2015). Quantitative polymerase chain reaction analysis of the mouse Cyp2j subfamily: tissue distribution and regulation. Drug Metab. Dispos..

[44] Ferguson CS, Tyndale RF (2011). Cytochrome P450 enzymes in the brain: emerging evidence of biological significance. Trends Pharm. Sci..

[45] Wu S, Moomaw CR, Tomer KB, Falck JR, Zeldin DC (1996). Molecular cloning and expression of CYP2J2, a human cytochrome P450 arachidonic acid epoxygenase highly expressed in heart. J. Biol. Chem..

[46] Snider NT, Nast JA, Tesmer LA, Hollenberg PF (2009). A cytochrome P450-derived epoxygenated metabolite of anandamide is a potent cannabinoid receptor 2-selective agonist. Mol. Pharm..

[47] Musella, A. et al. A novel crosstalk within the endocannabinoid system controls GABA transmission in the striatum. *Sci. Rep.* **7**, 7363 (2017). 10.1038/s41598-017-07519-8 PMC5544685 28779174

[48] Mackerell AD, Feig M, Brooks CL (2004). Extending the treatment of backbone energetics in protein force fields: limitations of gas-phase quantum mechanics in reproducing protein conformational distributions in molecular dynamics simulations. J. Comput. Chem..

[49] Carnevale, L., Arango, A., Arnold, W. R., Tajkhorshid, E. & Das, A. Endocannabinoid virodhamine is an endogenous inhibitor of human cardiovascular CYP2J2 epoxygenase. *Biochemistry*10.1021/acs.biochem.8b00691 (2018). 10.1021/acs.biochem.8b00691 PMC6262108 30285425

[50] Arnold WR, Baylon JL, Tajkhorshid E, Das A (2017). Arachidonic acid metabolism by human cardiovascular CYP2J2 is modulated by doxorubicin. Biochemistry.

[51] Lawton SK (2017). N-arachidonoyl dopamine modulates acute systemic inflammation via nonhematopoietic TRPV1. J. Immunol..

[52] Wilhelmsen K (2014). The endocannabinoid/endovanilloid N-arachidonoyl dopamine (NADA) and synthetic cannabinoid WIN55,212-2 abate the inflammatory activation of human endothelial cells. J. Biol. Chem..

[53] Costa B (2010). The dual fatty acid amide hydrolase/TRPV1 blocker, N-arachidonoyl-serotonin, relieves carrageenan-induced inflammation and hyperalgesia in mice. Pharmacol. Res..

[54] Di Marzo V, Bifulco M, De Petrocellis L (2004). The endocannabinoid system and its therapeutic exploitation. Nat. Rev. Drug Discov..

[55] De Petrocellis, L. & Di Marzo, V. in *The World of Endocannabinoids and Related Mediators* 67–84 (Elsevier, 2015).

[56] Korzekwa KR (1998). Evaluation of atypical cytochrome P450 kinetics with two-substrate models: evidence that multiple substrates can simultaneously bind to cytochrome P450 active sites. Biochemistry.

[57] Graves JP (2017). Characterization of the tissue distribution of the mouse Cyp2c subfamily by quantitative PCR analysis. Drug Metab. Dispos..

[58] Nishimura M, Yaguti H, Yoshitsugu H, Naito S, Satoh T (2003). Tissue distribution of mRNA expression of human cytochrome P450 isoforms assessed by high-sensitivity real-time reverse transcription PCR. Yakugaku zasshi : J. Pharm. Soc. Jpn..

[59] Dutheil F (2009). Xenobiotic-metabolizing enzymes and transporters in the normal human brain: regional and cellular mapping as a basis for putative roles in cerebral function. Drug Metab. Disposition.

[60] Ruparel S (2012). Plasticity of cytochrome P450 isozyme expression in rat trigeminal ganglia neurons during inflammation. Pain.

[61] Gao Y, Cao E, Julius D, Cheng Y (2016). TRPV1 structures in nanodiscs reveal mechanisms of ligand and lipid action. Nature.

[62] Chen, J. et al. Spatial distribution of the cannabinoid type 1 and capsaicin receptors may contribute to the complexity of their crosstalk. *Sci. Rep.* **6**, 33307 (2016). 10.1038/srep33307 PMC5032030 27653550

[63] Trott O, Olson AJ (2010). AutoDock Vina: improving the speed and accuracy of docking with a new scoring function, efficient optimization, and multithreading. J. Comput. Chem..

[64] Phillips JC (2005). Scalable molecular dynamics with NAMD. J. Comput. Chem..

[65] Hart K (2012). Optimization of the CHARMM additive force field for DNA: Improved treatment of the BI/BII conformational equilibrium. J. Chem. Theory Comput.

[66] Klauda JB (2010). Update of the CHARMM all-atom additive force field for lipids: validation on six lipid types. J. Phys. Chem. B.

[67] Vanommeslaeghe K (2010). CHARMM general force field: a force field for drug-like molecules compatible with the CHARMM all-atom additive biological force fields. J. Comput. Chem..

[68] Jorgensen WL, Chandrasekhar J, Madura JD, Impey RW, Klein ML (1983). Comparison of simple potential functions for simulating liquid water. J. Chem. Phys..

[69] Feller SE, Zhang YH, Pastor RW, Brooks BR (1995). Constant-pressure molecular-dynamics simulation-the langevin piston method. J. Chem. Phys..

[70] Darden T, York D, Pedersen L (1993). Particle mesh ewald-an N.Log(N) method for ewald sums in large systems. J. Chem. Phys..

