# Supplementary material for: Anti-inflammatory dopamine- and serotonin-based endocannabinoid epoxides reciprocally regulate cannabinoid receptors and the TRPV1 channel
Source: Nat Commun. 2021 Feb 10;12:926. doi: 10.1038/s41467-021-20946-6 (PMC7876028; doi:10.1038/s41467-021-20946-6)
Supplement: Supplementary file 1 — Supplementary Information [file 41467_2021_20946_MOESM1_ESM.pdf]

Supplementary Information

Anti-inflammatory dopamine- and serotonin-based endocannabinoid epoxides reciprocally regulate cannabinoid receptors and TRPV1 channel

William R. Arnold[1§], Lauren N. Carnevale[1§], Zili Xie[2], Javier L. Baylon[1,3,4,5,6], Emad Tajkhorshid[1,3,4,5,6], Hongzhen Hu[2] and Aditi Das[,†,1,3,5,6]*

[†]Department of Comparative Biosciences, [1]Department of Biochemistry, [2]Department of Anesthesiology, The Center for the Study of Itch & Sensory Disorders, Washington University School of Medicine, [3]Center for Biophysics and Quantitative Biology, [4]Center for Macromolecular Modeling and Bioinformatics, [5]Beckman Institute for Advanced Science and Technology, [6]Department of Bioengineering, Neuroscience program, University of Illinois Urbana-Champaign, Urbana IL 61801

Corresponding Author

* To whom correspondence should be addressed:

Aditi Das, Ph.D., University of Illinois Urbana-Champaign, 3836 VMBSB, 2001 South Lincoln Avenue, Urbana IL 61802, Phone: 217-244-0630. aditidas@illinois.edu

§ These authors have contributd equally to the manuscript

Table of Contents

Methods ... **4**

Notes 1 and 2 ... **9**

Figures ... **11**

Supplementary Figure 1 ... 11
Supplementary Figure 2 ... 12
Supplementary Figure 3 ... 13
Supplementary Figure 4 ... 14
Supplementary Figure 5 ... 15
Supplementary Figure 6 ... 16
Supplementary Figure 7 ... 18
Supplementary Figure 8 ... 19
Supplementary Figure 9 ... 20
Supplementary Figure 10 ... 21
Supplementary Figure 11 ... 22
Supplementary Figure 12 ... 23
Supplementary Figure 13 ... 24
Supplementary Figure 14 ... 25
Supplementary Figure 15 ... 26
Supplementary Figure 16 ... 27
Supplementary Figure 17 ... 28
Supplementary Figure 18 ... 29
Supplementary Figure 19 ... 30
Supplementary Figure 20 ... 31
Supplementary Figure 21 ... 32
Supplementary Figure 22 ... 33
Supplementary Figure 23 ... 34
Supplementary Figure 24 ... 35
Supplementary Figure 25 ... 36
Supplementary Figure 26 ... 37
Supplementary Figure 27 ... 38
Supplementary Figure 28 ... 39
Supplementary Figure 29 ... 40
Supplementary Figure 30 ... 41
Supplementary Figure 31 ... 42

Supplementary Figure 32 43
Supplementary Figure 33 44
Supplementary Figure 34 45
Supplementary Figure 35 46
Supplementary Figure 36 47
Tables 48
Supplementary Table 1 48
Supplementary Table 2 49
Supplementary Table 3 52
Supplementary Table 4 53
Supplementary Table 5 54
Supplementary Table 6 56
Movies 58
Supplementary Movie 1 58
Supplementary Movie 2 58
Supplementary Movie 3 58
Supplementary Movie 4 58
References 59

Methods

Materials. Human CYP2J2 cDNA was obtained from OriGene (Catalog No. SC321730) and modified as published before[1]. Ampicillin, arabinose, chloramphenicol, isopropyl β-D-1-thiogalactopyranoside (IPTG), and Ni-NTA resin were obtained from Gold Biotechnology. δ-aminolevulinic acid was obtained from Frontier Scientific. NADPH and $NADP^+$ were obtained from P212121.com. 1-palmitoyl-2-oleoyl-sn-glycero-3-phosphocholine (POPC) and 1-hexadecanoyl-2-(9Z-octadecenoyl)-sn-glycero-3-phospho-L serine (POPS) were purchased from Avanti Polar Lipids, Inc. AA, NADA, and NA5HT, SKF 525A (α-phenyl-α-propyl-benzeneacetic acid, 2-(diethylamino)ethyl ester, monohydrochloride), AMG-9810 ((2E)-N-(2,3-dihydro-1,4-benzodioxin-6-yl)-3-[4-(1,1-dimethylethyl)phenyl]-2-propenamide), capsaicin (N-[(4-hydroxy-3-methoxyphenyl)methyl]-6E-8-methyl-nonenamide), and CP55940 (*rac*-5-(1,1-dimethylheptyl)-2-[(1R,2R,5R)-5-hydroxy-2-(3-hydroxypropyl)cyclohexyl]-phenol) were obtained from Cayman Chemical. AA for syntheses was obtained from NuChek. All other materials and reagents used were purchased from Sigma-Aldrich and Fisher Scientific.

Reverse Transcription quantitative Polymerase Chain Reaction (RT-qPCR). To measure IL-6, IL-10, IL-1β, TNF-α, CYP2J9, CYP2J12, GAPDH, TRPV1, CB1, and CB2 mRNA, total RNA was isolated from BV2 microglial cells using the Direct-zol RNA Kit, according to the manufacturer's instructions (Zymo Research). One microgram of RNA and random primers were used to synthesize the first strand of cDNA using the High-Capacity cDNA Reverse Transcription Kit (Applied Biosciences). Total cDNA reaction samples were diluted 5-fold in RNAse/DNase-free water and were used as templates for amplification of each gene using the 7500 Real Time PCR System (Applied Biosciences). In addition, corresponding primers purchased from Integrated DNA Technologies (Supplementary Table 1) and the Power SYBR green PCR Master Mix (Applied Biosciences) were utilized during each qPCR reaction. The mRNA levels of IL-6, IL-10, IL-1β, TNF-α, CYP2J9, CYP2J12, TRPV1, CB1, and CB2 were

normalized to those of GAPDH, the internal control, and were analyzed using the $2^{-\Delta\Delta C_T}$ method.[2] Relative mRNA expression was calculated by taking the quotient of the treated sample (LPS + eVD) and LPS alone (IL-6, IL-10, IL-1β, and TNF-α mRNA) or the quotient of LPS-treated and LPS-depleted samples (CYP2J9, CYP2J12, TRPV1, CB1, and CB2). Graphs show the relative mRNA expression of each sample for a given gene. The asterisks above each bar indicate the p-value associated with the standard deviation of each triplicate ΔCT value. P-values were calculated using a two-tailed t-test ($\alpha = 0.05$): 0.0332 (*), 0.0021(**), 0.0002 (***), and < 0.0001 (****).Primers used were designed as shown in Supplementary Table 1.

BV2 Anti-inflammatory and Cytotoxicity Assays (Griess assay, IL-6 ELISA, and MTT). Assays were performed as previously described[3]. BV2 cells were seeded in 24-well plates (200,000 cells/mL) and grown to 80-90% confluency. For eVD screening and dose response studies, media was replaced with serum-free media and the cells were pre-incubated with eVDs for 4 hours prior to stimulation with 25 ng/mL LPS (Sigma-Aldrich, USA). For epoxy-eVDs studies, BV2 were treated with the sEH inhibitor, t-AUCB (1 µM), for 30 min prior to addition of epoxy-eVDs. For inhibition studies, CB1 antagonist, Rimonabant (1 µM), CB2 inverse agonist, AM630 (1 µM), TRPV1 antagonist, AMG 9810 (1 µM), were pre-incubated 30 min before addition of eVDs (1 µM) for 4 hrs. Media was collected after 24 hours and measured for NO levels (Griess assay) and IL-6 levels (ELISA) as previously described[3]. Cellular proliferation/cytotoxicity was assessed by testing the media with a commercially available MTT kit (Cayman Chemical Item No: #10009365).

BrdU Cell Proliferation Assay. The BV-2 cells were seeded at 20,000 cells/0.1 mL in a 96-well plate. For eVD screening and dose response studies, media was replaced with serum-free media and the cells were pre-incubated with eVDs for 4 hours prior to stimulation with 25 ng/mL LPS for an additional 24 hours (final well vol. 200 µL). For epoxygenated metabolites, serum-media was replaced with media containing 1 µM of sEH inhibitor (t-AUCB) for 30 min before adding epoVDs and LPS stimulation.

Briefly, after a 21 h incubation, BrdU (1x) was added to the plate for 3 hours. Next, the media was aspirated and the BV-2 cells were fixed according to manufacturer's instruction (Millipore Sigma, Cat. No. 2750). BrdU incorporation was detected by the addition of anti-BrdU monoclonal antibody with Goat anti-mouse IgG peroxidase conjugate. Lastly, TMB substrate solution was added for 30 min at room temperature and stop solution was added. The positive wells were measured using a Biotek Synergy 2 microplate reader (Biotek Instruments, Winooski, VT, USA) at an absorbance wavelength of 450 nm.

Expression and purification of recombinant CYP2J2 in *E. coli.* Recombinant D34-CYP2J2 containing a His_5 tag was expressed and purified as previously performed[1,4]. The D34-CYP2J2 is a 34-residue N-terminal truncation (residues 3-37) of CYP2J2 with a substitution of Leu2 for an Ala residue. These modifications have been previously shown to increase protein yield without affecting activity[38, 45].

Expression and purification of cytochrome P450 reductase. Expression of cytochrome P450 reductase (CPR) was performed as described previously[1].

Use of reactive oxygen species (ROS) scavengers. All *in vitro* metabolism data using recombinant proteins CYP2J2-CPR in nanodiscs were performed using ROS scavengers as previously described[5].

CYP2J2-mediated metabolism of NADA and NA5HT. Metabolism of NADA and NA5HT was performed in a lipid reconstituted system as previously described[5,6]. Briefly, 0.6 μM CYP2J2 and 0.6 μM CPR were incubated in a 20% POPS and 80% POPC reconstituted system in 0.5 mL of 0.1 M potassium phosphate buffer, pH 7.4 at 37° C with 100 μM NADA or NA5HT for 10 min. Reactions were initiated with 1 mM NADPH (final) and reacted for 60 min. Reactions were terminated by vortexing with 0.5 mL ethyl acetate. Adequate amount of $NaCl_{(s)}$ was added to facilitate layer separation. Metabolites were extracted thrice by vortexing the reactions for 1 min with 0.5 mL ethyl acetate followed by centrifugation at 1,984 x g at 4° C for

5 minutes to separate the layers. The organic layers were dried under a stream of $N_{2(g)}$ and resuspended in 0.1 mL of acetonitrile for HPLC and LC-MS/MS analysis. For all other experiments, CYP2J2 was incorporated into Nanodiscs.

Incorporation of CYP2J2 into Nanodiscs. Nanodiscs (NDs) containing CYP2J2 were prepared in 20% POPS and 80% POPC Nanodiscs as previously described[7]. NDs were used for all experiments using *in vitro* CYP2J2 except for initial product determination.

Kinetics of NADA and NA5HT metabolism. The kinetics of NADA and NA5HT metabolism was determined using a CYP2J2- ND/CPR system as previous described[7] with the following modifications. Reactions were performed with ROS scavengers as stated above. NADA and NA5HT (10-100 μM in DMSO) were incubated with CYP2J2-ND/CPR in 0.5 mL of 0.1 M potassium phosphate buffer (pH 7.4) for 10 min. Reactions were initiated with the addition of 0.5 mM NADPH. The formation of epoxy-eVDs was determined to be linear up to 45 min, and so a 30-min reaction was utilized. Reactions were terminated upon the addition of 0.5 mL ethyl acetate and approximately 10 mg $NaCl_{(s)}$ to facilitate layer separation. The products were extract thrice with ethyl acetate, dried under a stream of $N_{2(g)}$, and resuspended in 180-proof ethanol for LC-MS/MS quantification.

NADPH kinetics. The rate of NADPH oxidation by CYP2J2-ND/CPR was determined using UV-Vis spectroscopy as previously described[8]. The rate of the NADPH oxidation with CYP2J2-ND/CPR without substrates was considered the "baseline" rate.

AEA metabolism inhibition. AEA metabolism was determined as previously described[6] with the following modifications. Reactions were performed in the presence of ROS scavengers as stated above and were terminated using 0.5 mL ethyl acetate and $NaCl_{(s)}$. 25 μM and 75 μM of NADA or NA5HT were used to inhibit AEA metabolism. 40 μM of AEA without inhibitor was

used as a control control to compare against our previous measurements for AEA metabolism[6]. The levels of AEA metabolism were similar to previously published and were used to assess saturation level (v/V_{max})[6]. EET-EAs and epoxy-eVDs were simultaneously quantified using LC-MS/MS.

Ebastine competitive binding. Relative binding affinities of eVDs binding to CYP2J2 were determined using an ebastine competitive binding assay as previously described.[7]

LC-MS and LC-MS/MS analysis of CYP2J2 metabolites. CYP2J2 metabolism products were determined using HPLC Method 1. The Phenomenex column was used. LC-MS/MS detection was performed as previously described[6].

Supplementary Note 1

Previously, NADA was found in the cerebellum and hippocampus/thalamus regions in bovine and rat brains[9-11], while NA5HT was identified in bovine brain extracts and in the intestine[12,13]. The levels of NADA and NA5HT have been reported to be <1-10 pmol · g^{-1} in these brain tissues, which is lower than literature AEA levels (~20-80 pmol · g^{-1})[14] and lower than our reported levels for AEA in rat brains (150 pmol · g^{-1}) and pig brains (2800 pmol · g^{-1})[3]. AEA levels are known to rise postmortem due to denaturation of the enzymes that degrade AEA[15]. Additionally, during tissue extraction, NADA and NA5HT are subjected to potential oxidation of the headgroups and their levels are expected to be lower than AEA. Of note, two separate extraction methods were used to detect eVDs and AEA (see methods). AEA was not well detected using the methanol/C-18 cartridges and the eVDs quickly degraded during the acetate:hexane extraction. Therefore, we cannot simultaneously quantify AEA and eVDs in the same sample.

Supplementary Note 2

Estimate of NA5HT binding at the second site

We can definitively demonstrate that the eVDs bind to at least two sites in CYP2J2 from the kinetic curves; however, due to the solubility issues of the lipids, we cannot determine the kinetic parameters of their metabolism. Notwithstanding, we can obtain approximate values for the kinetic parameters of NA5HT based on a two-site binding equation using assumptions from other data (Equation 4). The assumptions are the following. (A) Negligible metabolism occurs at the unproductive site ($B_1 = 0$). (B) The EBS competitive binding and NADPH oxidation data represent the affinity at the first site and can be averaged to obtain $K_1 = 38$ µM. (C) AEA potentiates the metabolism of 75 µM NA5HT by almost 100-fold with an apparent $V_{\max}$ and K_{m}

of 5.46 $\pm$ 1.91 nmol · min^{-1} · nmol$_{CYP2J2}^{-1}$ and 53.7 $\pm$ 35.7 μM, respectively. The apparent V_{max} as AEA potentiates the metabolism of NA5HT may represent the V_{max} of NA5HT metabolism (i.e., $B_2 = 5{,}460$ pmol · min^{-1} · nmol$_{CYP2J2}^{-1}$). Substituting these values into Equation 4, K_2 is determined to be 5.36 $\pm$ 0.27 mM (Supplementary Figure 30). This demonstrates that the affinity of NA5HT for the second site is weak, which matches the observations of the data. There is not enough details in the NADA data to perform a similar analysis, but it is likely that NADA binds weakly to its second site as well.

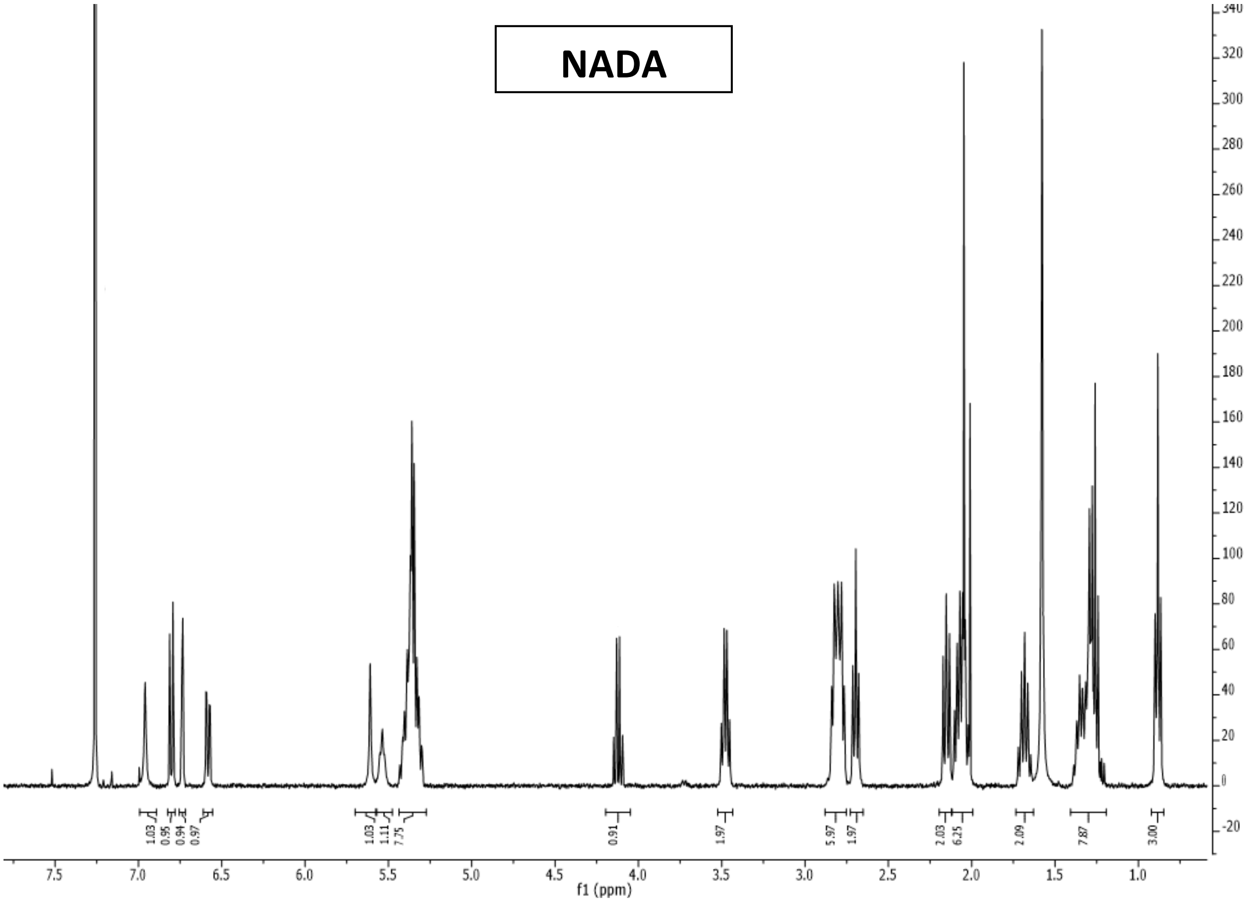

Supplementary Figure 1. ^{1}H-NMR spectrum for NADA (400 Hz, $CDCl_3$). Peak at 4.12 ppm corresponds to residual ethyl acetate. Other peaks for ethyl acetate overlap with NADA peaks (ethyl acetate: 2.05 and 1.26 ppm in $CDCl_3$)

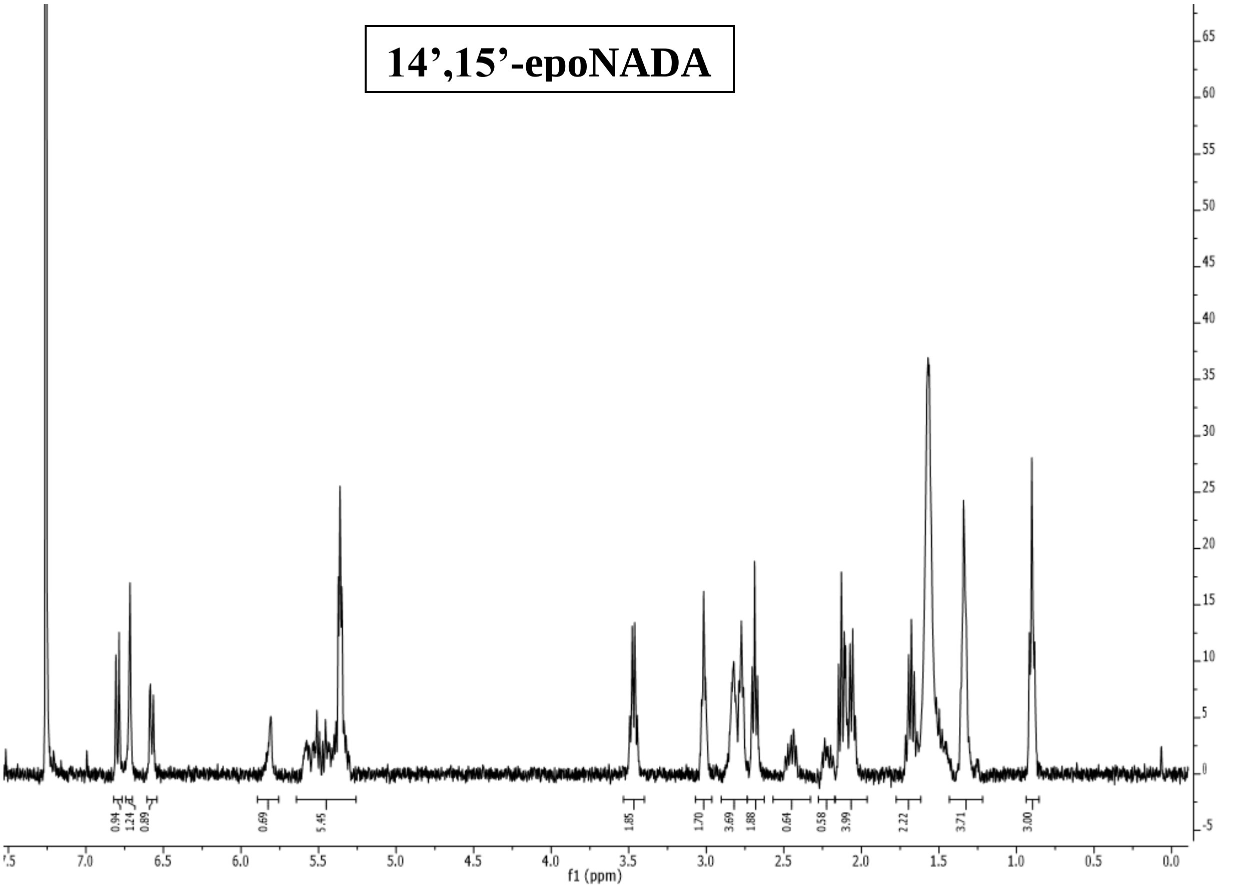

Supplementary Figure 2. ^{1}H-NMR spectrum for 14’,15’-epoNADA (400 Hz, $CDCl_3$).

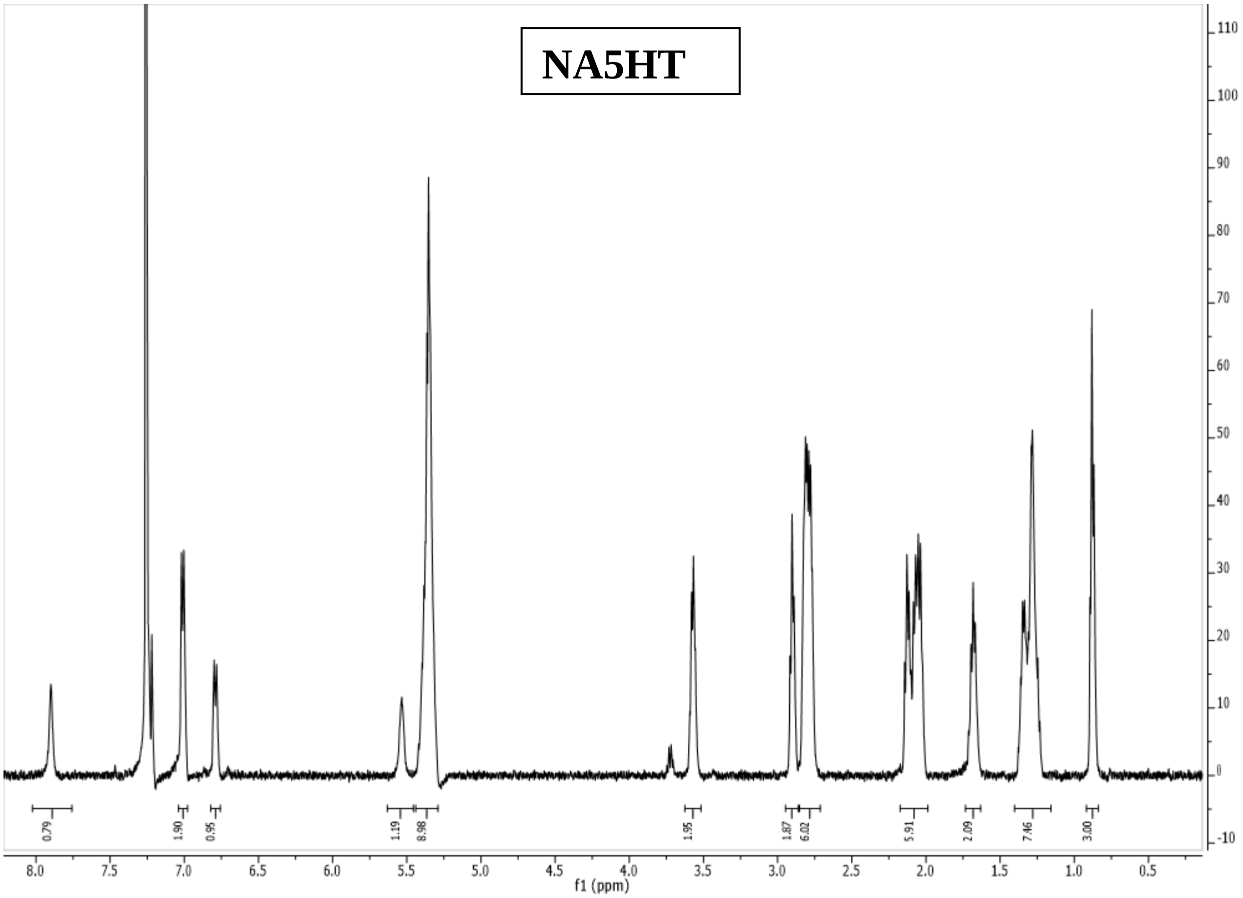

Supplementary Figure 3. ^{1}H-NMR spectrum for NA5HT (400 Hz, $CDCl_3$).

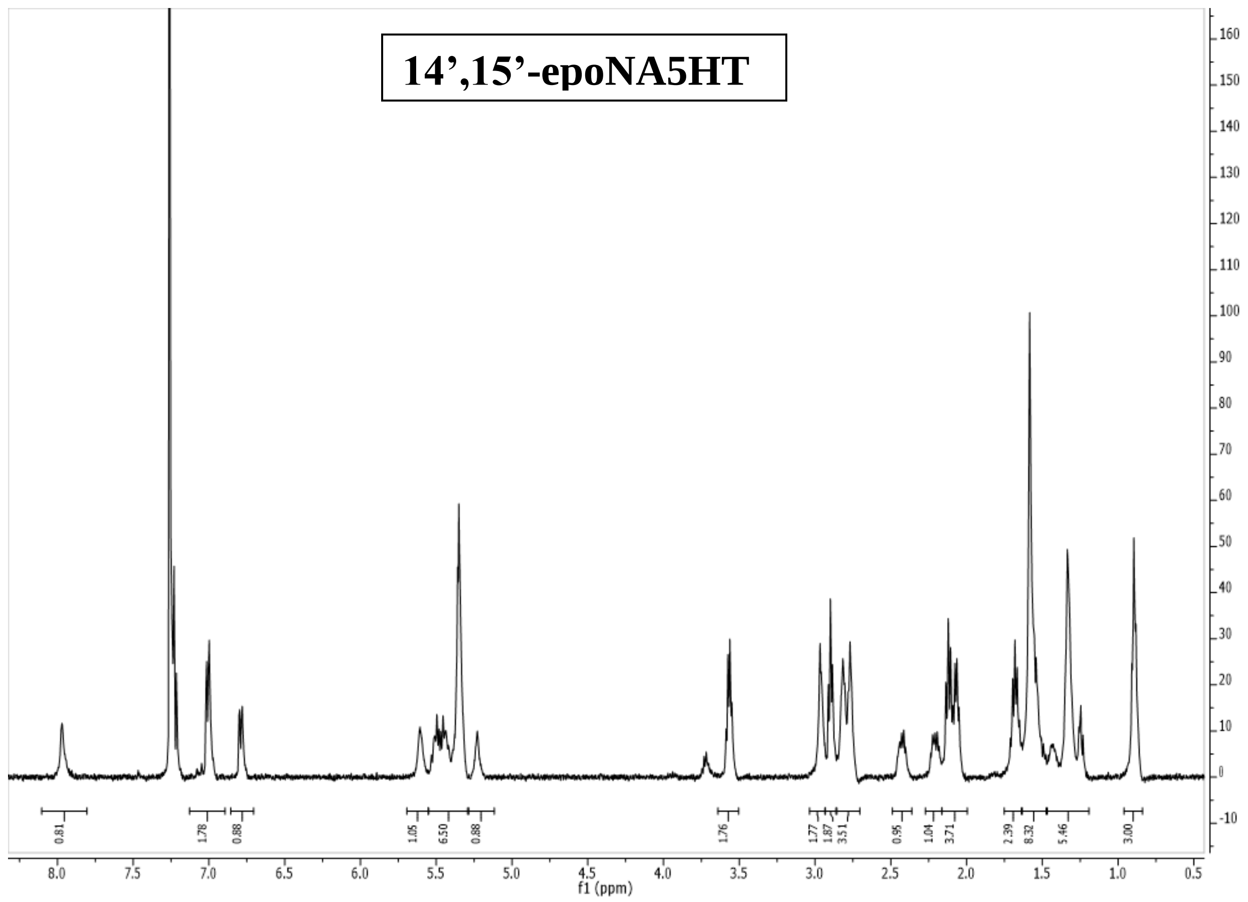

Supplementary Figure 4. ^{1}H-NMR spectrum for 14’,15’-epoNA5HT (500 Hz, $CDCl_3$).

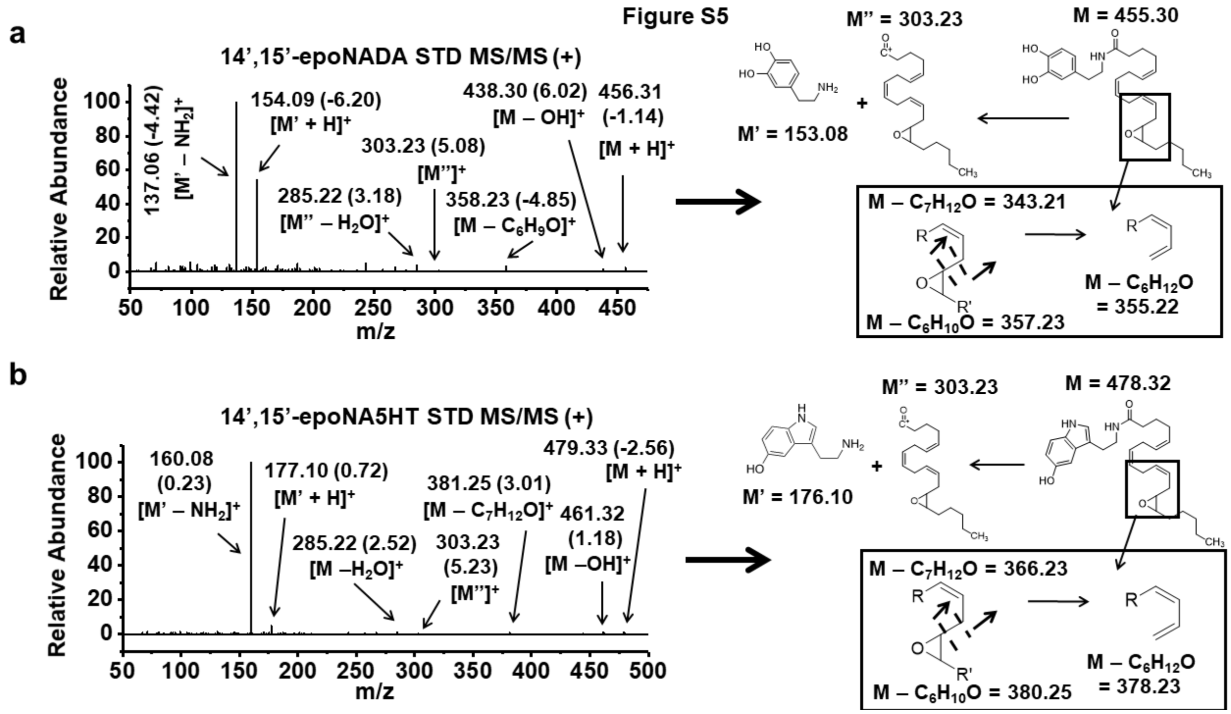

Supplementary Figure 5. MS/MS spectra of synthesized **(a)** 14',15'-epoNADA and **(b)** 14',15'-epoNA5HT standards along with fragmentation schemes. Data are obtained from LC-MS/MS analysis. Fragment m/z values are given with deviation from the calculated m/z values (± ppm) given in parentheses.

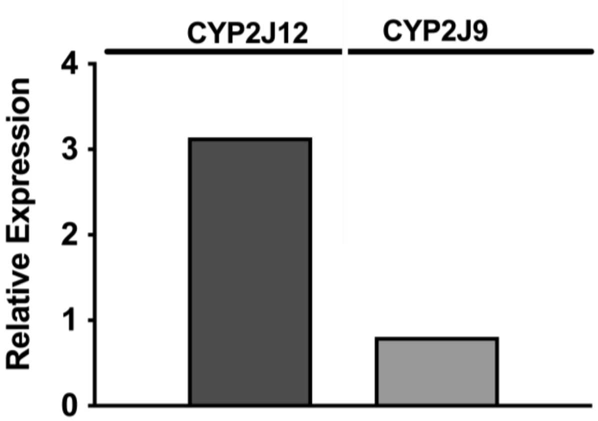

Supplementary Figure 6. *Effect of LPS in BV2 microglial cells on the relative mRNA expression of CYP2J12 and CYP2J9.* BV2 microglial cells seeded at 200,000 cells/well in a 24-well plate were treated with 100 ng/mL LPS for 3 hours. RT-qPCR detected the presence of CYP2J12 and CYP2J9 in BV2 cells. Data is from 3-pooled wells and is performed in technical replicates. Data is reported as the mean relative expression. Data can be found in the Source Data file.

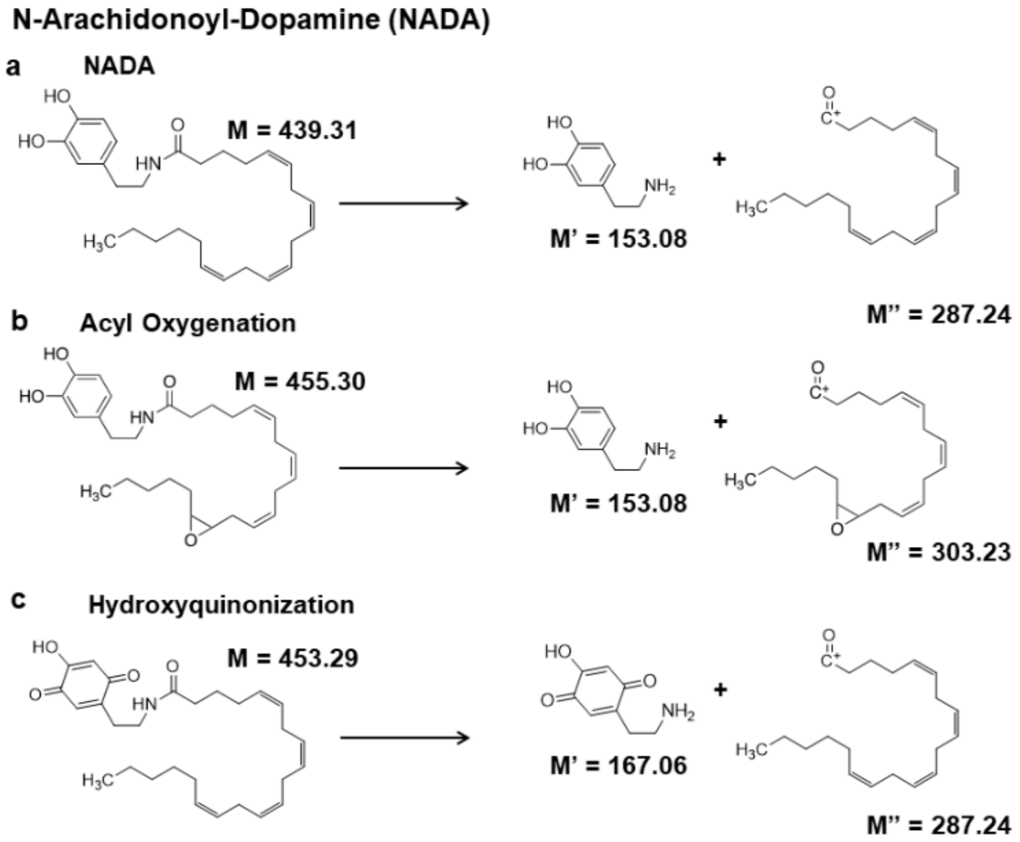

Supplementary Figure 7. Fragmentation scheme of **(a)** NADA, **(b)** acyl oxygenation of NADA (14',15'-epoNADA shown as an example), and **(c)** NADA-HQ.

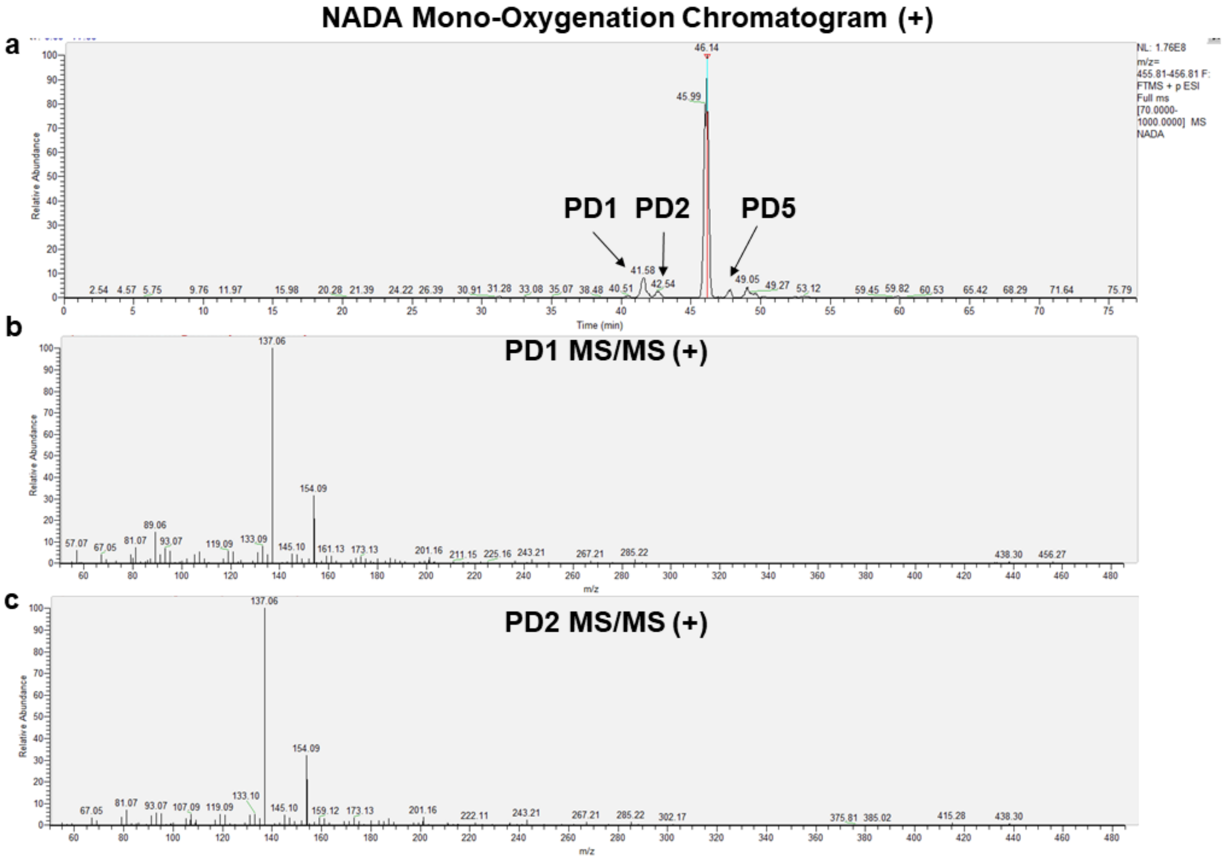

Supplementary Figure 8. *Mono-oxygenation of NADA by CYP2J2*. **(a)** LC-MS/MS chromatogram in positive ion mode. Mass range is ± 5 ppm of the predicted product. **(b-c)** MS/MS spectra obtained from each corresponding product.

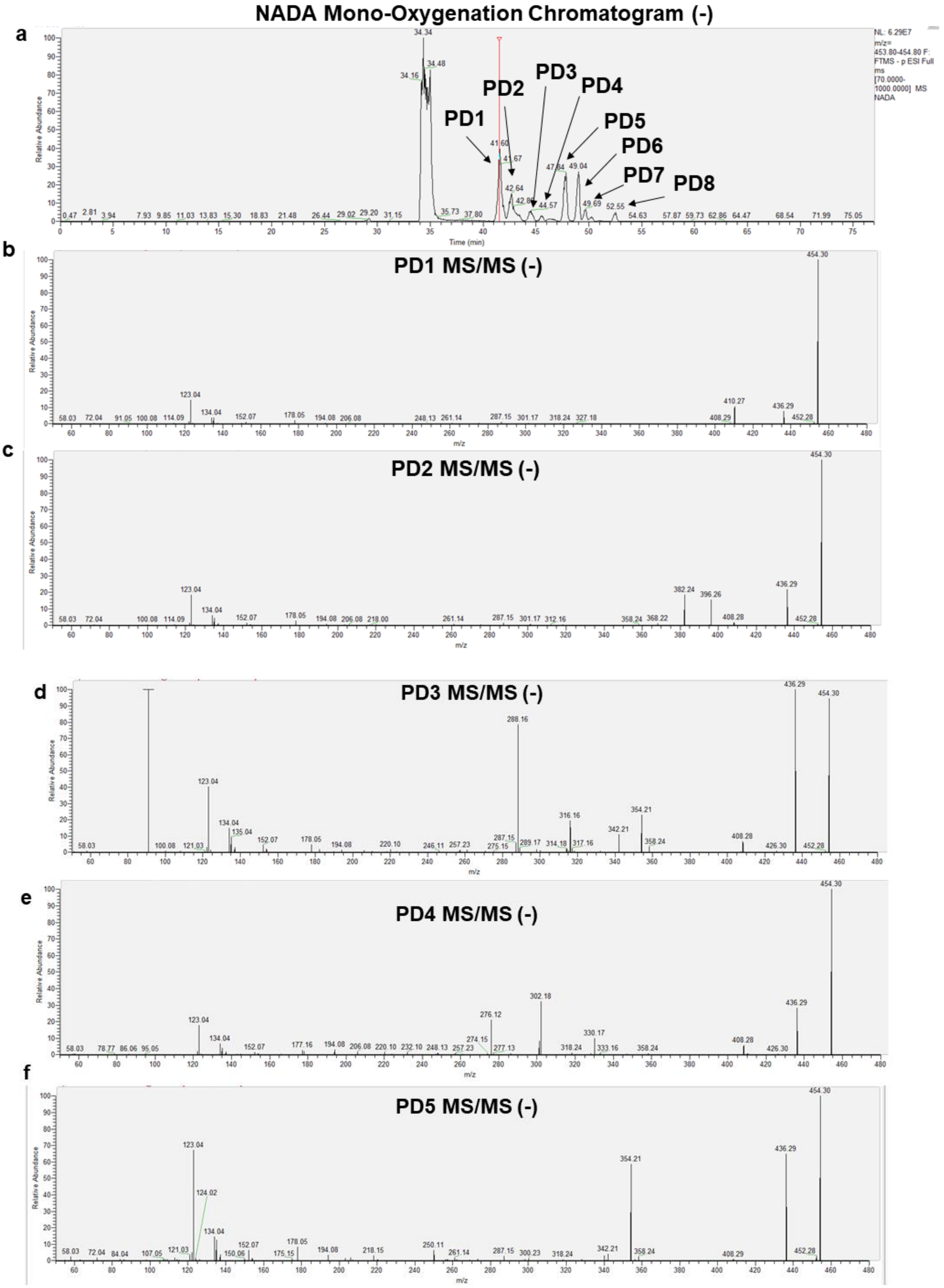
NADA Mono-Oxygenation Chromatogram (-)
a
PD1
PD2
PD3
PD4
PD5
PD6
PD7
PD8
NL: 6.29E7
m/z= 453.80-454.80 F: FTMS - p ESI Full ms [70.0000-1000.0000] MS NADA
Relative Abundance
Time (min)
b
PD1 MS/MS (-)
c
PD2 MS/MS (-)
d
PD3 MS/MS (-)
e
PD4 MS/MS (-)
f
PD5 MS/MS (-)
m/z

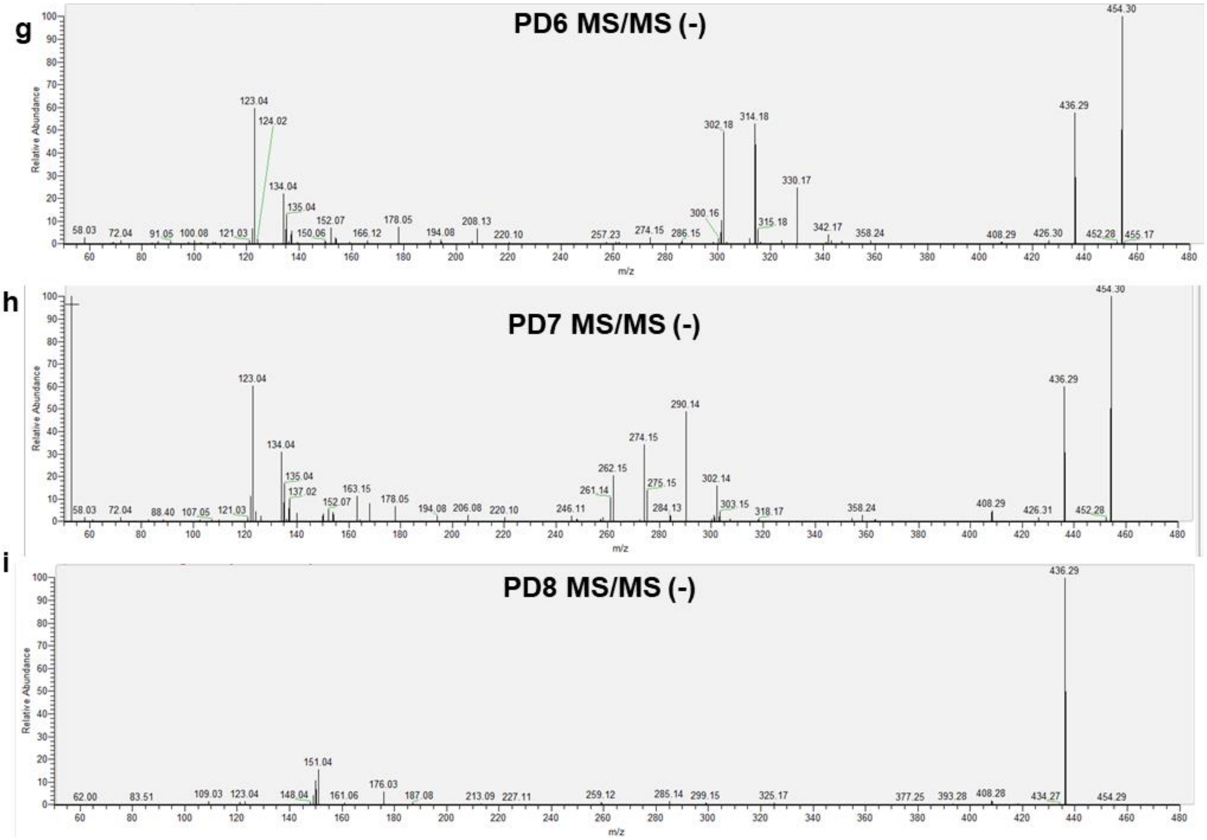

Supplementary Figure 9. *Mono-oxygenation of NADA by CYP2J2*. **(a)** LC-MS/MS chromatogram in negative ion mode. Mass range is ± 5 ppm of the predicted product. **(b-i)** MS/MS spectra obtained from each corresponding product.

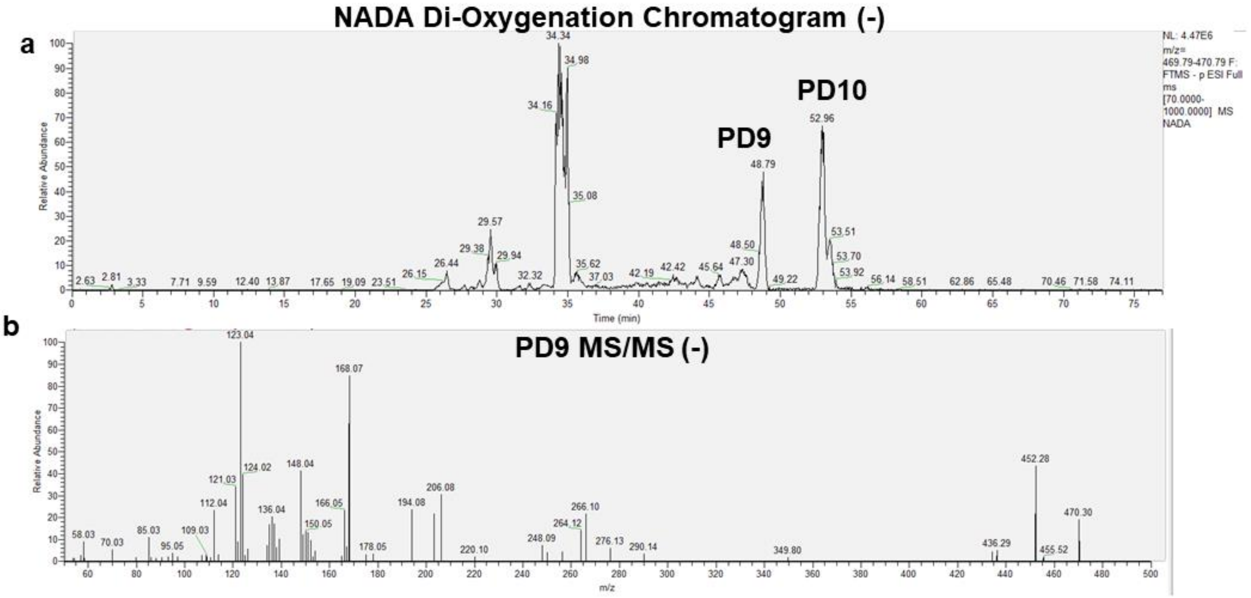

Supplementary Figure 10. *Di-oxygenation of NADA by CYP2J2*. **(a)** LC-MS/MS chromatogram in negative ion mode. Mass range is ± 5 ppm of the predicted product. **(b)** MS/MS spectrum obtained from PD9.

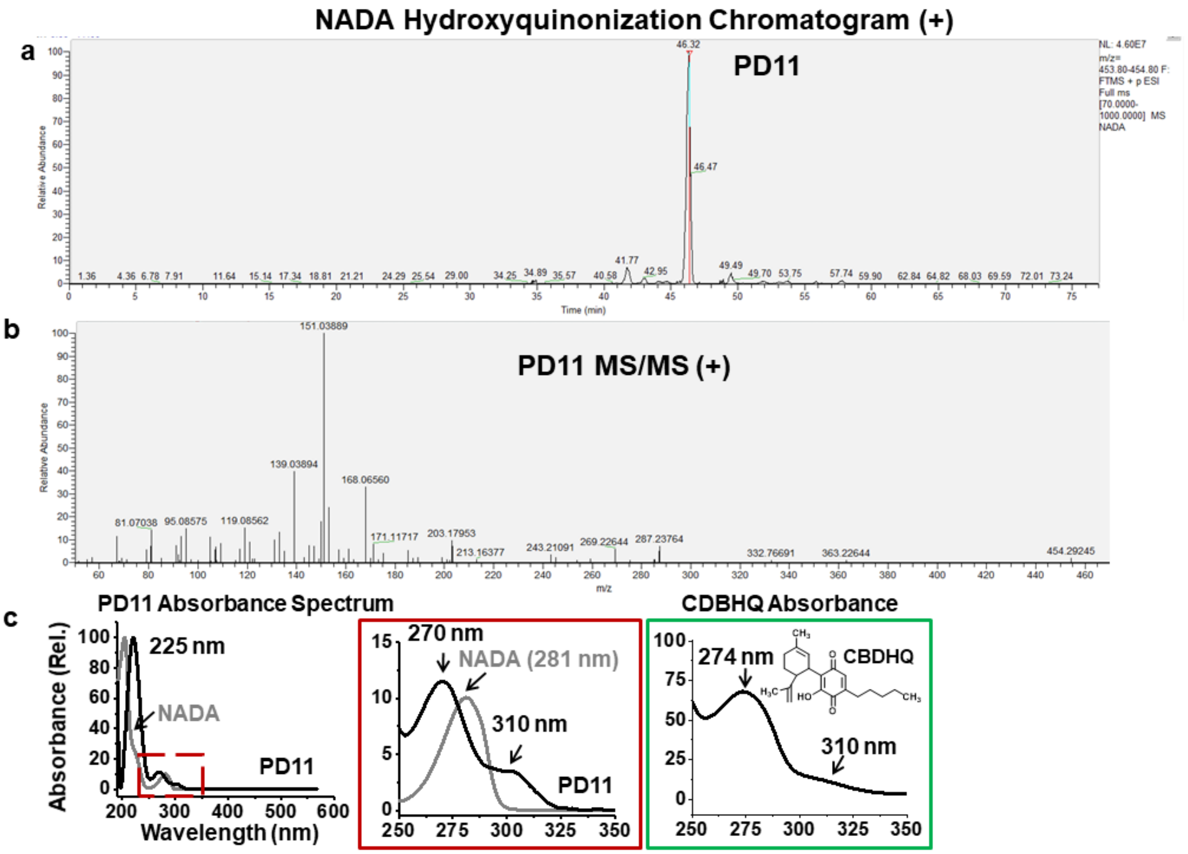

Supplementary Figure 11. *Hydroxyquinonization of NADA by CYP2J2*. **(a)** LC-MS/MS chromatogram in positive ion mode. Mass range is ± 6 ppm of the predicted product. **(b)** MS/MS spectrum of PD11. **(c)** Absorbance spectrum of PD11 obtained by UV-Vis HPLC analysis. NADA and cannabidiol hydroxyquinone (CBDHQ) were analyzed using a similar method and their absorbance spectra are given for comparison. It is known that dopamine is oxidized to form 6-hydroxy-*p*-quinone,[16,17] and therefore, NADA-6-hydroxy-*p*-quinone is likely the identity of PD11.

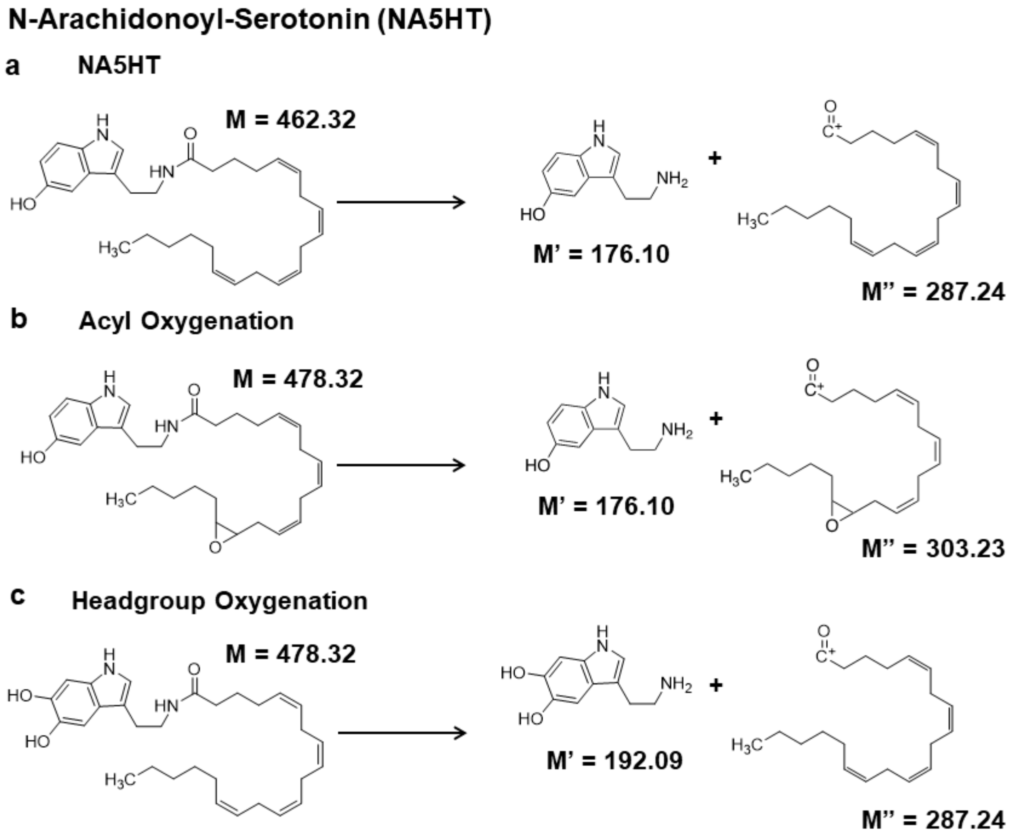

Supplementary Figure 12. Fragmentation scheme of **(a)** NA5HT, **(b)** acyl oxygenation of NA5HT (14',15'-epoNA5HT shown as an example), and **(c)** Headgroup oxygenation of NA5HT (structure of headgroup oxidation shown as an example and is not confirmed).

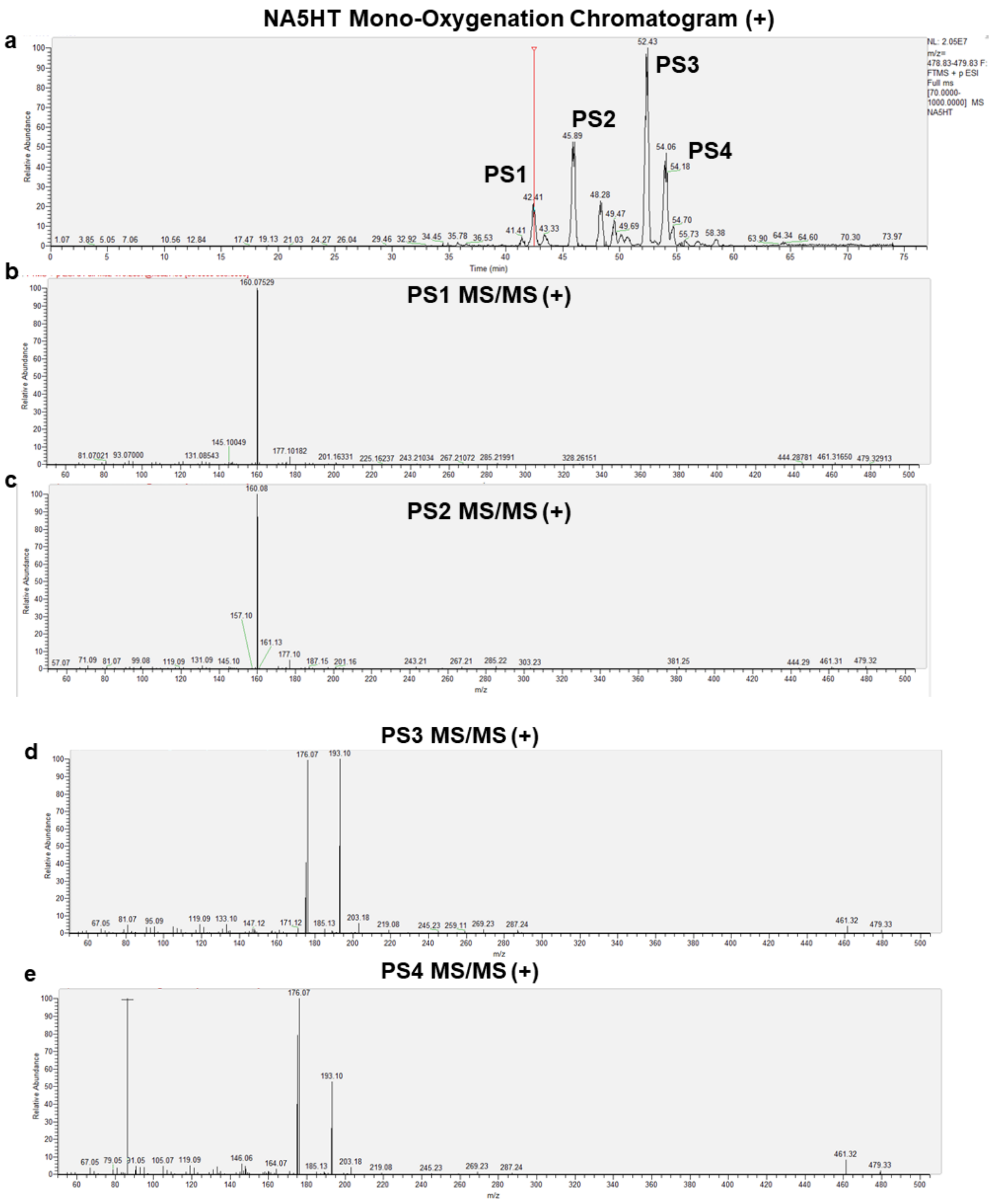

Supplementary Figure 13. *Mono-oxygenation of NA5HT by CYP2J2*. **(a)** LC-MS/MS chromatogram in positive ion mode. Mass range is ± 5 ppm of the predicted product. **(b-e)** MS/MS spectra obtained from each corresponding product.

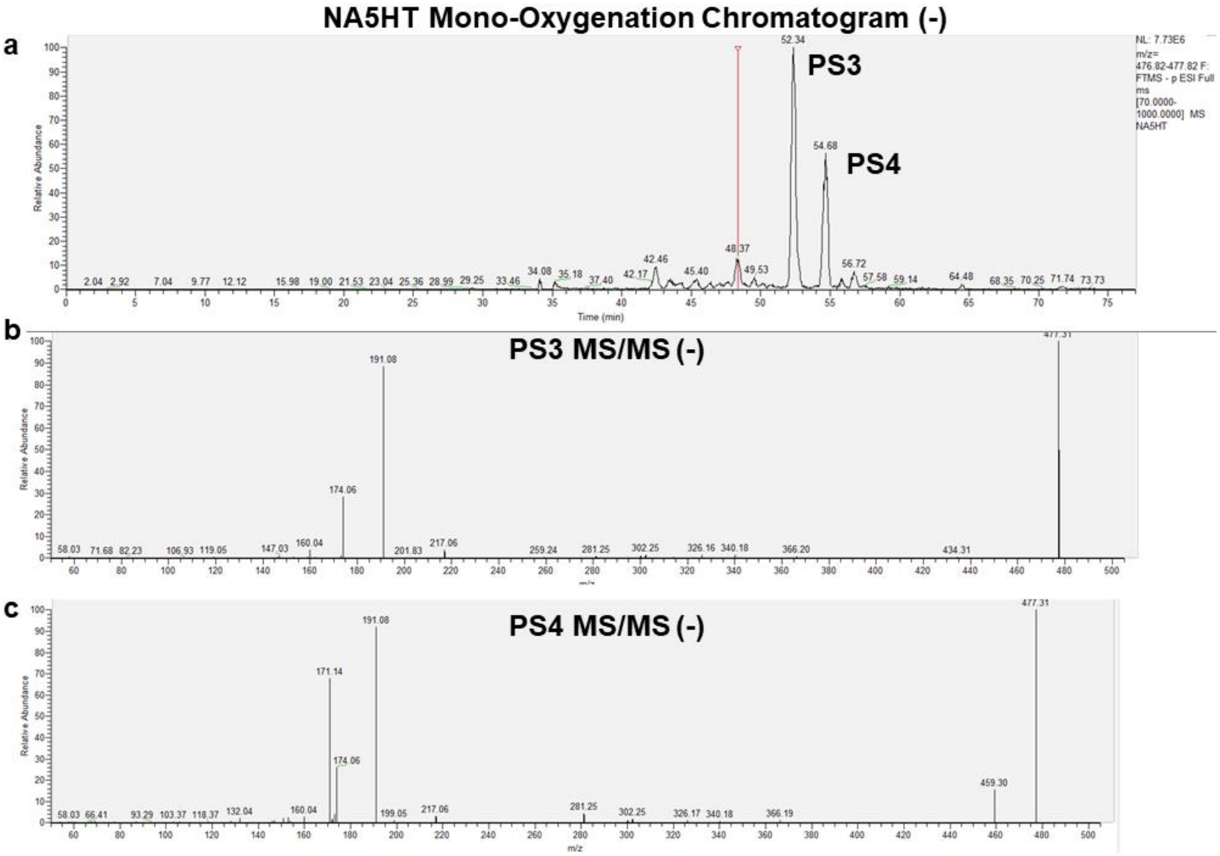

Supplementary Figure 14. *Mono-oxygenation of NA5HT by CYP2J2*. **(a)** LC-MS/MS chromatogram in negative ion mode. Mass range is ± 5 ppm of the predicted product. **(b-c)** MS/MS spectra obtained from each corresponding product.

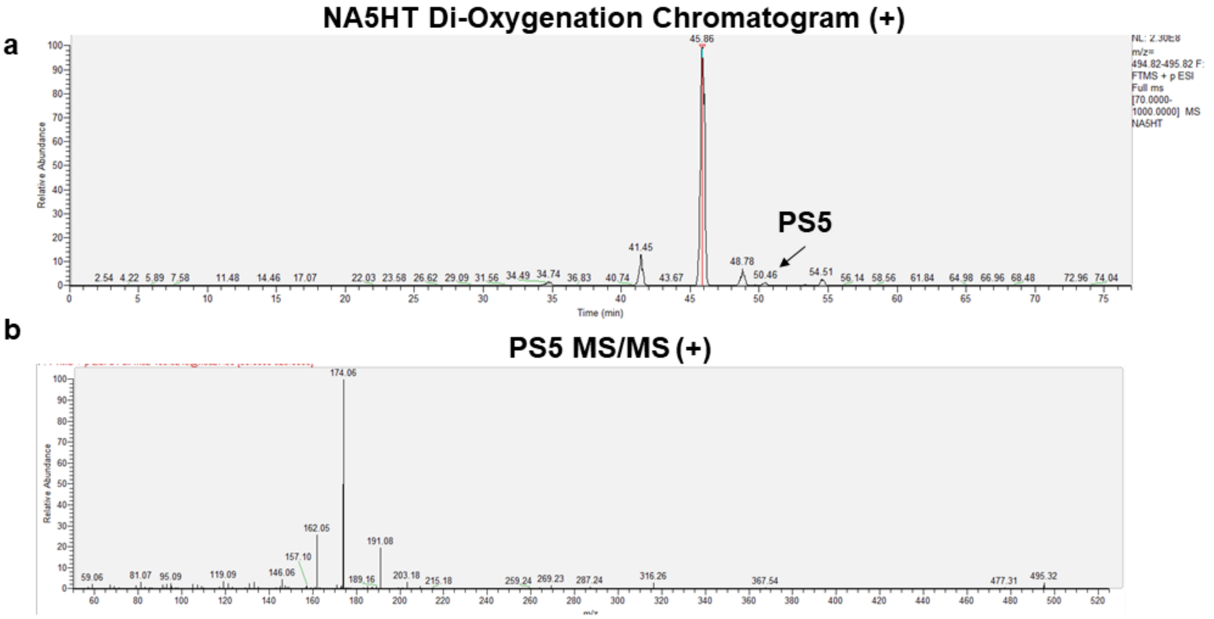

Supplementary Figure 15. *Di-oxygenation of NA5HT by CYP2J2*. **(a)** LC-MS/MS chromatogram in positive ion mode. Mass range is ± 5 ppm of the predicted product. **(b)** MS/MS spectrum of PS5.

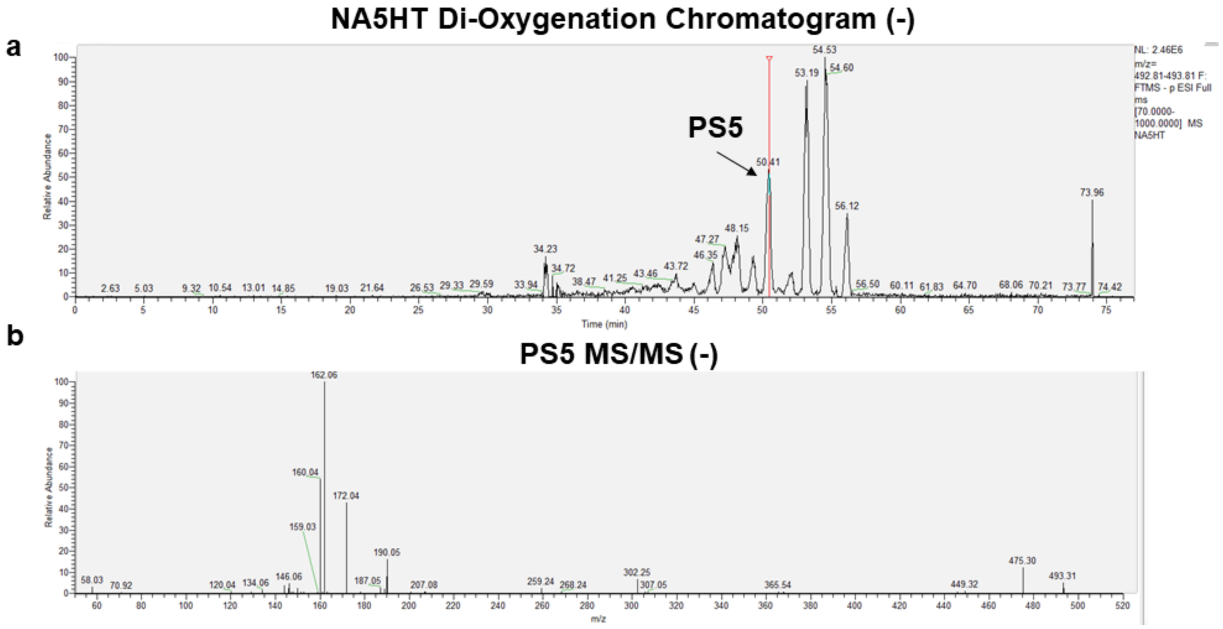

Supplementary Figure 16. *Di-oxygenation of NA5HT by CYP2J2*. **(a)** LC-MS/MS chromatogram in negative ion mode. Mass range is ± 5 ppm of the predicted product. **(b)** MS/MS spectrum of PS5.

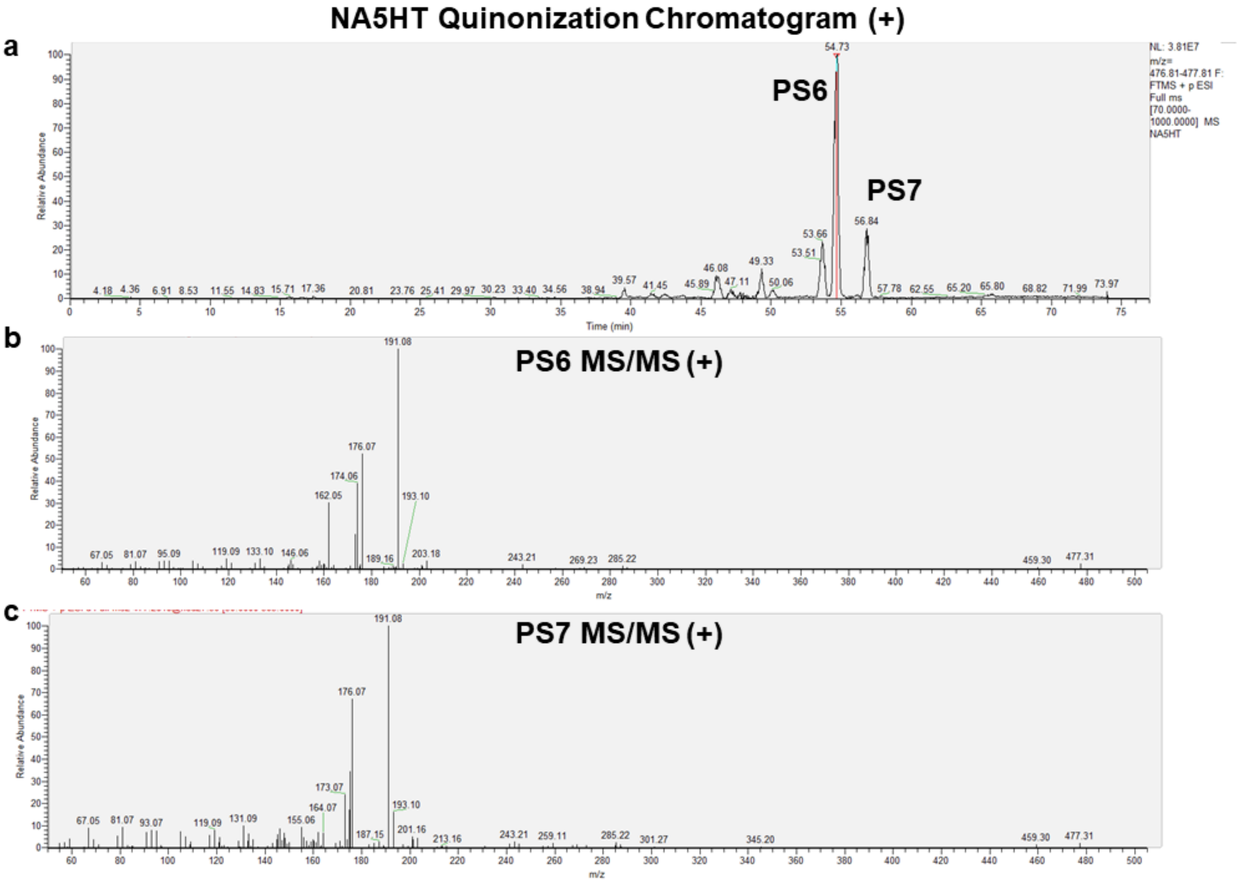

Supplementary Figure 17. *Quinonization of NA5HT by CYP2J2*. Quinonization was determined from the apparent oxygenation of the mono-oxygenation products (PS3 and PS4) indicated by a loss of 2 hydrogen atoms. **(a)** LC-MS/MS chromatogram in positive ion mode. Mass range is ± 5 ppm of the predicted product. **(b-c)** MS/MS spectra obtained from the indicated product.

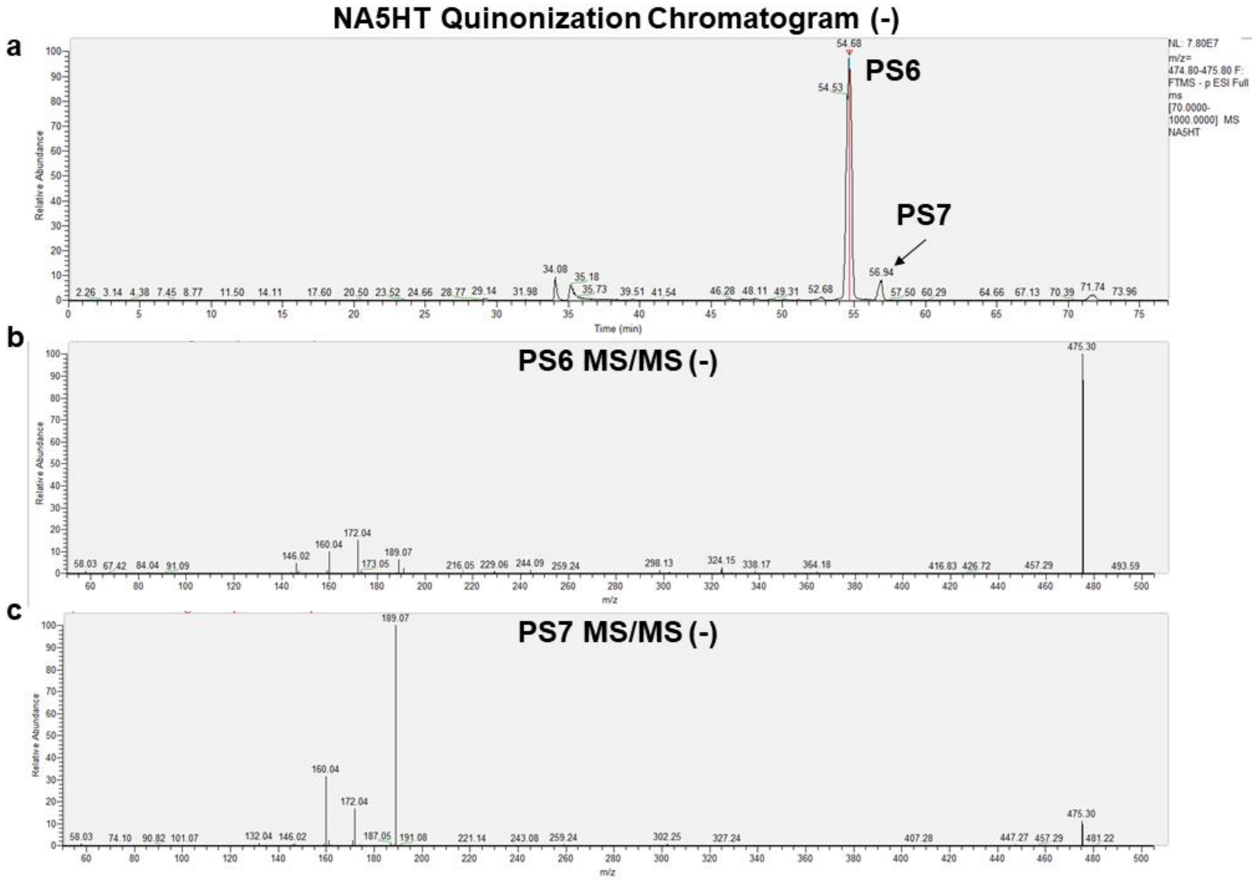

Supplementary Figure 18. *Quinonization of NA5HT by CYP2J2*. Quinonization was determined from the apparent oxygenation of the mono-oxygenation products (PS3 and PS4) indicated by a loss of 2 hydrogen atoms. **(a)** LC-MS/MS chromatogram in negative ion mode. Mass range is ± 5 ppm of the predicted product. **(b-c)** MS/MS spectra obtained from the indicated product.

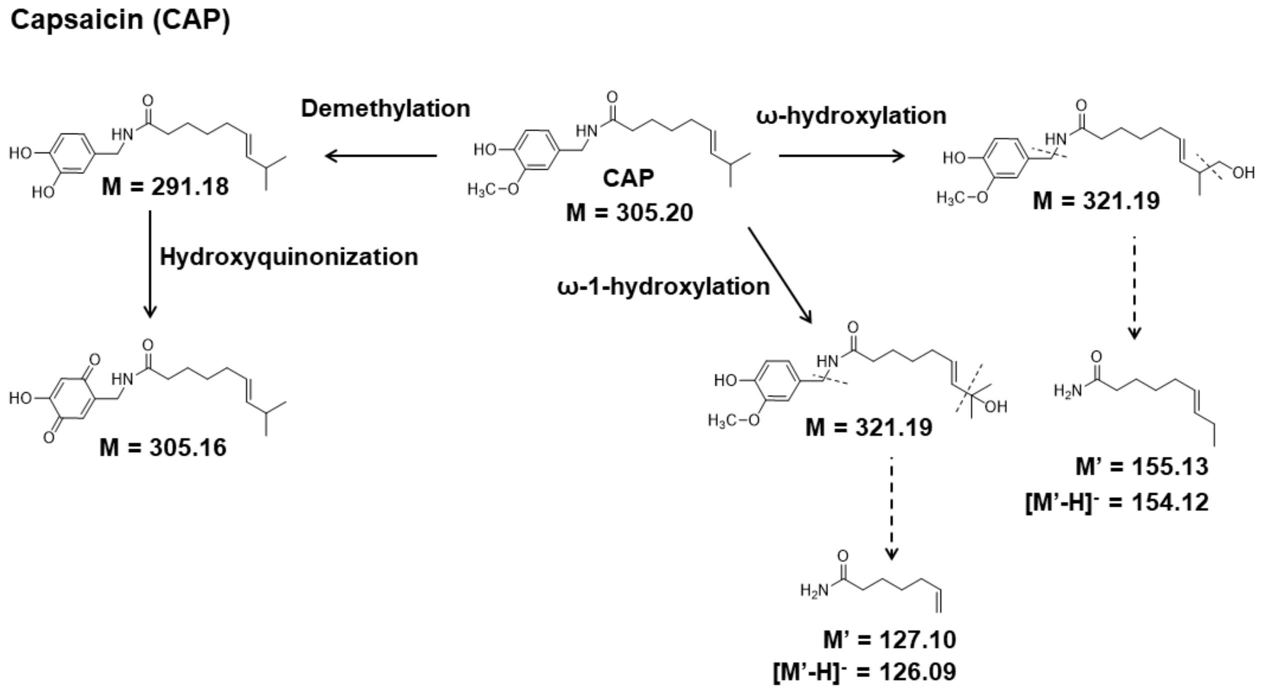

Supplementary Figure 19. Fragmentation scheme of capsaicin oxygenation.

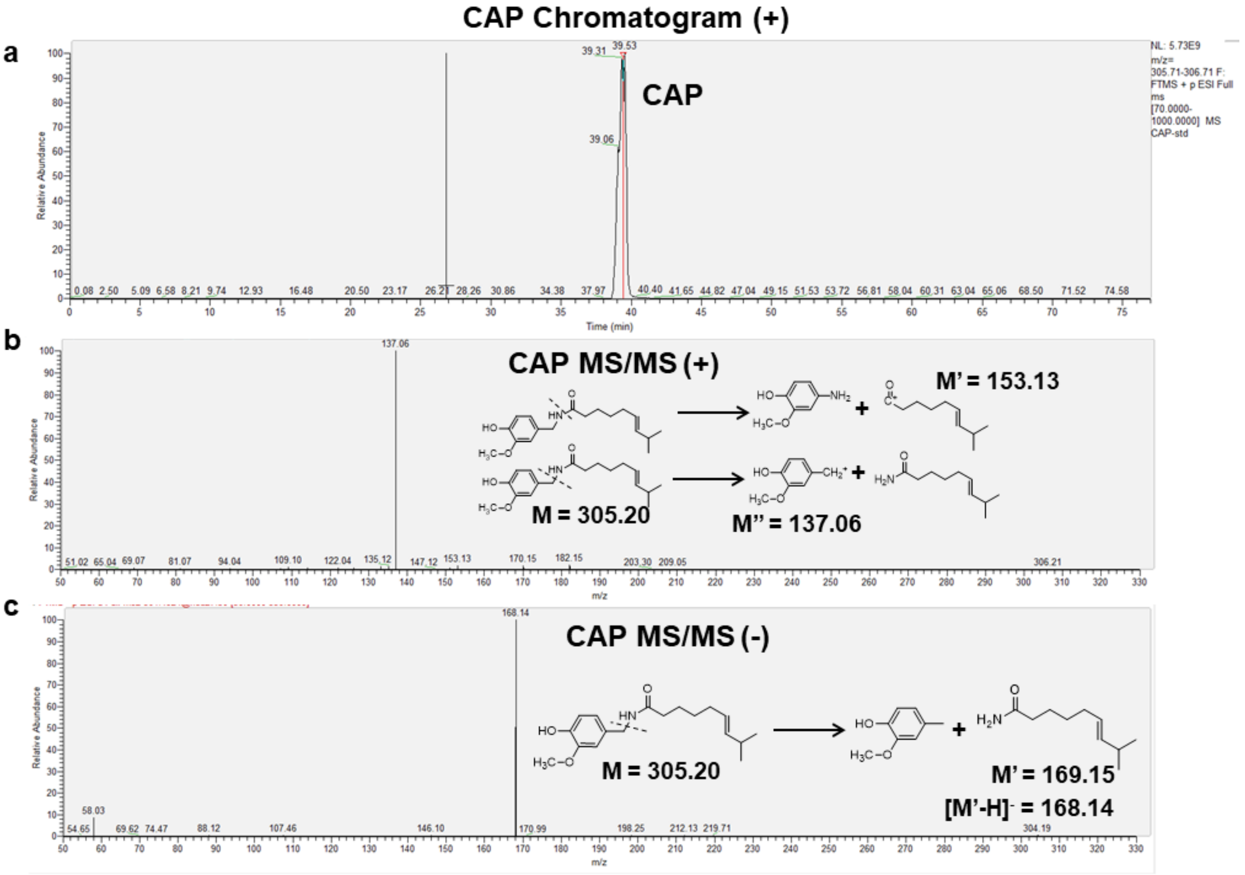

Supplementary Figure 20. *LC-MS/MS analysis of capsaicin (CAP).* **(a)** LC-MS/MS chromatogram in positive ion mode. Mass range is ± 5 ppm of the predicted product. **(b)** MS/MS spectrum of CAP in positive ion mode. **(c)** MS/MS spectrum of CAP in negative ion mode.

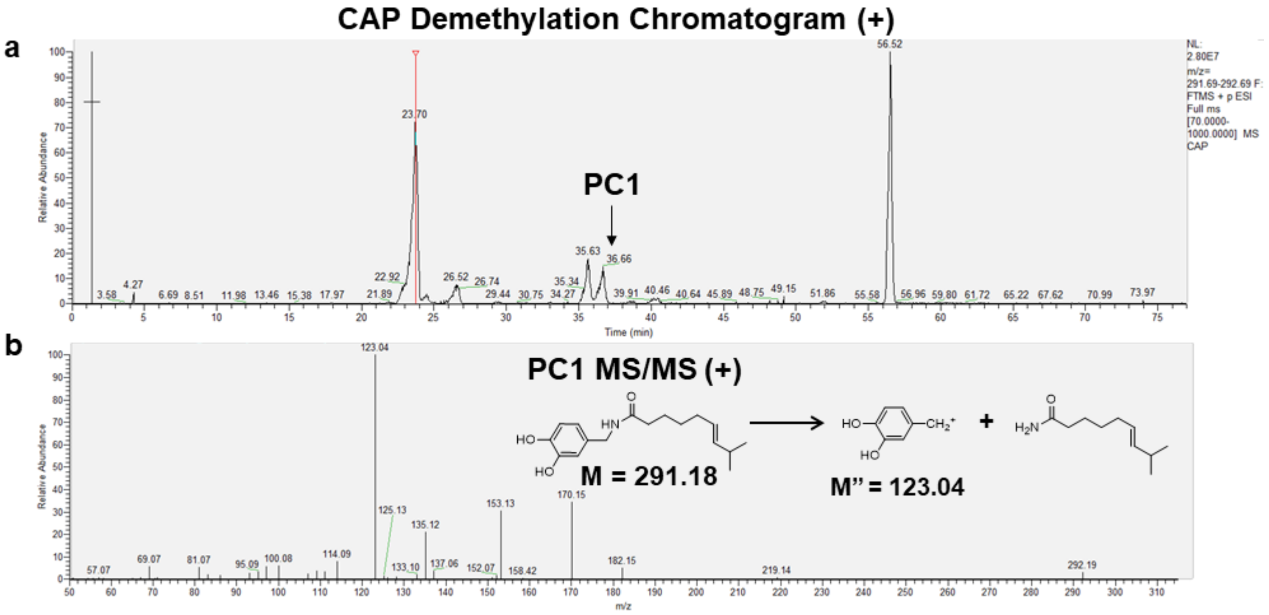

Supplementary Figure 21. *Demethylation of CAP by CYP2J2*. **(a)** LC-MS/MS chromatogram in positive ion mode. Mass range is ± 5 ppm of the predicted product. **(b)** MS/MS spectrum of PC1.

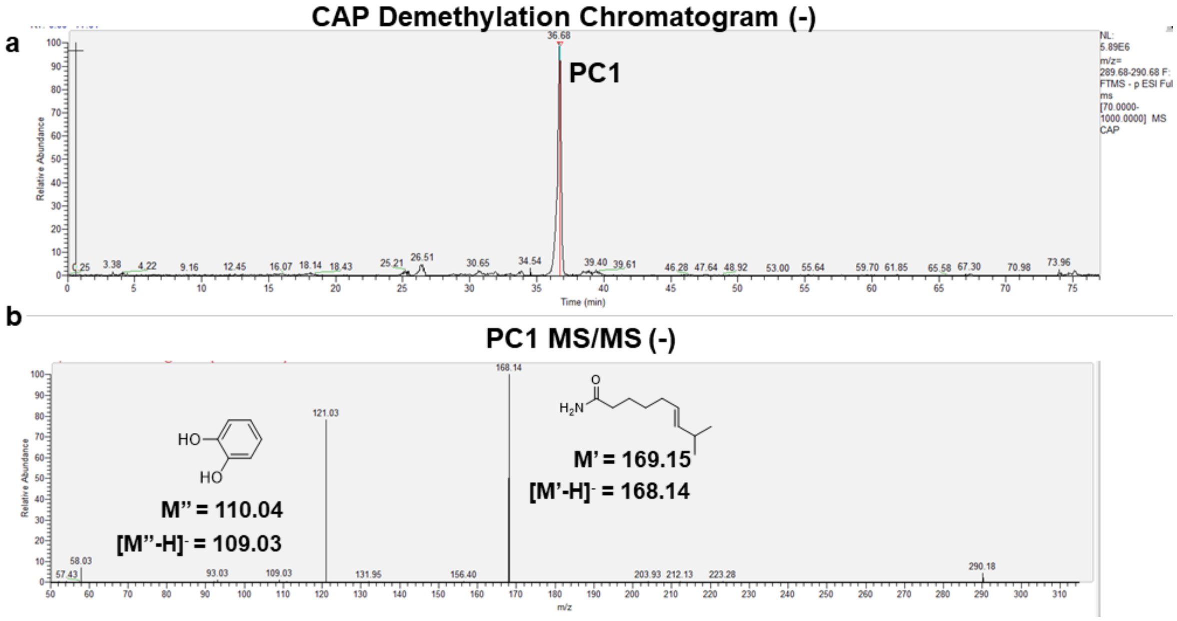

Supplementary Figure 22. *Demethylation of CAP by CYP2J2*. **(a)** LC-MS/MS chromatogram in negative ion mode. Mass range is ± 5 ppm of the predicted product. **(b)** MS/MS spectrum of PC1.

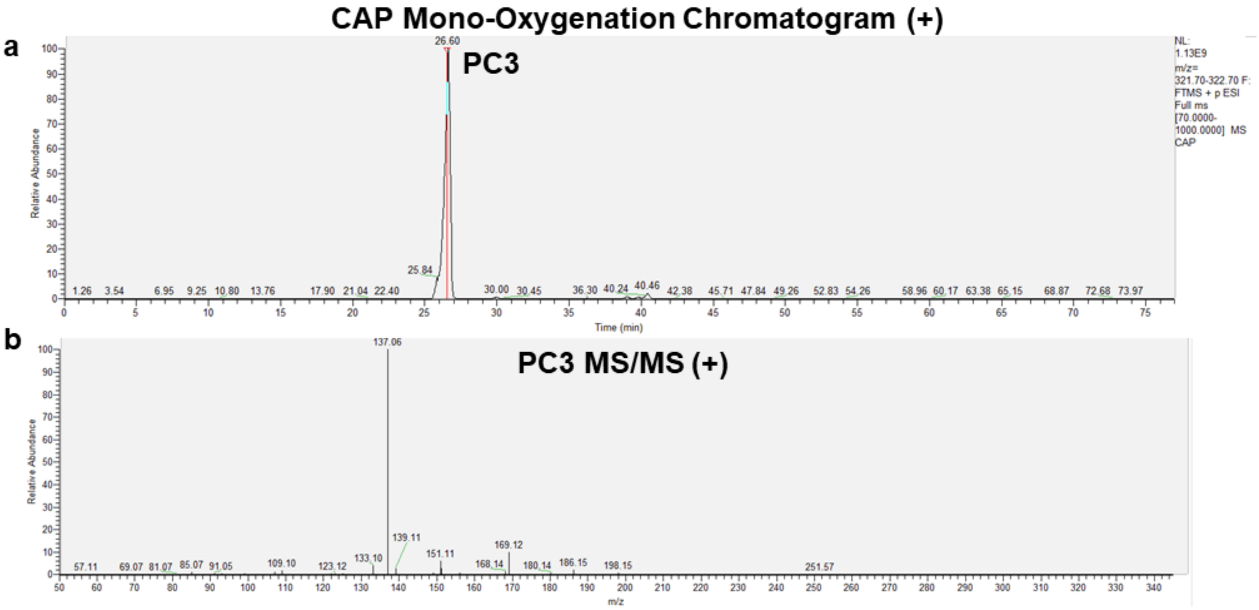

Supplementary Figure 23. *Mono-oxygenation of CAP by CYP2J2*. **(a)** LC-MS/MS chromatogram in positive ion mode. Mass range is ± 5 ppm of the predicted product. **(b)** MS/MS spectrum of PC3.

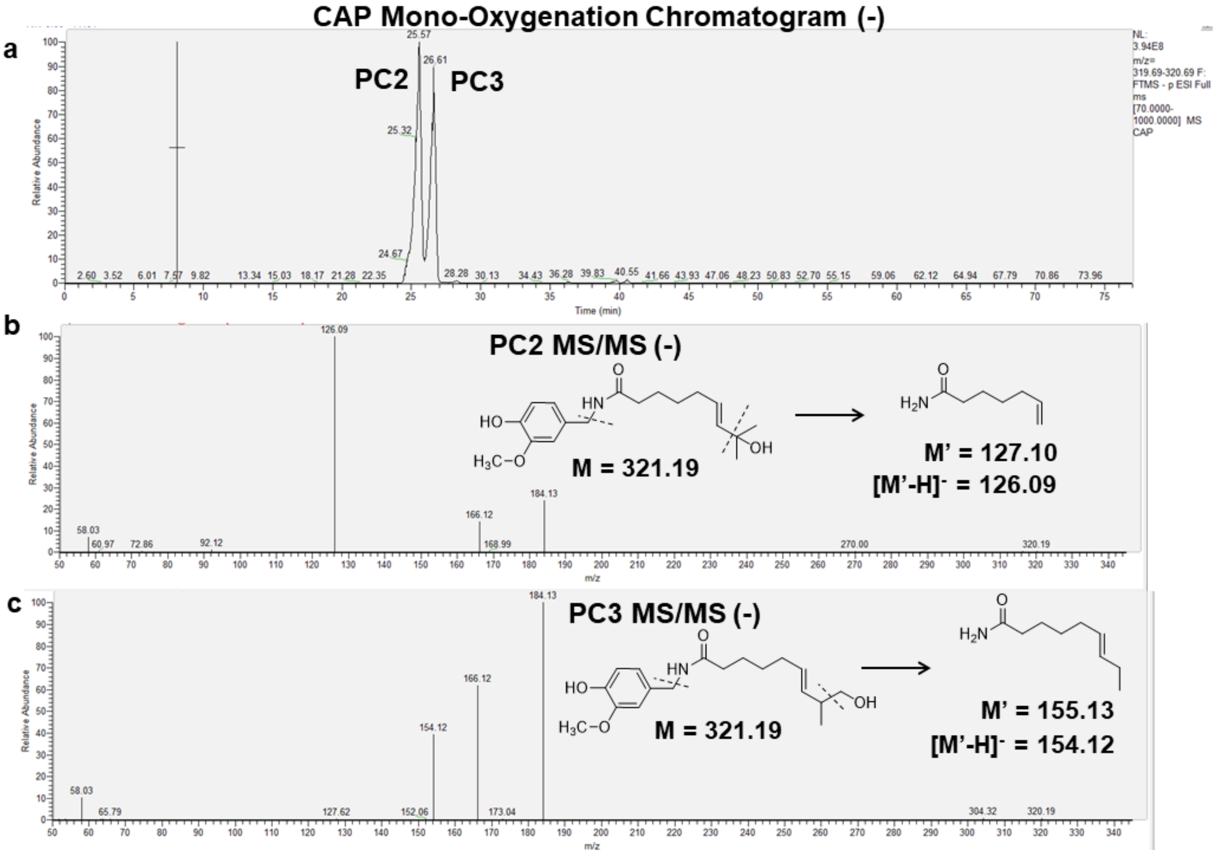

Supplementary Figure 24. *Mono-oxygenation of CAP by CYP2J2*. **(a)** LC-MS/MS chromatogram in negative ion mode. Mass range is ± 5 ppm of the predicted product. **(b-c)** MS/MS spectra of indicated products.

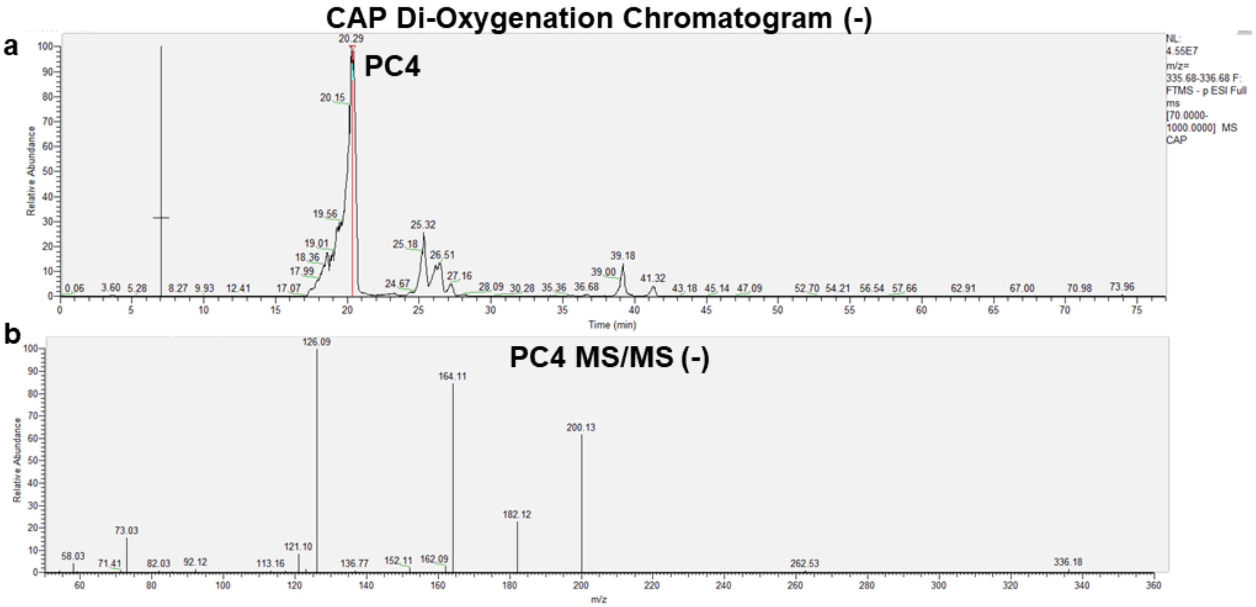

Supplementary Figure 25. *Di-oxygenation of CAP by CYP2J2*. **(a)** LC-MS/MS chromatogram in negative ion mode. Mass range is ± 5 ppm of the predicted product. **(b)** MS/MS spectrum of PC4.

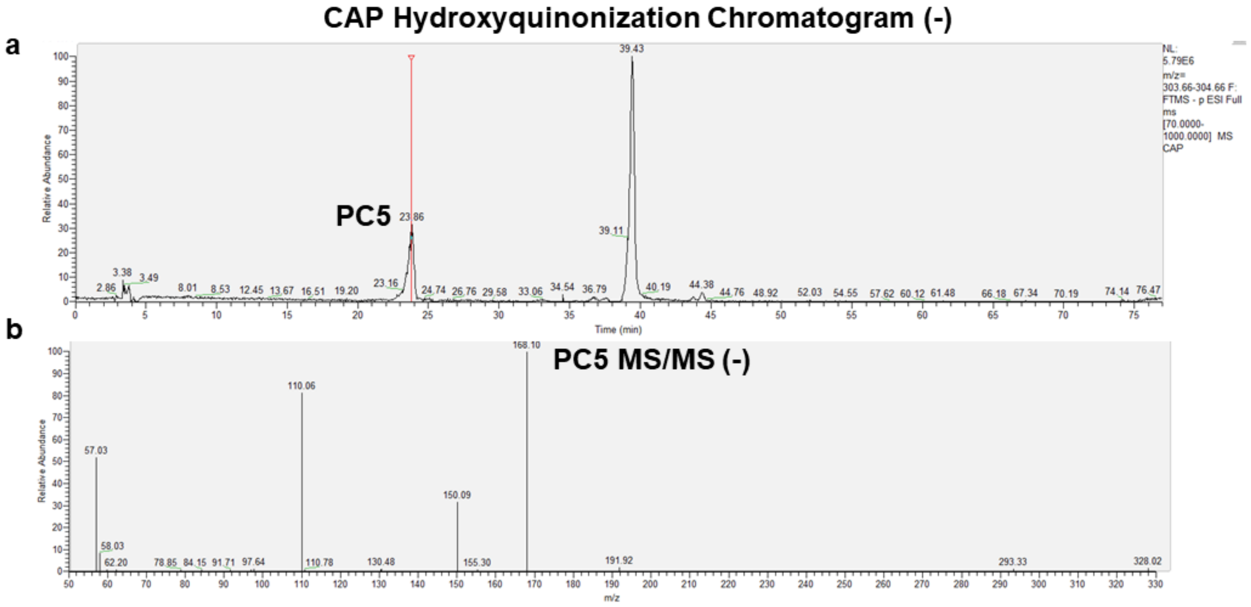

Supplementary Figure 26. *Hydroxyquinonization of CAP by CYP2J2*. Hydroxyquinonization is determined by an oxidation of the demethylated CAP (PC1) indicated by the loss of two hydrogen atoms. **(a)** LC-MS/MS chromatogram in negative ion mode. Mass range is ± 5 ppm of the predicted product. **(b)** MS/MS spectrum of PC5.

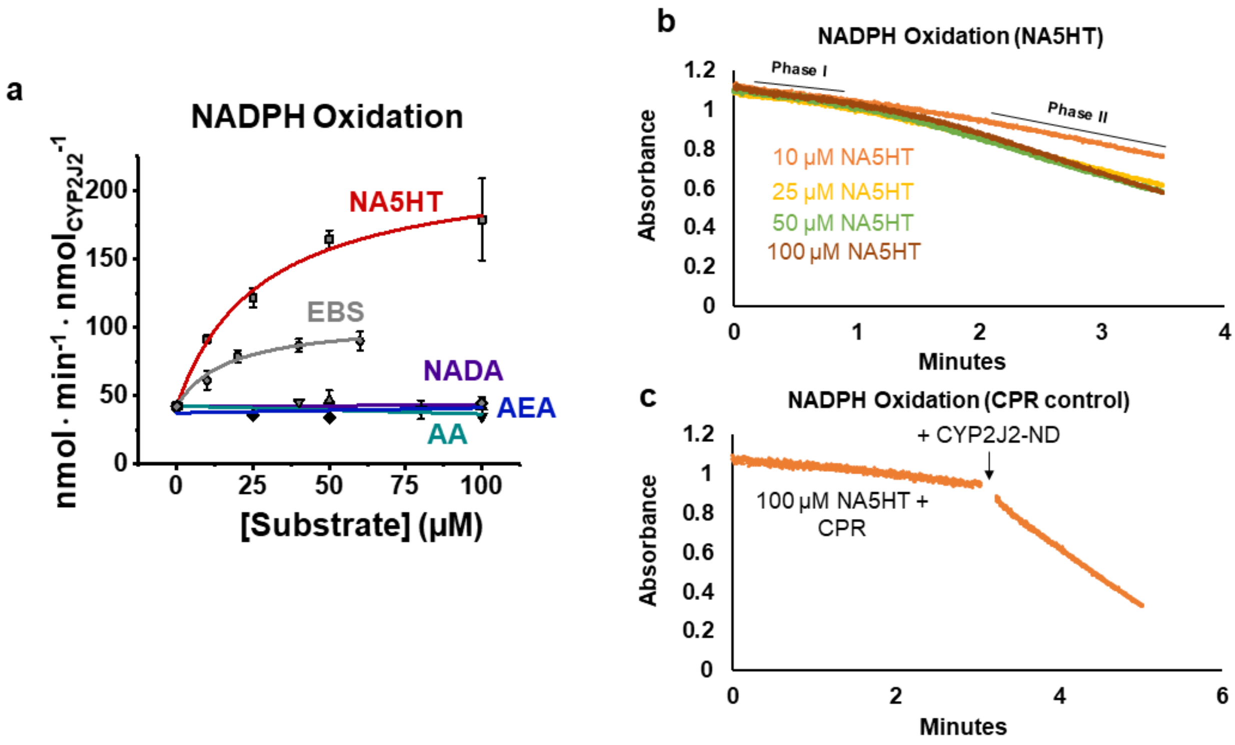

Supplementary Figure 27. *Biphasic NADPH oxidation in the presence of NA5HT*. NADPH oxidation as CYP2J2-ND metabolizes NA5HT was determined by the absorbance of NADPH at 340 nm. Data represents the SEM of three independent experiments. Representative spectra are shown. **(a)** NADPH oxidation rates with the indicated substrates. NA5HT shows biphasic NADPH oxidation kinetics. Phase I for all concentrations matches baseline rates (CPR and CYP2J2 without NA5HT). The Rates of Phase II are plotted in the figure. The K_m and V_{max} of Phase II are 27.3 ± 11.3 μM and 178 ± 28 $nmol_{NADPH} \cdot min^{-1} \cdot nmol_{CYP2J2}^{-1}$, respectively. Ebastine (EBS) is monophasic and has kinetics parameters of K_m = 18.9 ± 9.5 μM and V_{max} = 64.8 ± 12.1 $nmol_{NADPH} \cdot min^{-1} \cdot nmol_{CYP2J2}^{-1}$. **(b)** Absorbance at 340 nm in the presence of the indicated amounts of NA5HT. Phase I rates are similar to those obtained with CPR and CYP2J2-ND alone. Phase II shows a decrease in NADPH as a function of NA5HT concentration. **(c)** Experiments were repeated with NA5HT and CPR only. Rate of NADPH oxidation is similar to CPR alone. Adding CYP2J2-ND results in a rate of NADPH oxidation similar to the Phase II of 100 μM NA5HT as seen in **(b)**. Data can be found in the Source Data file.

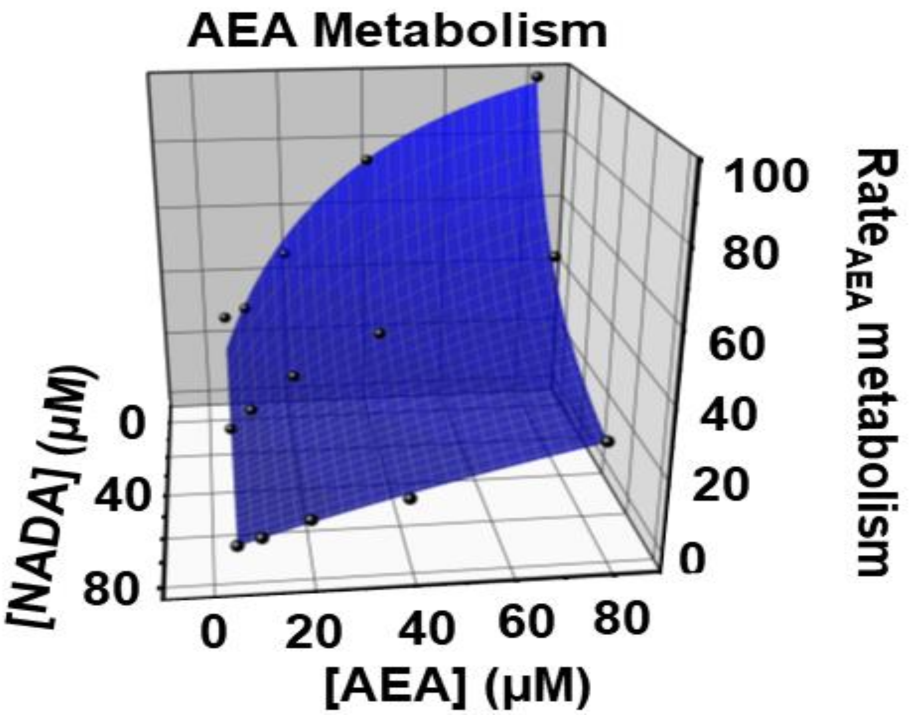

Supplementary Figure 28. 3D global fit of AEA inhibition by NADA as shown in Figure 3f. Rates are in $pmol_{EET\text{-}EAs} \cdot min^{-1} \cdot nmol_{CYP2J2}^{-1}$. Data can be found in the Source Data file.

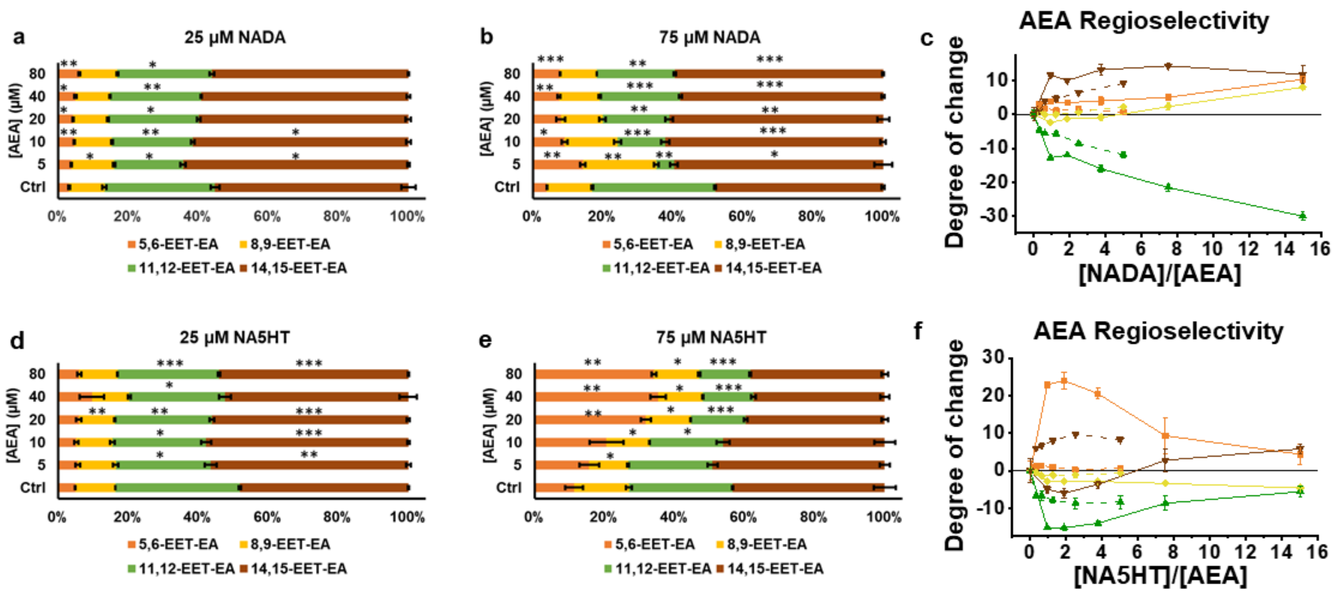

g

25 µM NADA	5,6-EET-EA	8,9-EET-EA	11,12-EET-EA	14,15-EET-EA
5 µM AEA	0.1144385	0.0315352	0.0030012	0.01018093
10 µM AEA	0.0091623	0.2289844	0.0049989	0.03540108
20 µM AEA	0.017267	0.913827	0.01640164	0.086447
40 µM AEA	0.0273624	0.8548984	0.00953489	0.0944611
80 µM AEA	0.0054711	0.2338745	0.03553634	0.57759347

75 µM NADA	5,6-EET-EA	8,9-EET-EA	11,12-EET-EA	14,15-EET-EA
5 µM AEA	0.0017096	0.0026845	0.000282144	0.03857806
10 µM AEA	0.0227428	0.1854282	0.000678421	0.00069429
20 µM AEA	0.0909166	0.4886497	0.001395266	0.00996682
40 µM AEA	0.009347	0.0631254	0.000219691	0.00079405
80 µM AEA	5.99E-05	0.0010161	8.51395E-05	0.00021154

25 µM NA5HT	5,6-EET-EA	8,9-EET-EA	11,12-EET-EA	14,15-EET-EA
5 µM AEA	0.4960847	0.6258162	0.03303457	0.00203935
10 µM AEA	0.7713618	0.2693141	0.01899977	0.00014094
20 µM AEA	0.3668372	0.0099252	0.00337816	0.00064675
40 µM AEA	0.3687003	0.2733748	0.03283166	0.27472115
80 µM AEA	0.2037727	0.0157322	0.00075801	0.00024499

75 µM NA5HT	5,6-EET-EA	8,9-EET-EA	11,12-EET-EA	14,15-EET-EA
5 µM AEA	0.36014316	0.006011316	0.060703962	0.148848765
10 µM AEA	0.250497516	0.011299366	0.038552102	0.578014951
20 µM AEA	0.004832614	0.017464704	0.000359393	0.263965653
40 µM AEA	0.006106328	0.023810142	0.000408967	0.136193174
80 µM AEA	0.001728063	0.032159009	2.55709E-05	0.157941302

Supplementary Figure 29. *Regioselectivity of AEA epoxidation in the presence of eVDs.* **(a-c)** Regioselectivity in the presence of NADA and **(d-f)** NA5HT at the indicated concentrations. Regioselectivity is shown as a percentage of the total EET-EAs. Control (Ctrl) refers to 40 µM AEA samples without eVDs, which was ran along with each experiment. Data in **(c)** and **(f)** show the dependence of the regioselectivity change on the concentrations of the eVDs as a function of an increasing ratio of the eVD:AEA. Degree of change is defined as the change in percentage value compared to control. Data from the 25 µM datasets are shown as dashed lines and the data from the 75 µM datasets are shown as solid lines. Data represents the mean ± SEM of 3 independent experiments. Statistical significance was determined by a two-tailed t-test with equal variance. *p < 0.05; **p < 0.01, ***p < 0.001. **(g)** Exact p values for the data. Yellow is p < 0.05, orange is p < 0.01, and red is p < 0.001. Data can be found in the Source Data file.

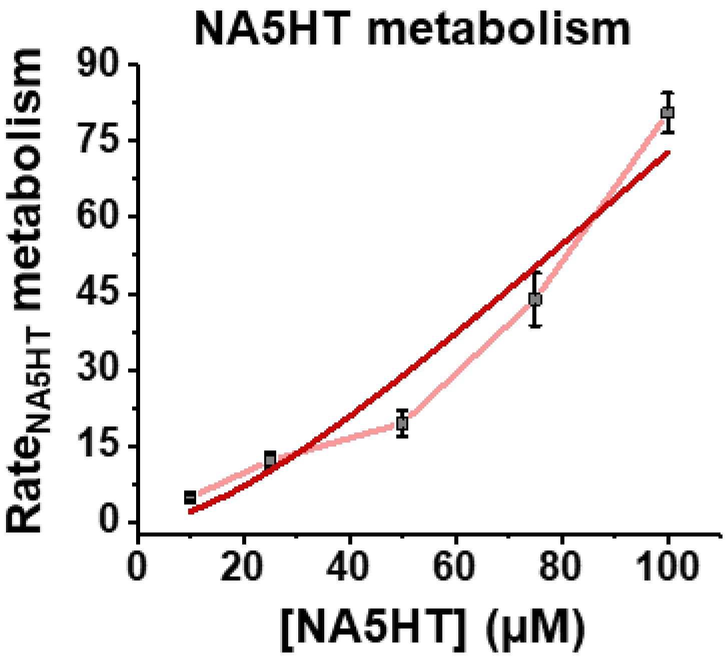

Supplementary Figure 30. NA5HT metabolism data fitted to a two-site (Equation 4) binding equation as detailed in the Results section. Data represents the mean ± SEM of 3 independent experiments. Data can be found in the Source Data file.

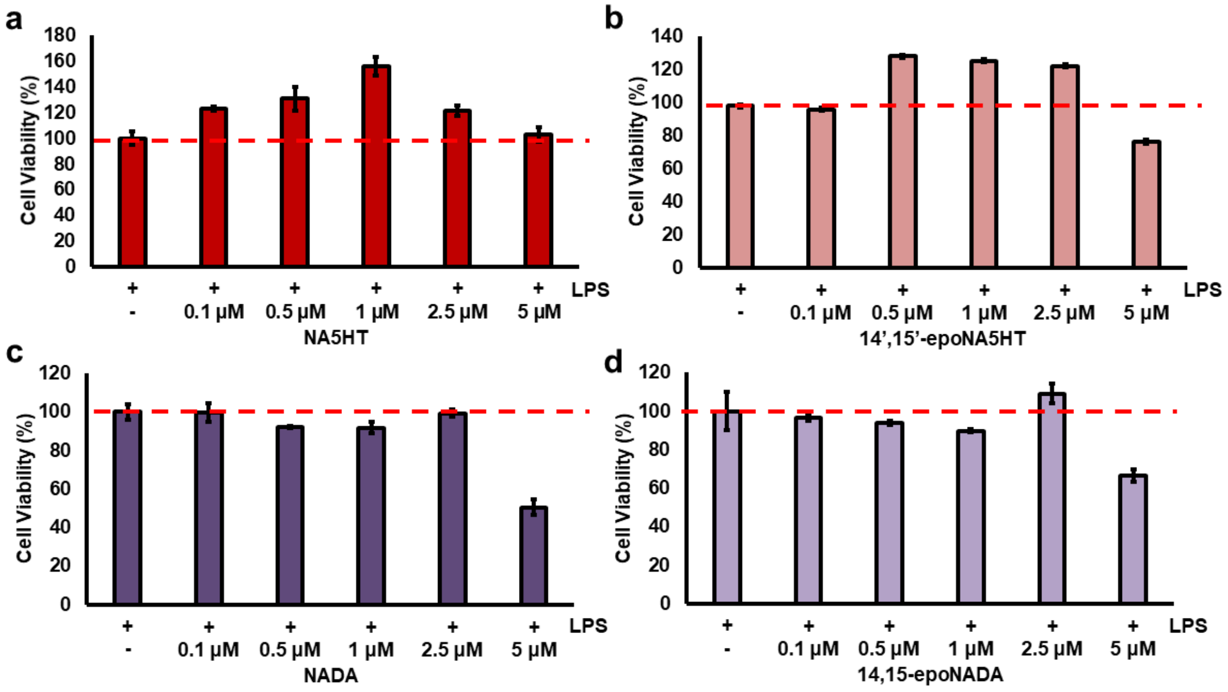

Supplementary Figure 31. *MTT assay to determine cell viability.* BV2 microglial cells seeded at 200,000 cells/well in a 24-well plate were treated with 25 ng/mL LPS for 24 hours and an MTT cell proliferation assay was performed to determine cell viability. Viability was determined as a percent of the formazan absorbance at 570 nm compared to LPS treatment only (100%) in the presence of **(a)** NA5HT, **(b)** 14',15'-epoNA5HT, **(c)** NADA, and **(d)** 14',15'-epoNADA. Data represents the SEM of 3 independent experiments. Data can be found in the Source Data file.

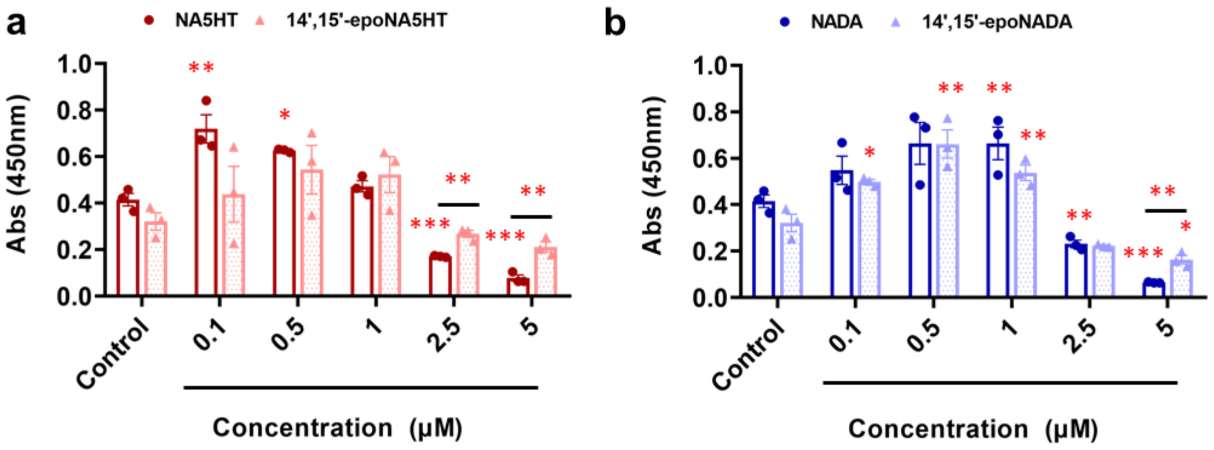

c

t-test	0.1 μM	0.5 μM	1 μM	2.5 μM	5 μM
	**	*		***	***
NA5HT To Control	0.01041	0.00157	0.18828	0.00088	0.00038
EpoNA5HT To Control	0.40062	0.11275	0.0782	0.25767	0.06495
				**	**
NA5HT vs epoNA5HT	0.10535	0.47608	0.56468	0.00341	0.00673

t-test	0.1 μM	0.5 μM	1 μM	2.5 μM	5 μM
			*	**	***
NADA To Control	0.11651	0.05795	0.02974	0.00455	0.00022
	*	**	**		*
EpoNADA To Control	0.01016	0.00871	0.01237	0.05974	0.01944
					**
NADA vs epoNADA	0.46489	0.98165	0.17689	0.6369	0.00611

Supplementary Figure 32. *Effects of eVDS and epoxy-eVDs on cell proliferation.* BV-2 microglial cells were pre-incubated with varying concentrations of **(a)** NA5HT and 14,15-epoNA5HT and **(b)** NADA and 14,15-epoNADA for 4 hours followed by LPS (25 ng/mL) stimulation for 24 hours. After 21 hours, BV-2 cell proliferation was measured using BrdU incorporation colorimetric ELISA assay. Values shown are the mean ± SEM of experiments performed in triplicate ($n = 3$). *p < 0.05, **p < 0.01, and ***p < 0.001. **(c)** Tables for p-values of the data. Cells are highlighted as yellow p < 0.05, orange p < 0.01, and red p < 0.001. Data can be found in the Source Data file.

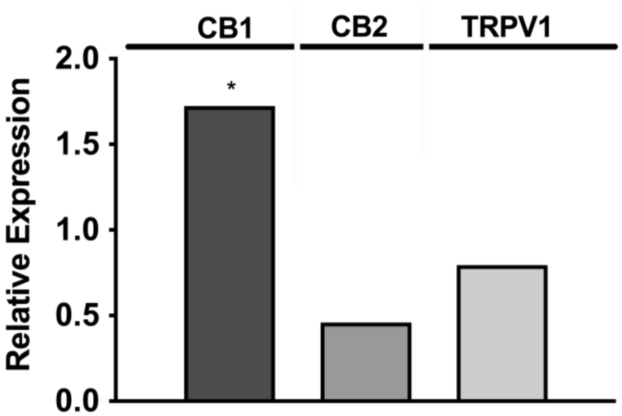

Supplementary Figure 33. *Relative mRNA Expression of CB1, CB2, and TRPV1 in LPS-treated BV2 microglial cells.* BV2 microglial cells seeded at 200,000 cells/well in a 24-well plate were treated with 25 ng/mL LPS for 24 hours. RT-qPCR detected the presence of TRPV1, CB1, and CB2 in BV2 cells. Data is from 3-pooled wells and is performed in technical replicates. Data is reported as the mean relative expression. Statistical analysis was performed using a two-tailed t-test ($\alpha = 0.05$). P-values: 0.0332 (*), 0.0021(**), 0.0002 (***), and < 0.0001 (****). Data can be found in the Source Data file.

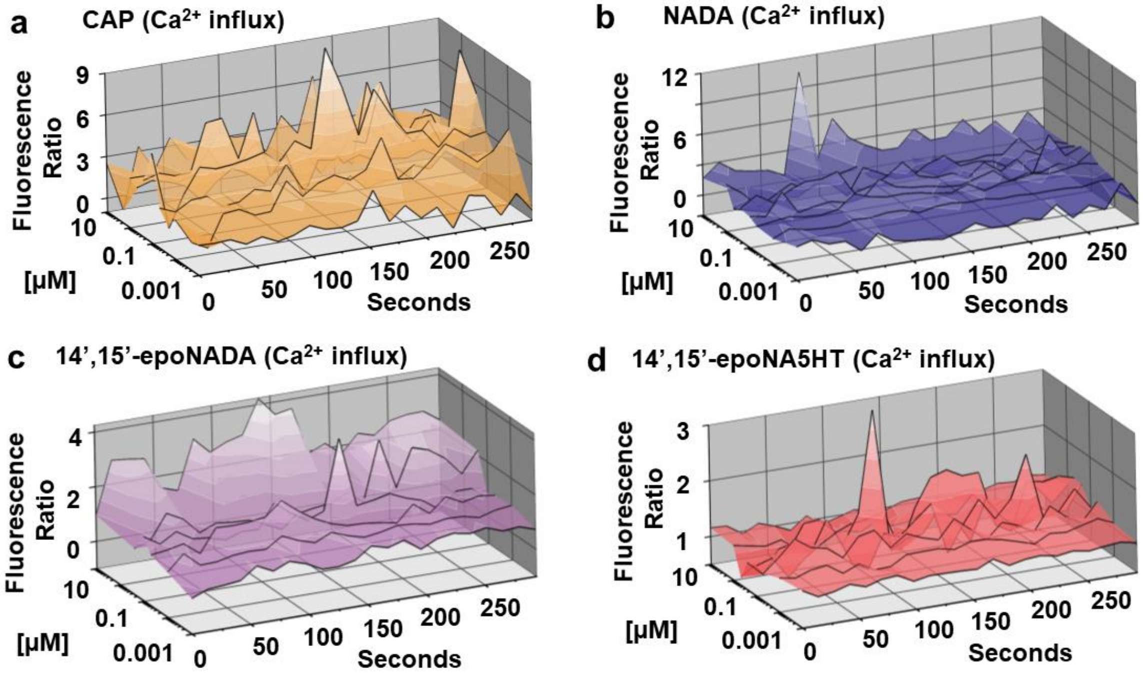

Supplementary Figure 34. *TRPV1 activation by vanilloids.* Relative Ca^{2+} influx upon TRPV1 activation was measured using a Fura-2 AM fluorescence assay. The fluorescence intensity at 510 nm from the Ca^{2+}-bound (excitation 340 nm) and Ca^{2+}-free (excitation 380 nm) dye was measured over time at varying concentrations of ligands and are reported as a ratio (Fluorescence Ratio). Representative plots of **(a)** Capsaicin (CAP), **(b)** NADA, **(c)** 14',15'-epoNADA, and **(d)** 14',15'-epoNA5HT from 4-6 separate experiments (2 sets of triplicate on separate days) are shown. Data can be found in the Source Data file.

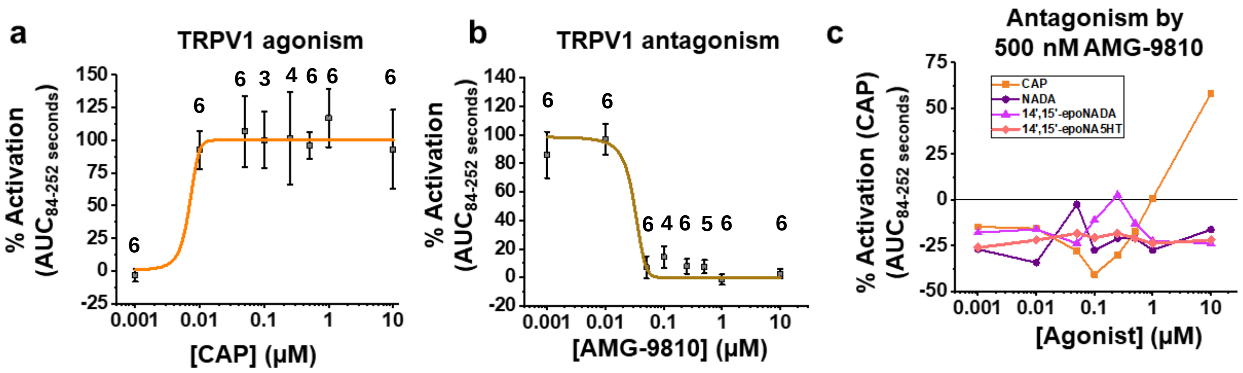

Supplementary Figure 35. *TRPV1-mediated Ca^{2+} influx agonized by CAP and antagonized by AMG-9810.* A Fura 2-AM fluorescence assay was used to determine TRPV1 binding in transfected HEK cells. 100% is defined as the B_{max} of CAP binding. **(a)** CAP agonism data. **(b)** Antagonism of TRPV1 by AMG-9810. 250 nM of CAP was used to activate TRPV1. Data in **(a-b)** is shown as the SEM of 4-6 experiments (2 sets of triplicate performed on separate days) and numbers above the data points represent the exact value of *n*. **(c)** HEK-TRPV1 cells were treated with 500 nM of reversible antagonist AMG-9810 30 min prior to stimulating with the indicated agonists. AMG-9810 prevented agonism of TRPV1 for all compounds. Data represents the SEM of 3 independent experiments. CAP was found to have an EC_{50} of 6.88 ± 3.36 nM, in accordance with reported values[18]. Data can be found in the Source Data file.

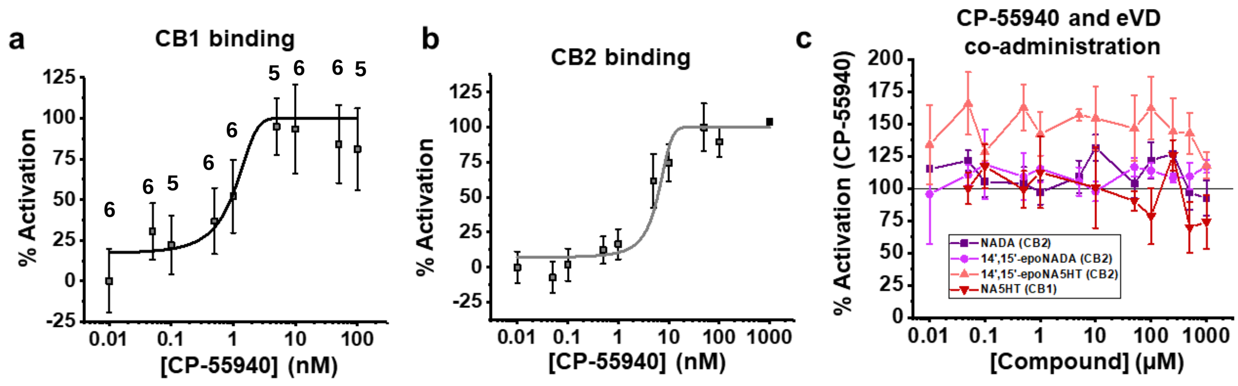

Supplementary Figure 36. *PRESTO-Tango assays of CB1 and CB2 activation.* **(a)** Agonism of CB1. Data respresents the SEM of 3-6 separate experiments (2 sets of triplicates from separate days) Numbers above data points represent the exact value of *n*. **(b)** Agonism of CB2 by CP-55940. Data represents the SEM of 3 independent experiments. **(c)** Co-administration of eVDs and CP-55940 to determine eVD binding to CB1 and 2. CB1 and CB2 were stimulated with 50 nM of CP-55940 to determine antagonism by NADA (CB2), 14',15'-epoNADA (CB2), 14',15'-epoNA5HT (CB2), and NA5HT (CB1). 100% is defined as the B_{max} of CP-55940 binding. Data represents the SEM of 3 independent experiments. EC_{50} values for CP55940 were 0.91 ± 0.38 nM and 5.2 ± 1.9 nM at CB1 and CB2, respectively and were similar to previously reported values [3,19,20]. Data can be found in the Source Data file.

Tables

Supplementary Table 1. Primers for Quantitative Polymerase Chain Reaction

Gene	Forward Primer (5’ → 3’)	Reverse Primer (5’ → 3’)	Reference
Cyp2j9	AACCGTTTCCCCAGTCAGTC	ATTGCACGCACTCTCTGTCA	[21]
Cyp2j12	ATGGAAGACAGCCAAGCACA	AGCCAGTGTGCTATCAGCTG	(designed in-house)
Il-6	CCAGAGATACAAAGAAATGATGG	ACTCCAGAAGACCAGAGGAAAT	[22]
Il-10	CATGGCCCAGAAATCAAGGA	GGAGAAATCGATGACAGCGC	[22]
Il-1β	AAATACCTGTGGCCTTGGGC	CTTGGGATCCACACTCTCCAG	[10]
Tnf-α	GCCACCACGCTCTTCTGCCT	GGCTGATGGTGTGGGTGAGG	[10]
Cnr1	TGAAGTCGATCTTAGACGGCC	GTGGTGATGGTACGGAAGGTA	[23]
Cnr2	TGAATGAGCAGACCGACA GG	AGAGATGTTTGCTGG GTGGC	[23]
Trpv1	TCGTCTACCTCGTGTTCTTGTTTG	CCAGATGTTCTTGCTCTCTTGTGC	[24]
Gapdh	TCACCACCATGGAGAAGGC	GCTAAGCAGTTGGTGGTGCA	[25]

Supplementary Table 2. LC-MS/MS metabolite identification

Substrate	Product	Formulae	m/z (+)<br>m/z (-)	Retention Times (Min)	Metabolite ID
NADA	Parent	$C_{28}H_{41}NO_3$	440.3159<br>438.3014	55.4	
	-O	$C_{28}H_{41}NO_4$	456.3108<br>454.2963	41.6; 42.6; 44.6; 45.6; 47.8; 49.0; 49.6; 52.6	PD1-PD8
	-O_2	$C_{28}H_{41}NO_5$	472.3057<br>470.2912	48.8<br>53.0	PD9 & PD10
	-HQ	$C_{28}H_{39}NO_4$	454.2952<br>452.2806	46.3	PD11
NA5HT	Parent	$C_{30}H_{42}N_2O_2$	463.3319<br>461.3174	55.6	
	-O	$C_{30}H_{42}N_2O_3$	479.3268<br>477.3122	42.4; 48.3; 52.4; 54.1	PS1-PS4
	-O_2	$C_{30}H_{42}N_2O_4$	495.3217<br>493.3072	50.5	PS5
	-Q	$C_{30}H_{40}N_2O_3$	477.3112<br>475.2969	54.7<br>56.8	PS6 & PS7
CAP	Parent	$C_{18}H_{27}NO_3$	306.2064<br>304.1918	39.5	
	-*O*-de-CH_3	$C_{17}H_{25}NO_3$	292.1907<br>290.1762	36.7	PC1
	-O	$C_{18}H_{27}NO_4$	322.2013<br>320.1867	25.6<br>26.6	PC2 & PC3
	-O_2	$C_{18}H_{27}NO_5$	338.1962<br>336.1816	20.3	PC4
	-HQ	$C_{17}H_{23}NO_4$	306.1700<br>304.1554	23.8	PC5

Supplementary Table 3. Binding interaction energies for AEA and NADA in Configuration 1.

**AEA**			
	**Residue**	**Interaction energy (kJ/mol)**	**Error (±kJ/mol)**
	ARG321	-8.92481	3.47937
	ARG495	-4.72929	1.37637
	ARG484	-3.76565	5.08937
	ASN185	-3.6608	0.91729
	LEU215	-3.13021	0.90826
	HIS494	-3.10337	2.63799
	HIS181	-2.99208	1.25837
	SER493	-2.98903	1.15341
	PRO491	-2.91059	2.22862
	TRP322	-2.7051	1.87051
	VAL492	-2.34359	0.69187
	LEU212	-2.29354	0.802
	THR313	-2.29139	0.656
	LEU481	-2.25677	2.00732
	LEU496	-1.88488	0.98401
	SER480	-1.76965	1.92706
	THR219	-1.65714	0.72366
	ASP216	-1.53383	0.59384
	PHE483	-1.15568	1.16863
**NADA**			
	**Residue**	**Interaction energy (kJ/mol)**	**Error (±kJ/mol)**
	ILE489	-3.25625	0.96471
	GLU222	-2.81904	7.20851
	THR114	-2.64145	1.26045
	ILE127	-2.4887	0.672
	THR488	-2.42929	1.62007
	PHE310	-2.33006	0.68078
	ASN231	-2.13378	1.40423
	ILE487	-2.12379	1.17521
	ARG117	-2.0808	1.03731
	MET400	-2.0443	0.97651
	ILE86	-1.86892	0.99568
	VAL380	-1.86384	0.53806
	ALA311	-1.75345	0.60574
	MET128	-1.71349	0.69304
	PRO381	-1.52613	0.65021

	LEU83	-1.51171	0.79452
	GLN228	-1.16917	1.08287
	GLU314	-1.15822	0.47136
	THR315	-0.98946	0.40637

Supplementary Table 4. Binding interaction energies for AEA and NADA in Configuration 2.

**AEA**			
	**Residue**	**Interaction energy (kJ/mol)**	**Error (±kJ/mol)**
	ILE487	-5.89876	1.21444
	GLN228	-5.83744	2.55033
	GLY486	-4.4337	0.85385
	ILE489	-3.73012	1.80267
	THR488	-2.86717	1.23994
	LEU83	-2.66672	1.22142
	PRO381	-2.01531	0.5883
	MET400	-1.92187	0.59975
	ARG111	-1.86332	0.65592
	PHE61	-1.76106	0.70389
	PRO112	-1.56523	0.63967
	THR114	-1.38301	0.59581
	VAL380	-1.31937	0.42989
	LEU81	-1.29664	0.75077
	ILE86	-1.23497	0.69801
	ASN231	-1.23156	0.70985
	GLU82	-1.21236	0.75533
	ILE127	-0.93357	0.4817
	MET485	-0.90994	0.50495
**NADA**			
	**Residue**	**Interaction energy (kJ/mol)**	**Error (±kJ/mol)**
	ASP307	-24.8669	9.39773
	PHE310	-5.45818	1.08501
	SER490	-4.84377	2.57133
	ILE489	-3.81991	1.27441
	ALA311	-2.55907	0.64457
	GLU222	-2.43978	6.04616
	THR315	-2.4274	0.79848
	GLU314	-1.99078	0.60895
	ILE127	-1.74117	0.56921
	VAL380	-1.56889	0.67457
	ILE376	-1.45199	0.64053
	ILE375	-1.40588	0.55081
	THR318	-1.24705	0.4895
	TRP251	-1.15856	0.49279
	PHE121	-1.06322	0.47599

	THR219	-0.99602	0.86035
	PRO491	-0.87034	0.55172
	LEU126	-0.76823	0.67118
	ARG111	-0.72046	0.51937

Supplementary Table 5. Binding interaction energies for AEA and NA5HT in Configuration 1.

AEA			
	**Residue**	**Interaction energy (kJ/mol)**	**Error (±kJ/mol)**
	ARG321	-21.757	3.21232
	TRP322	-4.69984	1.39869
	PRO491	-4.12342	1.47182
	ARG495	-3.77759	1.6088
	VAL492	-3.66875	0.78594
	SER493	-2.94951	0.78101
	ASN185	-2.82361	0.97184
	THR318	-2.74648	0.88867
	LEU215	-2.29793	0.59603
	LEU496	-2.1508	0.48536
	THR219	-1.81981	0.54936
	ASP216	-1.45737	0.6174
	THR313	-1.44611	0.63856
	HSD181	-1.23107	0.92312
	LEU212	-1.22474	0.5249
	SER317	-1.13683	0.42019
	SER480	-1.12392	0.50546
	SER490	-1.05437	0.5769
	LEU325	-0.94713	0.42667
**NA5HT**			
	**Residue**	**Interaction energy (kJ/mol)**	**Error (±kJ/mol)**
	GLU222	-5.78558	3.11688
	THR488	-3.52051	2.94825
	ILE489	-3.40178	1.07731
	VAL380	-3.0252	0.61197
	GLN228	-3.00795	2.10628
	ARG117	-2.86239	1.37033
	THR114	-2.8041	0.76497
	ARG111	-2.58118	0.899
	ILE127	-2.16936	0.67201
	ASN231	-1.96704	0.91609
	PRO112	-1.8457	0.72025
	PHE310	-1.78344	0.73298
	ALA311	-1.49405	0.50818
	PRO381	-1.43143	0.66267
	SER490	-1.38003	0.99802

	MET400	-1.26626	0.51045
	ILE487	-1.22545	1.01001
	THR315	-1.11141	0.64492
	GLY486	-1.00512	0.58716

Supplementary Table 6. Binding interaction energies for AEA and NA5HT in Configuration 2.

**AEA**			
	**Residue**	**Interaction energy (kJ/mol)**	**Error (±kJ/mol)**
	GLN228	-6.5189	1.61495
	ILE487	-6.4654	1.07545
	GLY486	-5.05945	0.69118
	ASN231	-3.30921	1.13293
	THR488	-2.75129	0.70766
	MET400	-2.60969	0.64574
	LEU83	-2.51603	0.82828
	ILE86	-2.41382	0.70615
	ILE489	-2.06282	0.60377
	PHE61	-1.61387	0.53751
	GLU222	-1.59248	1.60739
	LEU81	-1.54611	0.58844
	SER490	-1.37099	0.95213
	GLU82	-1.32141	0.53585
	ALA88	-1.07485	0.34869
	MET485	-1.04278	0.39769
	ALA223	-1.02855	0.51492
	THR114	-1.0283	0.63714
	VAL232	-0.97153	0.50219
**NA5HT**			
	**Residue**	**Interaction energy (kJ/mol)**	**Error (±kJ/mol)**
	ARG117	-4.62722	1.01273
	PHE310	-4.25033	0.78785
	ILE489	-3.79065	0.7503
	PRO112	-3.53451	2.60917
	ILE127	-3.16155	0.66233
	VAL380	-3.0195	0.73603
	ARG111	-2.8175	0.86368
	ALA311	-2.54837	0.69605
	GLU314	-2.41186	0.5614
	THR315	-2.06731	0.79649
	THR114	-1.5682	0.61104
	ILE376	-1.48917	0.57733
	THR318	-1.26557	0.42192
	ILE375	-1.19388	0.4528
	GLU222	-0.95049	0.97809

	VAL113	-0.87465	0.51344
	SER490	-0.83479	0.43952
	THR488	-0.62754	0.11655
	PRO491	-0.60725	0.5804

Supplemental Movies

Supplementary Movie 1. *Molecular dynamics (MD) simulations of NADA and AEA in Configuration 1.* MD simulations were performed for 50 ns as described in the Methods section. In this configuration, AEA is bound in the PUFA binding pocket and NADA in the substrate access channel. Green spheres represent the 14' and 15' positions of NADA.

Supplementary Movie 2. *Molecular dynamics (MD) simulations of NADA and AEA in Configuration 2.* MD simulations were performed for 50 ns as described in the Methods section. In this configuration, AEA is bound at the entrance of the substrate access channel and stabilizes the binding of NADA near the heme. Green spheres represent the 14' and 15' positions of NADA.

Supplementary Movie 3. *Molecular dynamics (MD) simulations of NA5HT and AEA in Configuration 1.* MD simulations were performed for 50 ns as described in the Methods section. In this configuration, AEA is bound in the PUFA binding pocket and NA5HT in the substrate access channel. Green spheres represent the 14' and 15' positions of NA5HT.

Supplementary Movie 4. *Molecular dynamics (MD) simulations of NA5HT and AEA in Configuration 2.* MD simulations were performed for 50 ns as described in the Methods section. In this configuration, AEA is bound at the entrance of the substrate access channel and stabilizes the binding of NA5HT near the heme. Green spheres represent the 14' and 15' positions of NA5HT.

Supplementary References

1 McDougle, D. R., Palaria, A., Magnetta, E., Meling, D. D. & Das, A. Functional studies of N-terminally modified CYP2J2 epoxygenase in model lipid bilayers. *Protein Sci* **22**, 964-979, doi:10.1002/pro.2280 (2013).

2 Schmittgen, T. D. & Livak, K. J. Analyzing real-time PCR data by the comparative C(T) method. *Nature protocols* **3**, 1101-1108 (2008).

3 McDougle, D. R. *et al.* Anti-inflammatory omega-3 endocannabinoid epoxides. *Proc Natl Acad Sci U S A* **114**, E6034-E6043, doi:10.1073/pnas.1610325114 (2017).

4 Zelasko, S., Palaria, A. & Das, A. Optimizations to achieve high-level expression of cytochrome P450 proteins using Escherichia coli expression systems. *Protein Expres Purif* **92**, 77-87, doi:DOI 10.1016/j.pep.2013.07.017 (2013).

5 Arnold, W. R., Baylon, J. L., Tajkhorshid, E. & Das, A. Arachidonic Acid Metabolism by Human Cardiovascular CYP2J2 Is Modulated by Doxorubicin. *Biochemistry* **56**, 6700-6712, doi:10.1021/acs.biochem.7b01025 (2017).

6 Arnold, W. R., Weigle, A. T. & Das, A. Cross-talk of cannabinoid and endocannabinoid metabolism is mediated via human cardiac CYP2J2. *J Inorg Biochem* **184**, 88-99, doi:10.1016/j.jinorgbio.2018.03.016 (2018).

7 Arnold, W. R., Baylon, J. L., Tajkhorshid, E. & Das, A. Asymmetric Binding and Metabolism of Polyunsaturated Fatty Acids (PUFAs) by CYP2J2 Epoxygenase. *Biochemistry* **55**, 6969-6980, doi:10.1021/acs.biochem.6b01037 (2016).

8 Capdevila, J. H. *et al.* The highly stereoselective oxidation of polyunsaturated fatty acids by cytochrome P450BM-3. *J Biol Chem* **271**, 22663-22671 (1996).

9 Hu, S. S. *et al.* The biosynthesis of N-arachidonoyl dopamine (NADA), a putative endocannabinoid and endovanilloid, via conjugation of arachidonic acid with dopamine. *Prostaglandins, leukotrienes, and essential fatty acids* **81**, 291-301, doi:10.1016/j.plefa.2009.05.026 (2009).

10 Marinelli, S. *et al.* N-arachidonoyl-dopamine tunes synaptic transmission onto dopaminergic neurons by activating both cannabinoid and vanilloid receptors. *Neuropsychopharmacol* **32**, 298-308, doi:10.1038/sj.npp.1301118 (2007).

11 Huang, S. M. *et al.* An endogenous capsaicin-like substance with high potency at recombinant and native vanilloid VR1 receptors. *Proc Natl Acad Sci U S A* **99**, 8400-8405, doi:10.1073/pnas.122196999

99/12/8400 [pii] (2002).

12 Siller, M. *et al.* Oxidation of endogenous N-arachidonoylserotonin by human cytochrome P450 2U1. *Journal of Biological Chemistry* **289**, 10476-10487, doi:10.1074/jbc.M114.550004

M114.550004 [pii] (2014).

13 Verhoeckx, K. C. *et al.* Presence, formation and putative biological activities of N-acyl serotonins, a novel class of fatty-acid derived mediators, in the intestinal tract. *Biochim Biophys Acta* **1811**, 578-586, doi:10.1016/j.bbalip.2011.07.008

S1388-1981(11)00133-8 [pii] (2011).

14 Buczynski, M. W. & Parsons, L. H. Quantification of brain endocannabinoid levels: methods, interpretations and pitfalls. *Br J Pharmacol* **160**, 423-442, doi:10.1111/j.1476-5381.2010.00787.x

BPH787 [pii] (2010).

15 Schmid, P. C. *et al.* Occurrence and postmortem generation of anandamide and other long-chain N-acylethanolamines in mammalian brain. *Febs Lett* **375**, 117-120 (1995).

16 Napolitano, A., Pezzella, A. & Prota, G. New reaction pathways of dopamine under oxidative stress conditions: nonenzymatic iron-assisted conversion to norepinephrine and the neurotoxins 6-hydroxydopamine and 6, 7-dihydroxytetrahydroisoquinoline. *Chem Res Toxicol* **12**, 1090-1097, doi:tx990079p [pii] (1999).

17 Napolitano, A., Crescenzi, O., Pezzella, A. & Prota, G. Generation of the neurotoxin 6-hydroxydopamine by peroxidase/H2O2 oxidation of dopamine. *J Med Chem* **38**, 917-922 (1995).

18 Lam, P. M. *et al.* Activation of recombinant human TRPV1 receptors expressed in SH-SY5Y human neuroblastoma cells increases [Ca(2+)](i), initiates neurotransmitter release and promotes delayed cell death. *Journal of neurochemistry* **102**, 801-811, doi:10.1111/j.1471-4159.2007.04569.x (2007).

19 Roy, J., Watson, J. E., Hong, I., Fan, T. M. & Das, A. Anti-Tumorigenic Properties of Omega-3 Endocannabinoid Epoxides. *J Med Chem*, doi:10.1021/acs.jmedchem.8b00243 (2018).

20 Pertwee, R. G. Pharmacology of cannabinoid receptor ligands. *Curr Med Chem* **6**, 635-664 (1999).

21 Graves, J. P. *et al.* Quantitative Polymerase Chain Reaction Analysis of the Mouse Cyp2j Subfamily: Tissue Distribution and Regulation. *Drug Metab Dispos* **43**, 1169-1180, doi:10.1124/dmd.115.064139 (2015).

22 Oh, D. Y. *et al.* GPR120 Is an Omega-3 Fatty Acid Receptor Mediating Potent Anti-inflammatory and Insulin-Sensitizing Effects. *Cell* **142**, 687-698, doi:10.1016/j.cell.2010.07.041 (2010).

23 Zhang, M. *et al.* Modulation of the balance between cannabinoid CB1 and CB2 receptor activation during cerebral ischemic/reperfusion injury. *Neuroscience* **152**, 753-760, doi:10.1016/j.neuroscience.2008.01.022 (2008).

24 Phan, T. X., Ton, H. T., Chen, Y., Basha, M. E. & Ahern, G. P. Sex-dependent expression of TRPV1 in bladder arterioles. *Am J Physiol-Renal* **311**, F1063-F1073, doi:10.1152/ajprenal.00234.2016 (2016).

25 Yen, C. H. *et al.* Characterization of a new murine cell line of sarcomatoid hepatocellular carcinoma and its application for biomarker/therapy development. *Sci Rep* **7**, 3052, doi:10.1038/s41598-017-03164-3 (2017).
