# Supplementary material for: Anti-inflammatory dopamine- and serotonin-based endocannabinoid epoxides reciprocally regulate cannabinoid receptors and the TRPV1 channel
Source: Nat Commun. 2021 Feb 10;12:926. doi: 10.1038/s41467-021-20946-6 (PMC7876028; doi:10.1038/s41467-021-20946-6)
Supplement: Supplementary file 2 — Description of Additional Supplementary Files [file 41467_2021_20946_MOESM2_ESM.docx]

**Description of Additional Supplementary Files**

**Supplementary Movie 1.** Molecular dynamics (MD) simulations of NADA and AEA in Configuration 1. MD simulations were performed for 50 ns as described in the Methods section. In this configuration, AEA is bound in the PUFA binding pocket and NADA in the substrate access channel. Green spheres represent the 14’ and 15’ positions of NADA.

**Supplementary Movie 2.** Molecular dynamics (MD) simulations of NADA and AEA in

Configuration 2. MD simulations were performed for 50 ns as described in the Methods section. In this configuration, AEA is bound at the entrance of the substrate access channel and stabilizes the binding of NADA near the heme. Green spheres represent the 14’ and 15’ positions of NADA.

**Supplementary Movie 3.** Molecular dynamics (MD) simulations of NA5HT and AEA in Configuration 1. MD simulations were performed for 50 ns as described in the Methods section. In this configuration, AEA is bound in the PUFA binding pocket and NA5HT in the substrate access channel. Green spheres represent the 14’ and 15’ positions of NA5HT.

**Supplementary Movie 4.** Molecular dynamics (MD) simulations of NA5HT and AEA in Configuration 2. MD simulations were performed for 50 ns as described in the Methods section. In this configuration, AEA is bound at the entrance of the substrate access channel and stabilizes the binding of NA5HT near the heme. Green spheres represent the 14’ and 15’ positions of NA5HT.
