# Supplementary figures and images for: Anti-inflammatory dopamine- and serotonin-based endocannabinoid epoxides reciprocally regulate cannabinoid receptors and the TRPV1 channel
Source: Nat Commun. 2021 Feb 10;12:926. doi: 10.1038/s41467-021-20946-6 (PMC7876028; doi:10.1038/s41467-021-20946-6)

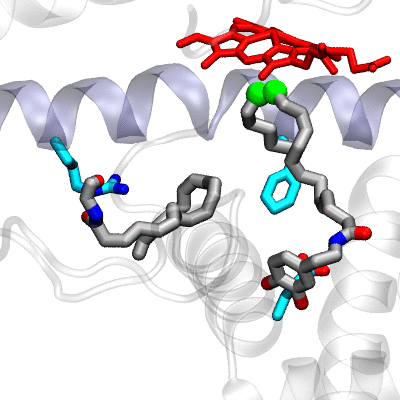

Supplement: Supplementary file 3 — Supplementary Movie 1 [file 41467_2021_20946_MOESM3_ESM.gif]

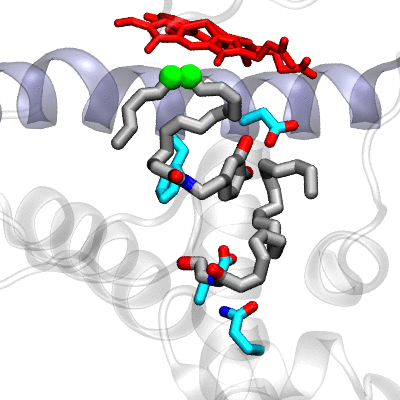

Supplement: Supplementary file 4 — Supplementary Movie 2 [file 41467_2021_20946_MOESM4_ESM.gif]

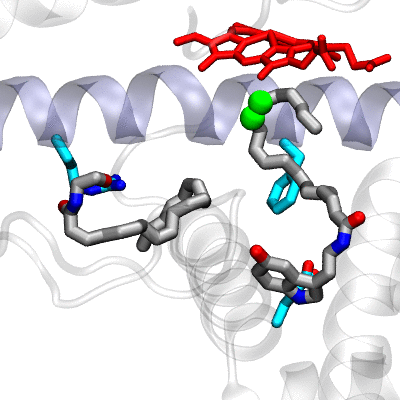

Supplement: Supplementary file 5 — Supplementary Movie 3 [file 41467_2021_20946_MOESM5_ESM.gif]

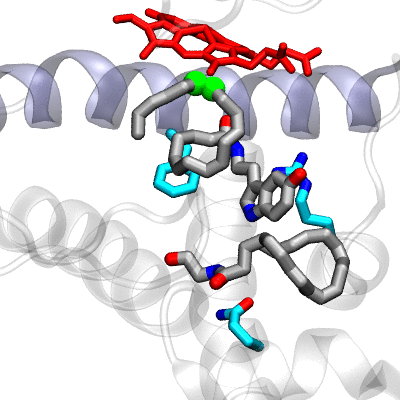

Supplement: Supplementary file 6 — Supplementary Movie 4 [file 41467_2021_20946_MOESM6_ESM.gif]
