# Supplementary material for: Anti-inflammatory dopamine- and serotonin-based endocannabinoid epoxides reciprocally regulate cannabinoid receptors and the TRPV1 channel
Source: Nat Commun. 2021 Feb 10;12:926. doi: 10.1038/s41467-021-20946-6 (PMC7876028; doi:10.1038/s41467-021-20946-6)
Supplement: Supplementary file 7 — Reporting Summary [file 41467_2021_20946_MOESM7_ESM.pdf]

nature research

Corresponding author(s): Aditi Das

Last updated by author(s): October 1st 2020

Reporting Summary

Nature Research wishes to improve the reproducibility of the work that we publish. This form provides structure for consistency and transparency in reporting. For further information on Nature Research policies, see Authors & Referees and the Editorial Policy Checklist.

Statistics

For all statistical analyses, confirm that the following items are present in the figure legend, table legend, main text, or Methods section.

n/a	Confirmed	
☐	☒	The exact sample size (*n*) for each experimental group/condition, given as a discrete number and unit of measurement
☐	☒	A statement on whether measurements were taken from distinct samples or whether the same sample was measured repeatedly
☐	☒	The statistical test(s) used AND whether they are one- or two-sided<br>*Only common tests should be described solely by name; describe more complex techniques in the Methods section.*
☒	☐	A description of all covariates tested
☒	☐	A description of any assumptions or corrections, such as tests of normality and adjustment for multiple comparisons
☐	☒	A full description of the statistical parameters including central tendency (e.g. means) or other basic estimates (e.g. regression coefficient) AND variation (e.g. standard deviation) or associated estimates of uncertainty (e.g. confidence intervals)
☐	☒	For null hypothesis testing, the test statistic (e.g. *F*, *t*, *r*) with confidence intervals, effect sizes, degrees of freedom and *P* value noted<br>*Give P values as exact values whenever suitable.*
☒	☐	For Bayesian analysis, information on the choice of priors and Markov chain Monte Carlo settings
☒	☐	For hierarchical and complex designs, identification of the appropriate level for tests and full reporting of outcomes
☒	☐	Estimates of effect sizes (e.g. Cohen's *d*, Pearson's *r*), indicating how they were calculated

Our web collection on statistics for biologists contains articles on many of the points above.

Software and code

Policy information about availability of computer code

Data collection	Software Analyst 1.6.2, Mass Lynx 3.4, Origin Pro 9.1, Excel 2016, Mnova v.12, VMD 1.9.2, NAMD2, Auto Dock Vina, NIS-Elements Imaging, Clampex 10.4 software
Data analysis	Software Analyst 1.6.2, Origin Pro 9.1, Excel 2016, Mnova v.12, VMD 1.9.2, NAMD2, Auto Dock Vina, NIS Elements Imaging, Clampex 10.4 Software

For manuscripts utilizing custom algorithms or software that are central to the research but not yet described in published literature, software must be made available to editors/reviewers. We strongly encourage code deposition in a community repository (e.g. GitHub). See the Nature Research guidelines for submitting code & software for further information.All data generated or analyzed during this study are included in this published article (and its supplementary information files). Other material is available from the corresponding author on reasonable request. Material availability statement: All plasmids used in the study are available from Addgene or can be obtained from the corresponding author on request. All other data or resources are available in the Source Data file and source data supplementary file or from the corresponding author upon reasonable request.

Policy information about availability of data

All manuscripts must include a data availability statement. This statement should provide the following information, where applicable:

- Accession codes, unique identifiers, or web links for publicly available datasets
- A list of figures that have associated raw data
- A description of any restrictions on data availability

All data generated or analysed during this study are included in this published article (and its supplementary information files). Whatever is not included is available from the corresponding author on reasonable request.

Field-specific reporting

Please select the one below that is the best fit for your research. If you are not sure, read the appropriate sections before making your selection.

☒ Life sciences ☐ Behavioural & social sciences ☐ Ecological, evolutionary & environmental sciences

Life sciences study design

All studies must disclose on these points even when the disclosure is negative.

Sample size	We described all sample sizes, included when obtained and biological vs. technical, are included in the figure legends and Methods section. Sample sizes dependent on the experiment used. For biochemical experiment N=3=6 and for cell culture and animal work n>3 in all cases
Data exclusions	Data was excluded based on a Grubb's test and whether the experiment did not work based on controls
Replication	We describe the replication of the data, including when obtained, in the figures and Methods section
Randomization	Randomization was applied to the DRG neurons study where they were isolated from animals
Blinding	Binding is application to the DRG neuron study where they were isolated from animals.

Reporting for specific materials, systems and methods

We require information from authors about some types of materials, experimental systems and methods used in many studies. Here, indicate whether each material, system or method listed is relevant to your study. If you are not sure if a list item applies to your research, read the appropriate section before selecting a response.

Materials & experimental systems

n/a	Involved in the study	
☐	☒	Antibodies
☐	☒	Eukaryotic cell lines
☒	☐	Palaeontology
☐	☒	Animals and other organisms
☒	☐	Human research participants
☒	☐	Clinical data

Methods

n/a	Involved in the study	
☒	☐	ChIP-seq
☒	☐	Flow cytometry
☒	☐	MRI-based neuroimaging

Eukaryotic cell lines

Policy information about cell lines

Cell line source(s)	BV-2 microglia cells were a gift from Dr. Rodney Johnson (University of Illinois at Urbana-Champaign). HTLA cells for PRESTO-tango assay were obtained from Brian Roth's Lab at PDSP, University of North Carolina – Chapel Hill. HEK-TRPV1 was obtained from Heather Bradshaw, Indiana University Bloomington
Authentication	All the cell lines were obtained from authentic sources as described.
Mycoplasma contamination	The cell lines were not tested for mycoplasma
Commonly misidentified lines (See ICLAC register)	No cell line from ICLAC was used
